# Forests in protected areas worldwide face increased thermal pressure from urbanization

**DOI:** 10.1038/s41467-026-76226-8

**Published:** 2026-07-28

**Authors:** Anqi Huang, Xiyan Xu, Gensuo Jia, Elise Belle, Pieter De Frenne, Yuyu Zhou

**Affiliations:** 1 Key Laboratory for Climate Risk and Urban-Rural Smart Governance, School of Geography, Jiangsu Second Normal University, Nanjing, China; 2 State Key Laboratory of Earth System Numerical Modeling and Application, Institute of Atmospheric Physics, Chinese Academy of Sciences, Beijing, China; 3 Collaborative Innovation Center on Forecast and Evaluation of Meteorological Disasters (CIC-FEMD), Nanjing University of Information Science & Technology, Nanjing, China; 4 United Nations Environment Programme World Conservation Monitoring Centre (UNEP-WCMC), Cambridge, UK; 5 Forest & Nature Lab, Department of Environment, Ghent University, Gontrode-Melle, Belgium; 6 Institute for Climate and Carbon Neutrality, The University of Hong Kong, Hong Kong SAR, China; 7 Department of Geography, The University of Hong Kong, Hong Kong SAR, China

**Keywords:** Climate sciences, Ecology, Forestry

## Abstract

Protected areas (PAs), thermal refuges for biodiversity, are increasingly under pressure from urbanization. Gaps remain in how urbanization worldwide influences the thermal environment of PAs. Here, we quantify urban pressure on protected forests as a distance-weighted built-up intensity. The thermal impacts of urban pressure are assessed by comparing satellite-derived mean land surface temperature (LST) and thermal extremes inferred by the 90th-percentile maximum LST of protected forests within and beyond a 5-km buffer zone outside urban boundaries. We show that the global mean distance between urban areas and PAs has decreased by over one-third (12.82 km) since 1990, increasing urban pressure on protected forests by about 2.56%. Urbanization alters thermal environments by elevating LST, and the frequency and intensity of thermal extremes, with the highest impact on annual mean daily LST (0.63 °C) and intensity of thermal extremes (0.81 °C) in tropical protected forests. We highlight the need to integrate urban development and conservation planning to support biodiversity conservation and sustainable development.

## Abstract (web summary)

A global analysis shows that since 1990, the average distance between urban areas and protected areas has decreased by over one-third, causing increased warming and extreme heat in forests within these PAs.

## Introduction

The number of protected areas (PAs) in the world has grown dramatically since their creation in the nineteenth century. In 1950, less than 0.5% of the global land surface was protected^1^. Today, PAs cover more than 16% of the global terrestrial areas^2^. The Kunming-Montreal Global Biodiversity Framework (GBF) aims to expand PA coverage to 30% by 2030 to reduce biodiversity loss, a target widely known as 30 × 30. PAs are crucial for biodiversity conservation and carbon sequestration, recognized as nature-based solutions to climate change adaptation and mitigation^3^. In particular, forest conservation is considered of paramount importance^4–7^. Protected forests buffer local climate through biophysical effects by altering surface physical features such as albedo, evapotranspiration, and aerodynamic resistance^8–10^. These processes create a more stable microclimate (i.e., local climatic conditions) within PAs, providing thermal refuges that protect species from macroclimate (i.e., large-scale climatic conditions) warming^11–13^. Tropical protected forests tend to exhibit stronger local cooling, whereas boreal protected forests play a greater role in slowing local warming trends^11^. With large-scale afforestation efforts underway, such as the United Nations Strategic Plan for Forests (UNSPF), which aims to increase global forest area by 3% by 2030, and the Bonn Challenge, which targets the restoration of 350 million hectares of degraded and deforested land globally by 2030^14,15^, it is increasingly important to assess whether protected forests across different latitudes can effectively maintain their thermal buffering capability.

The effectiveness of protected forests as thermal buffering for local climate relies on their effective management and isolation from human disturbances^11^. Observational evidence suggested that proper management can effectively maintain forest structural integrity and enhance canopy shading and evaporative cooling^16,17^. Although originally established in remote locations for optimal conservation, one-third of global PAs now face intense human activities because of other land uses, such as expanding agriculture and urbanization^18,19^. As urbanization approaches PAs, residents have easier access to ecosystem services, such as clean air and water, as well as cultural services obtained through recreation and environmental education^20–22^. The ease of access to ecosystem services also fosters policy support and financial resources, thereby enhancing the effectiveness of conservation efforts^23,24^. However, the intensified human activities induced by urbanization have also exacerbated logging pressure^25^, habitat fragmentation^26^, and disrupted canopy structure^27^ near or in PAs. These human-induced changes in protected forests modify surface energy budgets and are often linked to local temperature anomalies^28^. As a result, protected forests tend to experience increased microclimate instability^29^, weakening their capacity to serve as thermal refuges for biodiversity.

Many species depend on microclimates sustained by natural landscapes, including thermally sensitive ectotherms such as amphibians and reptiles that rely on shaded, cooler habitats to avoid thermal stress^30,31^. PAs, by conserving intact natural landscapes, provide thermal refuges for these species under climate change^16,32^, but this role is increasingly undermined by urbanization. Urbanization transforms natural landscapes into impervious surfaces such as concrete and asphalt, which enhances the absorption of solar radiation by the surface and reduces surface evaporative cooling, leading to warming in urban areas relative to rural areas, referred to as the urban heat island (UHI) effect^33,34^. As urbanization increases, the thermal stress spreads beyond urban boundaries due to the spillover of extra warmth of UHI and anthropogenic heat release^35–37^. This urban heat spillover, driven by transport of urban heat through atmospheric advection and diffusion^38,39^, intensifies thermal stress on species in PAs, while urbanization-induced habitat destruction and fragmentation reduce habitat connectivity and constrain climate-driven dispersal, together increasing the risk of local extinctions^40^. Despite growing recognition of urban heat spillover, existing studies mainly focused on thermal conditions in human-dominated areas^36,41^, whereas the magnitude and spatial extent of urban heat spillover into natural and semi-natural ecosystems remain poorly quantified.

**Fig. 1:**
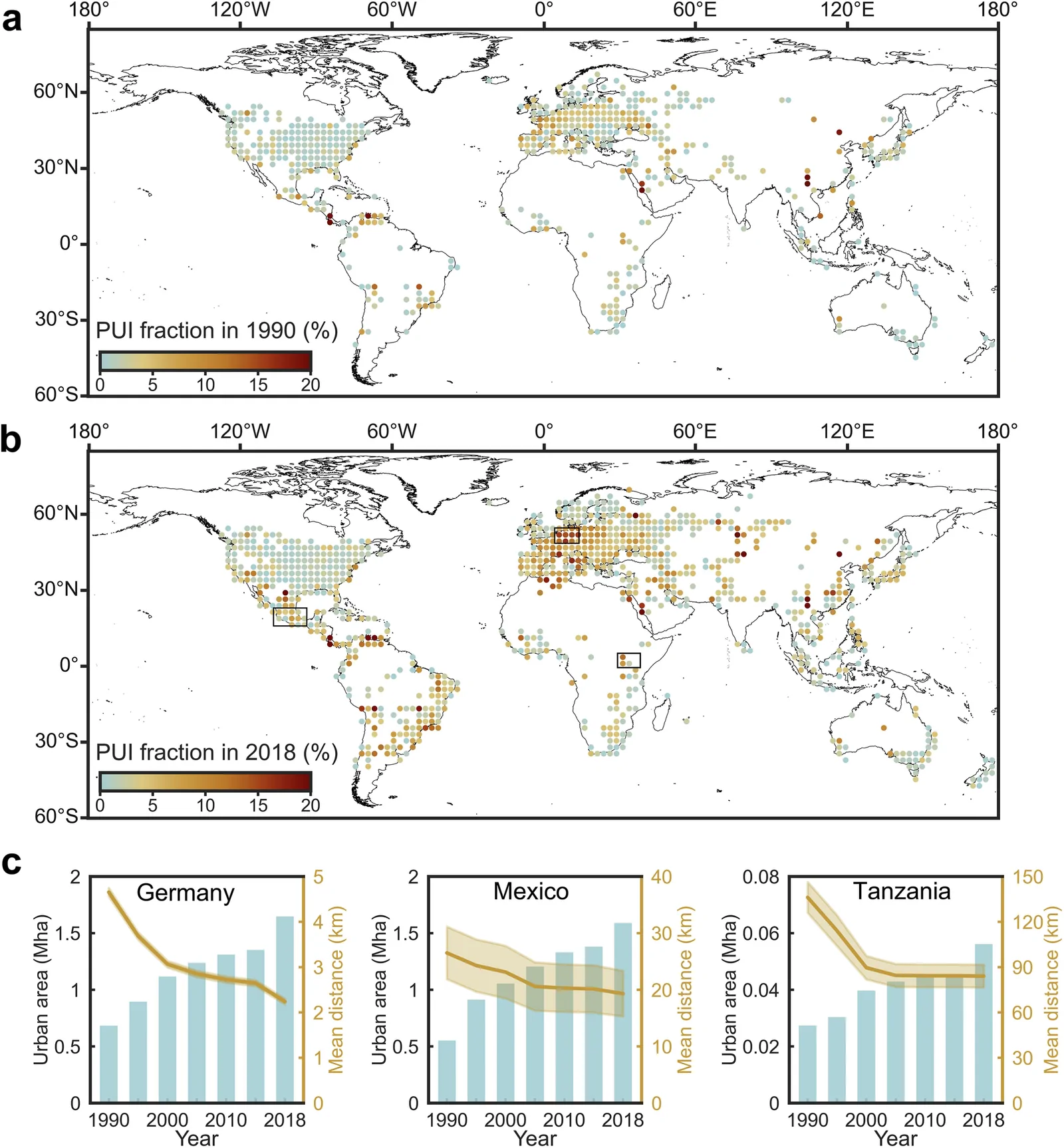
Global protected area-urban interfaces. **a**, **b** the area fraction of protected area-urban interfaces (PUIs) per 2.5° window in 1990 and 2018, and **c** examples of urban expansion and its mean distance to protected areas in countries with different PUI fraction levels in 2018: Germany (12.85%), Mexico (5.13%), and Tanzania (2.40%). The PUI is identified as the transition zone between PAs and urban areas, in which habitats are under the combined effects of nature conservation and urbanization. In (**c**), lines represent mean distances to protected areas, and yellow shading indicates ± standard error of the mean (SEM). Sample sizes were *n* = 2562, 38, and 50 PAs for Germany, Mexico, and Tanzania, respectively.

In this work, we firstly define a concept of protected area-urban interfaces (PUIs) as transition zones between the PAs and urban areas, mapped as the overlapping regions between PAs and urban buffer zones delineated at distances of 5, 10, 25, and 50 kilometers (km) from urban boundaries (“Methods”: Identification of PAs, urban areas, and their interfaces). The portions of PAs excluding PUIs and sharing the same climatic background are defined as background PAs (Supplementary Fig. 1). By comparing protected forest habitats in PUIs and background PAs, we use multi-source satellite observations to quantify the urban pressure on protected forests as a distance-weighted built-up intensity, and then investigate the impact of urban pressure on the local thermal environment of protected forest habitats globally. We show that from 1990 to 2018, the area of global PUIs within 5 km of urban areas increased over six-fold (Fig. 1). Forests in PUIs are consistently exposed to higher urban pressure and warmer mean land surface temperature than those in background PAs, with the highest warming impact on the mean land surface temperature and intensity of thermal extremes in the tropical protected forests. Our findings underscore the need to integrate urban planning with biodiversity conservation in global sustainability frameworks.

## Results

### Increased urban pressure on protected forest habitats

We present a global assessment of the urban pressure on protected forest habitats by comparing differences in urban pressure between forest habitats in PUIs and PAs between 1990 and 2018. Urban pressure on forest habitats is quantified as a function of built-up intensity and distance surrounding each forest habitat pixel (“Methods”: Quantification of urban pressure on forest habitats). More than 95% of PAs established before 1990 have experienced approaching urban expansion between 1990 and 2018, with the global mean distance between urban areas and PAs shrinking by more than a third (Supplementary Note 1, Supplementary Fig. 2). As urbanization proceeds, urban boundaries increase, thereby expanding the interfaces between urban areas and PAs (PUIs). These expanding PUI areas are mostly concentrated in central Europe and the eastern United States, where PAs are located close to urban areas. Central Europe contributed a substantial share of the global PUI increase, while the eastern United States experienced a broad but less intense expansion of PUIs.

**Fig. 2:**
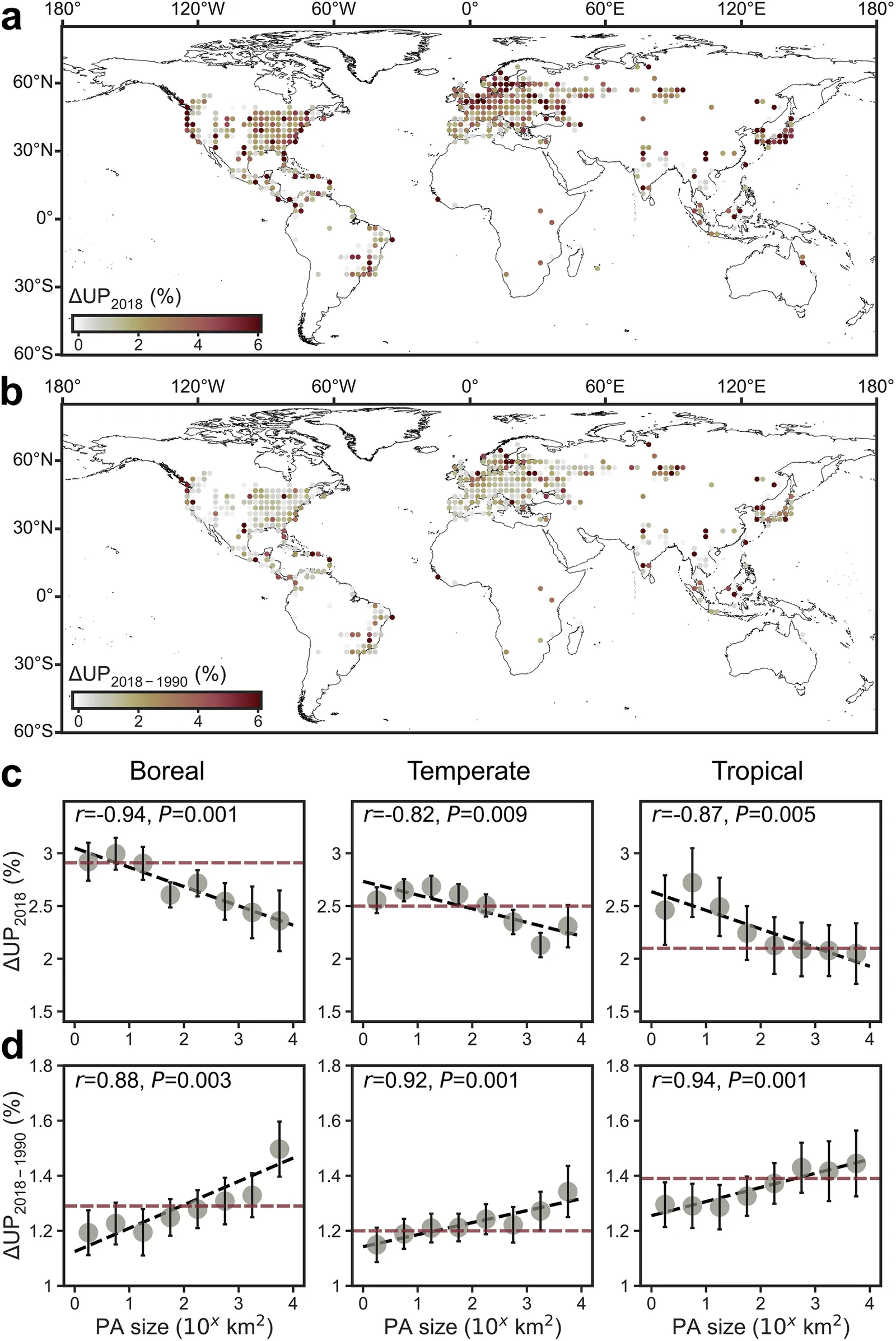
Increased urban pressure on protected forests during 1990-2018. **a** The urban pressure difference (ΔUP) between forest habitats in protected area (PA)-urban interfaces (PUIs) and background PAs in 2018 (ΔUP_2018_), **b** difference in ΔUP between forest habitats in PUIs and background PA between 1990 and 2018 (ΔUP_2018-1990_), **c** ΔUP of protected boreal, temperate, and tropical forests with different PA sizes and **d** ΔUP difference of protected boreal, temperate, and tropical forests with different PA sizes. **a**, **b** are displayed at a spatial aggregation of 2.5° resolution for clarity. In (**c**, **d**), points show mean values and error bars indicate ± standard error of the mean (SEM). Dashed red lines indicate the overall mean across all observations, and dashed black lines show linear regression trends. Correlations were assessed using two-sided Pearson correlation tests. The displayed *r* and *P* values represent the Pearson correlation coefficient and *p*-value, respectively. Sample sizes were *n* = 1007, 2383, and 395 valid observations for boreal, temperate, and tropical forests, respectively.

In 2018, more than 95% of forest habitats in 5 km PUIs were subject to greater urban pressure than in background PAs (ΔUP = UP_PUI_- UP_PAB_ > 0), with an average ΔUP of 2.56 ± 0.05% (mean ± SEM) (Fig. 2a). Urban expansion is accompanied by increased human activities, which can be quantified as human footprint intensity. The human footprint intensity is calculated as a composite measure of human activities, including the built environment, population density, nighttime lights, cropland, pasture, railways, roads, and navigable waterways, which are known to drive habitat loss and fragmentation, disrupt species dispersal, and promote the establishment of invasive species^42^. We found that human footprint intensity in PUIs is 8.43 ± 0.02% higher than that in background PAs, and urban pressure significantly increases with human footprint intensity, demonstrating widespread human activities on forest habitats in PUIs caused by urbanization (Supplementary Fig. 3). The closer PUIs are to urban areas, the greater urban pressure (Supplementary Table 1). Forest habitats experience less urban pressure in the tropical PUIs (2.10 ± 0.13%) than in the temperate (2.50 ± 0.06%) and boreal (2.91 ± 0.05%) PUIs (Fig. 2c).

From 1990 to 2018, more than 90% of forest habitats in PUIs showed a greater increase in urban pressure compared to forest habitats in background PAs (ΔUP_2018_-ΔUP_1990_ > 0), which is the greatest in the tropical region (1.39 ± 0.05%), followed by the boreal (1.29 ± 0.11%) and temperate (1.20 ± 0.04%) regions (Fig. 2b), mainly driven by rising urbanization intensity within pre-existing urban areas. Moreover, the differences of urban pressure increase in forest habitats between PUIs and background PAs rise significantly with PA size in the boreal (*r* = 0.88, *P* < 0.01), temperate (*r* = 0.92, *P* < 0.01), and tropical (*r* = 0.94, *P* < 0.01) regions (Fig. 2d).

### Alteration of the thermal environment in forest habitats

**Fig. 3:**
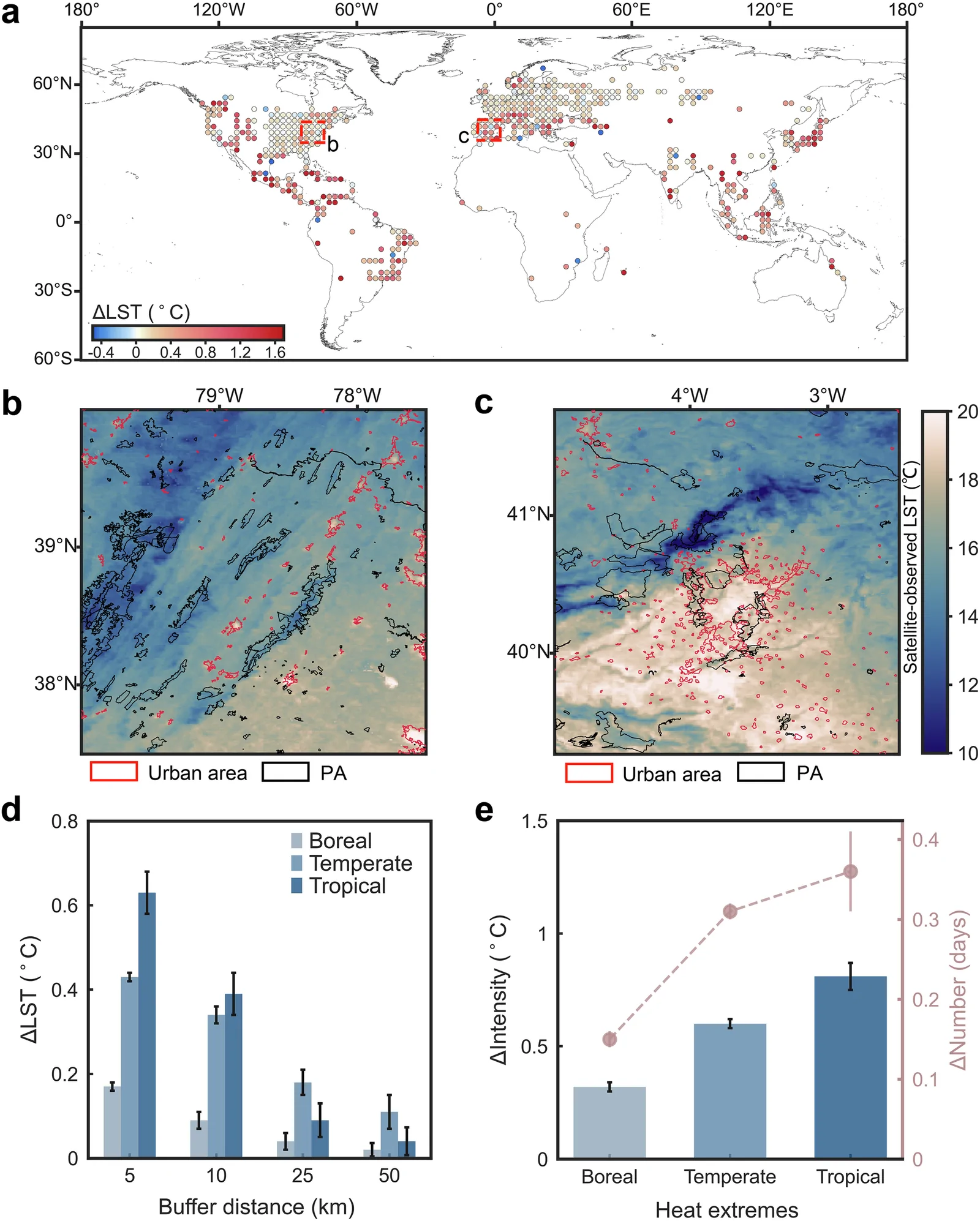
Influence of urbanization on land surface temperature of forest habitats in PUIs compared to PAs. **a** Urbanization-induced mean land surface temperature differences (ΔLST) in forest habitats in protected area (PA)-urban interfaces (PUIs) compared to that in background PAs, **b**, **c** spatial distribution of land surface temperature in urban and protected areas observed by satellite in areas of interest in the Eastern United States and Western Europe, respectively, **d** urbanization-induced mean land surface temperature anomalies in forest habitats in PUIs compared to that in background PAs with different PUI buffer distances, and **e** urbanization-induced anomalies in intensity and number of heat extremes in forest habitats in PUIs compared to that in background PAs. In (**d**, **e**), points and bars represent mean values, and error bars indicate ± standard error of the mean (SEM). Sample sizes were *n* = 1007, 2383, and 395 valid observations for boreal, temperate, and tropical forests, respectively.

By comparing land surface temperature between forest habitats in PUIs and background PAs (“Methods”: Effect of urbanization on forest land surface temperature), we investigated the impact of urban pressure on the local thermal environment of protected forest habitats. We found that the land surface temperature (LST) of more than 70% of the forest habitats in PUIs is higher than in nearby background PAs (ΔLST = LST_PUI_-LST_PAB_ > 0). The daily mean forest land surface temperature in PUIs is 0.38 ±  0.01 °C higher than that in background PAs (Fig. 3a–c) If temperature differences between forest habitats in PUIs and background PAs were calculated with elevation difference within ±100 m, the daily mean ΔLST declined to 0.33 °C (Supplementary Table 2), demonstrating a slight influence of elevation differences between PUIs and background PAs.

We further quantified the relative contributions of the direct effect (e.g., canopy degradation caused by urbanization) (Supplementary Note 2, Supplementary Fig. 4 and Supplementary Fig. 5), indirect effect (e.g., urban heat spillovers), and residual component reflecting effects of topographic, edaphic, or other unaccounted factors to the thermal difference between PUIs and background PAs (Supplementary Note 3, Supplementary Fig. 6). We found that in the 0.38 ± 0.01 °C difference, about 0.18 °C is attributed to urbanization-induced direct forest canopy degradation, about 0.13 °C is attributed to indirect effects of urban heat spillover, and the remaining 0.07 °C is introduced by the differences in factors such as background topography and soil properties between PUIs and background PAs (Supplementary Note 3). Partial correlation analysis shows that the direct thermal effect of urbanization in PUIs is mainly contributed by population density (*r* = 0.31, *P* < *0.01*), forest edge density (*r* = 0.23, *P* < *0.01*), and impervious surface density (*r* = 0.21, *P* < *0.01*), which exerts pronounced pressure on canopy structures. The indirect effect of urbanization is dominated by impervious surface density (*r* = 0.39, *P* < *0.01*) followed by population density (*r* = 0.21, *P* < *0.01*), indicating their major role in shaping the strength of urban heat spillover (Supplementary Note 3, Supplementary Table 3).

The closer PUIs are to urban areas, the greater the warming effect of urbanization on forest habitats. The warming in forest habitats within 5 km buffer PUIs of urban areas in the boreal (0.17 ± 0.01 °C), temperate (0.43 ± 0.01 °C), and tropical (0.63 ± 0.05 °C) is much higher than that within 50 km buffer PUIs of urban areas in the boreal (0.02 ± 0.03 °C), temperate (0.11 ± 0.04 °C), and tropical (0.04 ± 0.05 °C) (Fig. 3d). This implies less impact of urbanization away from urban area, which is supported by the evidence that human footprint intensity declines with increasing distance from urban areas (Supplementary Table 1). Furthermore, the intensity and number of heat extremes, i.e., the 90th-percentile maximum land surface temperature, affecting forest habitats are much greater in PUIs than in background PAs, especially in the tropics, where annual mean intensity and number of heat extremes are 0.81 ± 0.06 °C and 0.36 ± 0.05 days higher than that in background PAs, respectively (Fig. 3e, Supplementary Fig. 7).

**Fig. 4:**
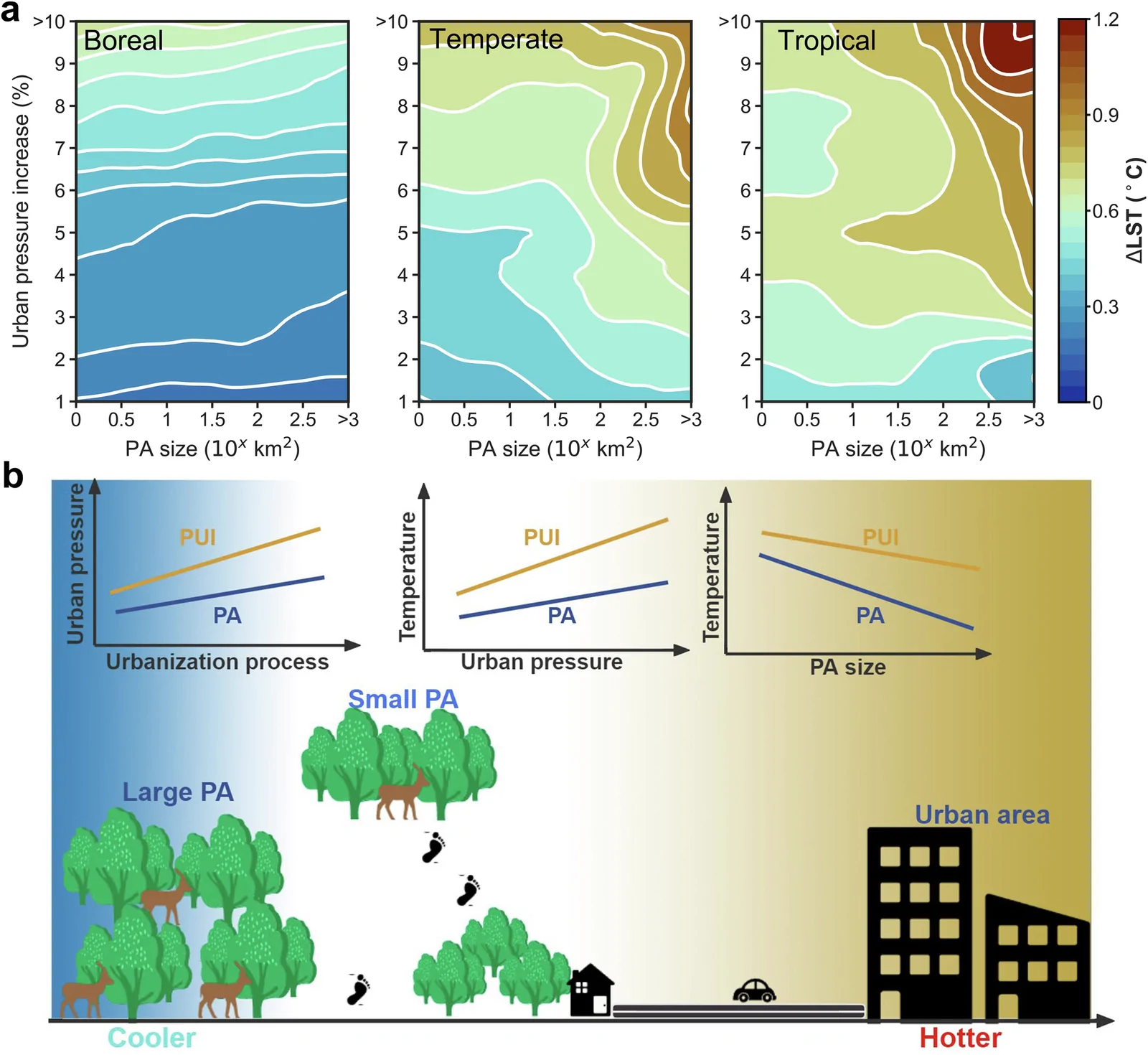
Warming effects on forest habitats in PAs influenced by PA size and urban pressure. **a** The relationships between warming effects on protected areas (PAs), urban pressure increases and PA sizes for protected boreal, temperate, and tropical forests, respectively. The white curves are the isotherms of the mean land surface temperature differences (ΔLST) increment. **b** Schematic diagram of hypothetical effects of thermal habitat degradation at the expanding protected area-urban interfaces (PUIs).

The forest PA size and urban pressure regulate the intensity of warming in forest habitats in PUIs (Fig. 4). For each 1% increase in urban pressure, the forest land surface temperature in PUIs increases by about 0.052 ± 0.002 °C (*r* = 0.92, *P* < 0.01), 0.045 ± 0.001 °C (*r* = 0.98, *P* < 0.01) and 0.019 ± 0.004 °C (*r* = 0.72, *P* < 0.01) in the boreal, temperate, and tropical regions, respectively. The alteration of the thermal environment in boreal forest habitats is not sensitive to PA size, yet highly sensitive to increasing urban pressure. By contrast, with increasing urban pressure, forest habitats in the temperate and tropical PUIs of larger PAs (area>1000 km^2^) tend to experience greater warming than background PAs. This suggests that in small PAs (area <10 km^2^), both PUIs and background PAs are often exposed to urban pressure, weakening original thermal buffering, whereas large PAs retain extensive interior areas distant from urban pressure and thus maintain stronger buffering capacity. The greatest difference in daily mean land surface temperature between PUIs and background PAs is 0.95 ± 0.55 °C in temperate forests and 1.22 ± 0.47 °C in tropical forests, occurring when the PA size exceeds 1000 km^2^ and urban pressure in PUIs increases by more than 10%. These results show that urbanization intensifies warming in protected forest habitats shaped by distance to urban areas and PA size, highlighting the need to incorporate thermal risk assessment into the urban planning and sustainable management of PAs.

## Discussion

Despite growing recognition of the effects of urbanization on natural landscapes, gaps remain in how urbanization exerts pressure on PAs, thereby reducing their capacity to buffer local climate. We provide a global assessment of the spatial extent and intensity of urban pressures on PAs, and the impact of urban pressures on the local thermal environments of protected forest habitats. The results offer observational evidence that, at the global level, between 1990 and 2018, increasing urban pressure on protected forest habitats is driven not only by urban expansion toward PAs, but also by rising built-up intensity in surrounding areas. Higher built-up intensity reflects rapid changes in urban land use, including increased building density, expanded transport networks, and more concentrated industrial and residential activities. Increased industrial facilities and transport infrastructures can transfer waste heat and pollutants into nearby PAs through atmospheric advection and diffusion^43^, while growing urban populations raise the frequency and intensity of human activities such as tourism and accommodation development, further increasing pressure on PAs^44^.

We show that urban pressure on forest habitats in PUIs was higher in smaller PAs, whereas forest habitats in PUIs of larger PAs exhibited greater temporal increases in urban pressure since 1990. These contrasting patterns arise because small PAs tend to be located closer to cities. Based on the 1990 baseline analysis (Supplementary Note 1), 24.65% (40.92%) of small PAs are within 5 km (10 km) of urban areas, compared to only 5.24% (8.50%) for large PAs. As a result, small PAs experience higher baseline urban pressure and have limited capacity for further urban expansion due to their small remaining non-PUI areas, making them more likely to be fully exposed to urban pressure. In contrast, large PAs are typically located farther from cities with extensive boundaries, leaving greater potential for urbanization in their surrounding areas and leading to greater temporal increases in urban pressure as urbanization progresses.

Observational evidence of warming in forest habitats of small PAs further verifies the urbanization-induced deterioration of small PAs. We find that, even in small PAs, land surface temperatures of forest habitats are lower than those of forest habitats in nearby PUIs, indicating that small PAs can still maintain a certain thermal buffering capacity compared to PUIs. However, the temperature difference between PUIs of small PAs and background PAs is lower than that between PUIs of large PAs and background PAs. This suggests that the impact of urbanization has already spilled over into the core areas of small PAs, leading to a gradual reduction in the differences of forest biophysical features between background PAs and nearby PUIs, thus reducing the thermal buffering capacity of small PAs. Nevertheless, safeguarding small PAs remains crucial, as forested habitats there continue to provide stronger thermal buffering than open lands, e.g., grasslands and croplands, against climate change impact on biodiversity.

It is commonly believed that large PAs maintain a natural and stable environment for biodiversity due to strict management practices and their original establishment in remote areas, away from human settlements^11,45^. However, with population growth and economic development, humans need more land for rapid urbanization. These remote large PAs are increasingly approached by expanding urban areas and tend to experience growing urban pressure^1,46^, especially in tropical regions, where rapid population growth and accelerating economic development are projected to drive substantial urban expansion in the coming decades^47^. Our results indicate that urban pressure can destabilize the thermal environment of forest habitats in large PAs (Fig. 4). Large PAs account for about 70% of the world’s protected forests and most of the large PAs are in tropical regions (Supplementary Fig. 1). The tropical forests exhibit stronger warming responses to degradation than temperate and boreal forests, because degradation in tropical forests leads to greater reductions in turbulent heat flux than in temperate and boreal forests, mainly due to larger declines in evapotranspiration and canopy roughness^28^.

Excessive warming in PUIs is attributed to direct effects of canopy degradation and indirect urban heat spillover. Canopy degradation in PUIs is reflected in the lower leaf area index, forest cover, and canopy height, which modifies surface energy budget and weakens local cooling capacity directly^48^. In addition, canopy degradation increases forest edge exposure and thus expands forest contact with urban heat indirectly^49^. Extensive impervious urban surfaces enhance solar radiation absorption and suppress evapotranspiration, leading to heat accumulation that warms near-surface air, and the excessive heat can be transported beyond urban boundaries through advection and diffusion^39^. As urban expands beyond original city margins, the transfer of accumulated heat into surrounding wildland areas is further amplified^38,41^. Furthermore, anthropogenic heat from transportation and infrastructures can propagate into distant wildland areas along transportation^50^.

In this study, the urban heat spillover is not directly quantified but inferred as the residual after subtracting the direct canopy effects from total urbanization-induced thermal effects. This residual represents a combination of physical processes regulating heat transport and redistribution, including urban morphology, wind patterns, and atmospheric circulation. The approach assumes a linear response of land surface temperature to urban pressure and treats canopy degradation and UHI spillover as additive effects without explicitly accounting for their interactions, which can introduce uncertainty into the attribution results. However, forest canopy and UHI effects interactions (e.g., UHI spillover influencing canopy structure) cannot be disentangled with current observations. In addition, some inferred UHI spillover effects may reflect indirect warming from industrial activities and infrastructure development outside city boundaries through canopy degradation and anthropogenic heat emissions, which are not separately resolved in our analysis. This gap highlights the need for nonlinear attribution models by integrating interpretable machine-learning approaches with numerical models, e.g., urban canopy models, while incorporating multiple anthropogenic processes to enable a more comprehensive attribution of urbanization effects on the protected forest thermal environment.

The satellite-derived land surface temperature used in this study is a critical proxy of forest-environment interactions; however, it much infers the radiative temperature of the upper canopies^51^. Land surface temperature may differ from 2 m air temperature, which is more commonly used in climate assessments^52^. Therefore, converting land surface temperature to corresponding air temperature responses is important but remains challenging due to complex land-atmosphere interactions and atmospheric circulation^53,54^. Moreover, observations of air temperature within canopies of different forest types remain limited, underscoring the need for field measurements and validations^32^. Nonetheless, our analysis based on satellite-derived land surface temperature can, to some extent, reflect urbanization-associated changes in the surface thermal conditions of forest habitats within PAs.

Urban-induced thermal pressure on protected forests varies across latitudes, indicating region-specific biodiversity impacts and management needs. Our results show that tropical forests, which host the highest levels of global biodiversity, face stronger urban-induced thermal pressure compared to forests in the mid- and high-latitude regions. Many tropical ectothermic species have evolved within narrow thermal ranges, often less than 2 °C between their realized temperatures and upper thermal limits^55,56^. For example, lowland tropical Anolis lizards and several tropical Drosophila species have already been subjected to temperatures approaching their critical thermal maxima, leaving warming tolerances of less than 2 °C^56,57^. The tropics have been warmed approximately 0.79 °C in the last five decades^58^. This additional local warming identified in tropical PUIs substantially narrows the thermal ranges of many species. As a result, biodiversity in tropical protected forests tends to face heightened risk under continued urban expansion. Given the rapid urbanization driven by population growth in most tropical countries, strengthening the management of existing PAs may offer a more feasible and effective path than restricting urban expansion. Enhancing forest integrity^59^, establishing buffer zones around PAs^60^, and further promoting urban green infrastructures^61^can mitigate UHI and strengthen the resilience of protected forests. Moreover, given the relatively stable trends of urban expansion in many mid- and high-latitude countries, an effective strategy for mitigating thermal pressure on protected forests involves integrating strict forest management within PAs with compact urban development^62^.

PUIs represent the portions of PAs that are, in principle, protected from direct development but remain increasingly susceptible to urbanization. Beyond the observed thermal degradation, more than 70% of forest habitats within PUIs exhibit lower connectivity (Supplementary Note 4, Supplementary Fig. 8a), and more than 64% of forest habitats within PUIs have lower mean species abundance than in background PAs (Supplementary Fig. 8b). When habitat fragmentation and heat stress occur simultaneously, they can reinforce each other and increase the risk of local extinction. However, these non-linear superposition effects are overlooked in the current studies. Although PUIs may face heightened risks of fragmentation, wildfire, and biodiversity loss under continued urban pressure^63^, they can still provide multiple benefits for biodiversity and society with their remaining natural attributes^61,64^. Compared with impervious surfaces, forests in PUIs retain greater evapotranspiration and stronger shading effects, thereby cooling habitats for urban and peri-urban wildlife against UHI^65^. We also found that within the 5 km urban buffer zone, protected forest habitats are 0.23 ± 0.01 °C cooler than unprotected forests, suggesting that forests within PUIs retain thermal buffering capacity despite urban warming. In addition, the temperature difference between unprotected forest habitats inside and outside urban buffer (5 km) zones is 0.43 ± 0.01 °C higher than the temperature difference between protected forest habitats in background PAs and PUIs. This result implies that without forest conservation, the thermal impact of urbanization is further amplified. Therefore, integrating PUI distribution into planning is essential for managing the trade-offs between building sustainable cities and safeguarding biodiversity.

Our study provides insights into how urbanization may constrain conservation efforts. From 1990 to 2018, a more than six-fold expansion of PUIs reflects accelerating urbanization toward PAs, thereby exacerbating thermal risk induced by urban pressure on PAs. Natural areas located less than 5 km from urban areas experience the strongest urban and thermal pressures, and are therefore not recommended as priority locations for future PAs. The urban-induced warming in protected forest habitats can extend up to 50 km from city boundaries, suggesting that conservation planning must account for urban influence at larger spatial scales. In addition, our results show that protected forests exposed to urban-induced thermal risk are also losing connectivity, which may limit the ability of species in these regions to move and track suitable climates. A practical solution is to establish ecological corridors connecting these isolated PAs, forming a larger conservation network to enhance the resilience of PAs against climate change^66,67^. We highlight the need for urbanization to fully integrate climate solutions, biodiversity conservation and socio-economic development needs.

## Methods

### Identification of PAs, urban areas, and their interfaces

The World Database on Protected Areas (WDPA) is a joint initiative between the United Nations Environment Program and the International Union for Conservation of Nature (IUCN), managed by the United Nations Environment Program World Conservation Monitoring Center (UNEP-WCMC)^2^. It is the most comprehensive global database of both marine and terrestrial PAs. The October 2018 release of the WDPA was used in this study to identify the PA boundary. We selected the PAs with areas larger than 1 km^2^ and IUCN category of I to VI (Supplementary Table 4), and further converted the selected PAs into a raster at 0.01° resolution to match the resolution of the remote sensing data used in subsequent analyses. We excluded the IUCN category of ‘not reported’, ‘not assigned’, or ‘not applicable’ due to the lack of adequate biodiversity assessment in these PAs^19^. Only the pixels with 100% coverage of PAs were identified as pure pixels of PAs. The PAs at 0.01° resolution were then overlaid with aggregated European Space Agency (ESA) Climate Change Initiative (CCI) land cover data of 1992 and 2018 at 0.01° to identify boreal, temperate and tropical forest pixels as forest habitats in PAs. The forest habitats represent the living environment of the species predominantly covered by forests, but may contain a very low fraction of non-forest because of challenges in identifying a pure forest pixel with satellite data^68^. Boreal forests are located within 50°N to 70°N, temperate forests within 25°N to 50°N and tropical forests within 25°S to 25°N (Supplementary Fig. 1a). Using forest cover data of 2018, the area of protected forests accounted for about 45% of the terrestrial PAs. Our analysis focused on protected forest pixels and does not include non-forest ecosystems within PAs. The findings are therefore most applicable to forest-dominated PAs.

The urban area data in 1990 and 2018 were extracted from the multitemporal global urban boundary (GUB) dataset^69^. The GUB data is derived from the global artificial impervious area (GAIA) dataset based on kernel density estimation, cellular automata, and geomorphological methods to reduce the uncertainty in identifying impervious surfaces in urban edge areas, and the GUB can stably capture the spatial changes of urban areas worldwide. The period 1990-2018 was selected because the GUB dataset used for the urban extent analysis is only available for this time period. We selected only urban areas with areas larger than 1 km^2^, and further converted the GUB data into a raster at 0.01° resolution. Only the pixels with 100% coverage of urban areas were identified as pure pixels of urban areas.

Protected area-urban interfaces (PUIs) were defined as the zone of transition between PAs and urban areas. Although urban areas rarely encroach directly on PAs, they can impact nearby PAs through thermal stress and human activities that spread beyond urban boundaries^35–37^. Therefore, we set buffer zones with various distances (5 km, 10 km, 25 km and 50 km) from the city boundary to represent different spillover ranges of thermal stress and human activities caused by urbanization. Typically, the 5 km distance from urban areas represents a threshold at which various human activities exert significant pressure on natural habitats^16^. When PAs are within about 10 km of urban areas, a distinct set of ecological and environmental challenges emerges, such as light pollution^1^. The 50 km is a reasonable distance estimate at which ecological interactions between a PA and its urban surroundings become nonnegligible^20^.

The PUI was mapped by intersecting the PA and the urban area buffer, which ensures that PUIs are within the transition zone between the PA and the urban area (Supplementary Fig. 1b-d). Forest habitats in PUIs were assumed to be affected by a combination of nature conservation and urbanization, while forest habitats in interior PAs were under conservation with minimal disturbances. If a PA is sufficiently small and located entirely within the buffer zone of an urban area, it will all be classified as PUIs, functioning as a transition zone between the urban area and the PAs that are not overlapped by the buffer zone of an urban area (Supplementary Fig. 1d). We further defined the portions of PAs excluding PUIs as background PAs.

### Quantification of urban pressure on forest habitats

We assumed that urban pressure caused by urbanization depends on the built-up intensity, i.e., the density of the built environment in urban areas, and the closer habitats are to the urban area of higher intensity, the greater urban pressure on habitats^70^. Therefore, urban pressure (UP) on forest habitats was calculated as a function of built-up intensity (*β*) and distance between forest habitats and urban areas (*D*) as

1 $${{UP}}_{{i}}=\frac{\mathop{\sum }_{{j}=1}^{{n}}\left({\beta }_{{j}}{{D}}_{{i},{j}}\right)}{\mathop{\sum }_{{j}=1}^{{n}}\left({\beta }_{max }{{D}}_{{i},{j}}\right)}$$

where UP_i_ is the urban pressure on a forest pixel *i*, which ranged from 0 (natural state with no urban pressure) to 1 (maximum urban pressure), and *β*_*j*_ is the built-up intensity of the pixels *j*, which ranged from 0 (fully vegetated surfaces) to 1 (*β*_*max*_, fully built-up surfaces). The built-up intensity was quantified using the annual maps of global artificial impervious area (GAIA)^71^. The GAIA was mapped annually at a 30 m resolution from 1985 to 2018, using the full archive of Landsat images, and the overall accuracy of the GAIA dataset is greater than 90%. The built-up intensity of each pixel at 0.01° resolution was calculated as the fraction of the GAIA impervious surface pixels within the 0.01° pixel. *D*_*i,j*_ is the weight assigned to the distance between pixel *i* and nearby pixel *j*, and *n* is the number of pixels around pixel *i* in the current urban spatial distribution (numbered *j* = 1, 2…n). *D*_*i,j*_ is calculated with a normalized Gaussian curve as

2 $${{D}}_{{ij}}={{e}}^{-\frac{{{x}}_{{ij}}^{2}}{2{{\sigma}}^{2}}}$$

where *x*_*ij*_is the euclidean distance between pixel *i* and nearby pixel *j*, and *σ* is the standard deviation of *x*_*ij*_. For a normalized Gaussian curve, about 99.99% of the data fall within the 4*σ* range. Therefore, we truncated the distribution of *D*_*i,j*_to zero at 4σ and the cumulative built-up intensity within 4*σ* of a forest pixel was calculated according to Eq. (1). A small value of *σ* corresponds to urban pressure at a local scale. We further tested different *σ* values and found that when *σ* was set at 2.5 km, the difference in urban pressure between PUI and PA was the most significant (Supplementary Table 5).

We assumed that the background climate for paired PUIs and background PAs was similar in each 0.25° window. We did not differentiate the management regimes of PAs. At this local to regional scale of 0.25° window, management intensity and effectiveness, which are shaped by local political and socio-economic conditions and shift over time^72^, are also assumed to be similar for PUIs and nearby background PAs. Disturbance caused by urbanization on forests in the background PAs was assumed to be lower. Forests in PUIs, however, were assumed to be exposed to more external disturbances caused by urbanization than protected forests in paired background PAs. Accordingly, across all paired 0.25° windows, forests in PUIs experience a higher urban pressure (3.20 ± 0.06%) than that in background PAs (0.64 ± 0.01%) in 2018. Forests in background PAs without urban pressure (UP = 0) account for less than 5% of forests in background PAs globally. Therefore, we quantify urban influence as a relative difference between PUIs and background PAs. Urban pressure on forest habitats in PUIs was then evaluated as the urban pressure difference (ΔUP) between PUIs and background PAs in 1990 and 2018 as

3 $$\Delta {{\rm{UP}}}={{{\rm{UP}}}}_{{{\rm{PUI}}}}-{{{\rm{UP}}}}_{{{\rm{PA}}}{{\rm{B}}}}$$

where UP_PUI_and UP_PAB_are the mean urban pressure in the same 0.25° window for forests in PUIs and background PAs, respectively (Supplementary Fig. 1d). Positive values thus reflect higher urban pressure on forest habitats in PUIs than in background PAs. Because of the lack of forest cover data for 1990, we used forest cover from ESA CCI land cover data in 1992, assuming the forest cover change between 1990 and 1992 was very low. To reduce systematic biases caused by elevation difference in the same window, the global 1 km resolution land surface digital elevation model (DEM) derived from the United States Geological Survey 30-arc sec SRTM30 gridded DEM data created from the NASA Shuttle Radar Topography Mission (SRTM) was used^73^. We constrained our analysis to windows where the elevation difference between background PAs and PUIs was within ±500 m.

To examine temporal changes in urban pressure, we calculated ΔUP changes between 1990 and 2018 (ΔUP_2018_-ΔUP_1990_). Because urban pressure is defined as a nonlinear function of urbanization intensity and distance to urban areas, we conducted an additional scenario-based analysis to examine the relative roles of urbanization intensity and distance to urban areas. We recalculated ΔUP_2018_ under two scenarios: (S1) retaining only pre-existing urban pixels (built-up intensity > 0 in 1990), (ΔUP_2018_-ΔUP_1990_) reflects changes associated with increasing urbanization intensity, and (S2) retaining only newly emerged urban pixels (built-up intensity = 0 in 1990 and > 0 in 2018), (ΔUP_2018_-ΔUP_1990_) reflects distance reduction due to urban expansion. In both cases, ΔUP1990 was calculated using the urban distribution and intensity of 1990. We showed changes in ΔUP (ΔUP_2018_-ΔUP_1990_) were 1.02% and 0.73%, for scenarios S1 and S2, respectively. This analysis indicates that changes associated with urbanization intensity cause a larger ΔUP change than distance reduction alone.

In addition, we analyzed the differences in human footprint intensity between PUIs and background PAs. Human footprint intensity is calculated as a weighted combination of eight human activity variables, including built environment, population density, nighttime lights, cropland, pasture, railways, roads, and navigable waterways, and has been updated to 2024^42,74^. We further calculated urban pressure and human footprint intensity in forest habitats within different urban buffer distances from 5 to 50 km (Supplementary Table 1). As forest habitats are farther away from urban areas, the urban pressure and human footprint intensity are reduced because of less residential facilities and human activities.

### Effect of urbanization on forest land surface temperature

We used 1 km MODIS 8-day Aqua land surface temperature data (MYD11A2) collected between 2014–2018 to analyze the forest land surface temperature change affected by biogeophysical effects^75^. In forests, the tree canopy buffers local microclimatic conditions, thereby affecting ecological processes^76^. Canopy temperature plays an important role in indicating forest microclimatic conditions. In the vegetated areas, especially areas covered by dense and tall trees, the land surface temperature observed by satellite corresponds to the temperature of the canopy surface rather than to the below-canopy temperature or the air temperature at 2 m above the ground surface^77^. The advantages of land surface temperatures obtained by remote sensing also lie in relatively high resolution and spatial continuity, which allows comparison of thermal conditions on different underlying surfaces. The overpass times of Aqua are around 13:30 and 1:30 local time each day and approximate the times of daily maximum and minimum temperatures, respectively. We only used land surface temperature with “mandatory QA flags” of 0 or 1 and “error flag” of 0 or 1, indicating that the land surface temperature was not affected by cloud, and the average land surface temperature error is lower than 2 K. Daily land surface temperature is calculated as an average of the daytime and nighttime land surface temperatures. We further averaged the daily land surface temperature from 8 days to a monthly scale and then calculated a 5-year (2014–2018) daily mean land surface temperature for further analysis. We used a space-for-time method to estimate the land surface temperature changes induced by the biogeophysical effect. In the same 0.25° window, we evaluated the land surface temperature difference (ΔLST) of forest between PUIs and nearby background PAs of the same forest type as

4 $$\Delta {\mathrm{L{ST}}={L{ST}}_{{PUI}}-{L{ST}}_{{PA}B}}$$

where LST_PUI_ and LST_PAB_ are the 5-year daily mean land surface temperature in the same 0.25° window for forest habitats in PUIs and background PAs, respectively. Positive values indicate warmer temperatures of forest habitats in PUIs compared to background PAs. We further used a space-for-time method to calculate the differences of intensity (mean extreme land surface temperature) and number of heat extremes (total days) between PUIs and background PAs in 5 years (2014–2018). An extreme heat day was determined as any day exceeding the 90^th^ percentile for maximum land surface temperature in the period of 2003–2018, which can be calculated by MODIS daytime land surface temperature data. All datasets used in the above analyses are summarized in Supplementary Table 6.

As urban areas are more likely to be distributed at a lower elevation than PAs due to topographical and accessibility constraints, we evaluated the impact of the elevation difference on the calculation of ΔLST. While our analyses suggest that the global and regional mean ΔLST bias caused by elevation is limited when the elevation difference between PUIs and PAs is below 500 m, we acknowledge that some residual bias may still exist (Supplementary Table 2). Sensitivity tests showed that reducing the elevation difference threshold from 500 m to 100 m led to a slight decrease in ΔLST estimates. On a global scale, ΔLST declined from 0.38 to 0.33 °C. Regionally, the change was relatively small in the boreal region (from 0.17 to 0.16 °C), but slightly greater in temperate (from 0.43 to 0.39 °C) and tropical (from 0.63 to 0.56 °C) regions. These results suggest that although elevation-induced biases are not negligible, they do not significantly affect our overall conclusions. To minimize such biases induced by elevation while maintaining sufficient sample coverage, we constrained our analysis in windows where the elevation difference between PAs and PUIs was within ±500 m.

### Reporting summary

Further information on research design is available in the Nature Portfolio Reporting Summary linked to this article.

## Supplementary information


Supplementary Information
Reporting Summary
Transparent Peer Review file


## Data Availability

The datasets supporting the findings of this study are obtained from publicly available sources. The World Database on Protected Areas (WDPA) is available at https://www.protectedplanet.net/en/thematic-areas/wdpa?tab=WDPA. Global Urban Boundaries (GUB) data is available at https://data-starcloud.pcl.ac.cn/iearthdata/14. Annual Global Artificial Impervious Area (GAIA) map is available at https://data-starcloud.pcl.ac.cn/iearthdata/13. Global Human Footprint data is available at https://doi.org/10.6084/m9.figshare.16571064. MODIS Aqua Land Surface Temperature (MYD11A2 Version 6) is available at https://doi.org/10.5067/MODIS/MYD11A2.006. MODIS Leaf Area Index (MOD15A2H Version 6) is available at https://doi.org/10.5067/MODIS/MOD15A2H.006. ESA CCI land cover data is available at https://www.esa-landcover-cci.org. ESA CCI aboveground biomass data is available at https://climate.esa.int/en/projects/biomass. Global Mean Species Abundance data is available at https://www.globio.info/globio-data-downloads.
