# Supplementary material for: Forests in protected areas worldwide face increased thermal pressure from urbanization
Source: Nat Commun. 2026 Jul 28;17:9171. doi: 10.1038/s41467-026-76226-8 (PMC13521939; doi:10.1038/s41467-026-76226-8)
Supplement: Supplementary file 1 [file 41467_2026_76226_MOESM1_ESM.pdf]

Supplementary Information for

Forests in protected areas worldwide face increased thermal pressure from urbanization

Anqi Huang[1], Xiyan Xu[2,3*], Gensuo Jia[2], Elise Belle[4], Pieter De Frenne[5], Yuyu Zhou[6,7]

[1]Key Laboratory for Climate Risk and Urban-Rural Smart Governance, School of Geography, Jiangsu Second Normal University, Nanjing 211200, China

[2]State Key Laboratory of Earth System Numerical Modeling and Application, Institute of Atmospheric Physics, Chinese Academy of Sciences, Beijing 100029, China

[3]Collaborative Innovation Center on Forecast and Evaluation of Meteorological Disasters (CIC-FEMD), Nanjing University of Information Science & Technology, Nanjing 210044, China

[4]United Nations Environment Programme World Conservation Monitoring Centre (UNEP-WCMC), Cambridge CB3 0DL, United Kingdom.

[5]Forest & Nature Lab, Department of Environment, Ghent University, Gontrode-Melle, Belgium

[6]Institute for Climate and Carbon Neutrality, The University of Hong Kong, Hong Kong SAR 999077, China

[7]Department of Geography, The University of Hong Kong, Hong Kong SAR 999077, China

*Correspondence to Xiyan Xu, xiyan.xu@tea.ac.cn

List of Supplementary Items

Supplementary Note 1 Urban expansion towards protected areas globally ………3

Supplementary Note 2 Forest habitat degradation in PUIs………………………..5

Supplementary Note 3 Attribution of urbanization-induced surface warming…..6

Supplementary Note 4 Reductions in forest connectivity and mean species abundance in PUIs……………………………..………………………………7

Supplementary Table 1 Differences in vegetation features and urban pressure indicators between forest habitats in PUIs and in background PAs with different urban buffer distances………………………………………..………………..11

Supplementary Table 2 Differences in land surface temperature between forest habitats in PUIs and in background PAs across different ranges of DEM differences…………………..……………………………….……………….. 12

Supplementary Table 3 Partial correlation of drivers for direct and indirect thermal effects…………………..…………………………...………………..13

Supplementary Table 4 Brief introductions to the IUCN protected area categories I to VI…………………..…………………………...…………………..……..14

Supplementary Table 5 Mean differences in urban pressure (ΔUP) between in PUIs and in background PAs with different σ value………………………………15

Supplementary Table 6 Overview of datasets used in this study…………………16

Supplementary Figure 1 Global urban areas and protected forests………...……17

Supplementary Figure 2 Global urban area expansion towards PAs during 1990-2018…………………...….…………………………………………………………18

Supplementary Figure 3 Increased human footprint intensity on protected forests …………………………………………………………………...……19

Supplementary Figure 4 Leaf area index difference between protected forests in PUIs and in background PAs during in 2018…………………………………..20

Supplementary Figure 5 Aboveground carbon deficit between protected forests in PUIs and in background PAs in 2018……………..………………………………..21

Supplementary Figure 6 Attribution of urbanization-induced surface warming of forest in PUIs……………………………………………………………………...22

Supplementary Figure 7 Influence of urbanization on heat extremes of forest habitats in PUIs compared to background PAs……………….…………………23

Supplementary Figure 8 Reductions in forest connectivity and mean species abundance in PUIs………………………………………………………………..24

Supplemental References………………………………………………………...25

Supplementary Note 1 Urban expansion towards protected areas globally

We included 18,814 PAs established before 1990 with areas larger than 1 km^2 and with an assigned IUCN category I to VI. We extracted the geodesic distance between the urban boundary and the protected area (PA) boundary. The geodesic distance is the shortest distance between two points considering the curvature of the Earth. We further quantified global urban area expansion towards PAs over the 1990-2018 time period with an expansion distance and expansion rate as

$$\text{Expansion distance} = MiD_{1990} - MiD_{2018} \quad (1)$$

$$\text{Expansion rate} = \frac{MiD_{1990} - MiD_{2018}}{MiD_{1990}} \quad (2)$$

where MiD_{1990} and MiD_{2018} are the minimum distances from PA boundaries to the closest urban boundaries in 1990 and 2018, respectively. We chose PAs established before 1990 for calculating distances from urban areas, thus preventing confusion regarding whether changes in distance are due to the establishment of new PAs surrounding urban areas or the expansion of urbanization towards PAs.

Between 1990 and 2018, more than 95% of PAs have experienced approaching urban expansion, and the global mean expansion distance of urban areas towards PAs during this period is 12.82±0.23km (means ± standard error) (Supplementary Figure 2a and b). Urbanization has greatly shortened the distance between urban areas and PAs in tropical regions (22.91±1.08km) compared to boreal (13.75±0.45km) and temperate (5.53±0.15km) regions. This is because of shifting baselines as urban areas originally maintained a substantially long distance from PAs in the tropics (183.83±11.67km in 1990), while urban areas in temperate regions were developed very close to PAs already in 1990 (26.85±1.29km in 1990). This spatial arrangement results in lower expansion rate of urban areas towards PAs in the tropics (28.31±0.78%) than in boreal (35.59±0.45%) and temperate regions (35.55±0.39%).

The expansion distance of urban areas towards PAs increases with PA size in boreal (r=0.91, $p<0.01$) and temperate (r=0.86, $p<0.01$) regions indicating that urbanization has greatly shortened the distance between urban and remote large PAs in

these regions (Supplementary Figure 2c). In the tropics, however, the expansion distance of urban areas towards PAs is less correlated to PA size because urban areas are basically still far from PAs. Despite urbanization greatly shortened distance between urban and large PAs, the expansion rate of urban areas towards PAs remains relatively low largely due to the originally long distances between them. We showed 24.65% (40.92%) of small PAs are within 5 km (10 km) of urban areas, compared to only 5.24% (8.50%) for large PAs in 1990. The expansion rate of urban areas towards PAs decreases significantly with increasing PA size in boreal (r=0.90, $p<0.01$), temperate (r=0.89, $p<0.01$) and tropical (r=0.92, $p<0.01$) regions (Supplementary Figure 2d).

Supplementary Note 2 Forest habitat degradation in PUIs

Forest habitat degradation in protected area-urban interfaces (PUIs) was quantified by changes in leaf area index (LAI) and aboveground biomass carbon (AGC) of forests. We used aggregated ESA CCI global aboveground biomass data[1] and MODIS LAI data (MCD15A2H)[2] at 0.01° resolution in 2018 to analyze the forest carbon density difference and leaf area difference between PUIs and background PAs in each 0.25° window. We constrained our analysis in windows where the elevation difference between background PAs and PUIs was within ±500 m to avoid biases induced by elevation.

We found that urban pressure causes forest habitat degradation in PUIs inferred by the reduced leaf area index of 0.11± 0.01 $m^2 m^{-2}$ (Supplementary Figure 4) and reduced forest carbon density of 2.22± 0.25 MgC ha^{-1} in 2018 compared to forest habitats in background PAs (Supplementary Figure 5). Forest LAI loss of PUIs compared to background PAs in tropical forests (-0.13±0.03 $m^2 m^{-2}$) is higher than that in temperate forests (-0.12± 0.01 $m^2 m^{-2}$) and in boreal forests (-0.08± 0.01 $m^2 m^{-2}$). Forest AGC loss of PUIs compared to PAs in tropical forests (-4.69±0.51 $MgCha^{-1}$) is about twice as much as in temperate forests (-2.29±0.45 $MgCha^{-1}$) and four times as much as in boreal forests (-1.14±0.42 $MgCha^{-1}$). The degradation decreases as the distance between protected forest habitats and urban areas increases (Supplementary Table 1).

Supplementary Note 3 Attribution of urbanization-induced surface warming

To isolate the contribution of urbanization to warming of forest, we assume that, under the same background climate, differences in forest LST between PUIs and background PAs are primarily associated with urbanization-driven human disturbance. To assess the extent to which urbanization contributes to land surface temperature increases in PUIs, we estimated the temperature change induced by urban pressure as the product of its sensitivity and magnitude:

$$\Delta\text{LST}=\left(\frac{\partial\text{LST}}{\partial\text{UP}}\right)_{\text{PA}}\Delta\text{UP}+\varepsilon \quad (3)$$

where $\left(\frac{\partial\text{LST}}{\partial\text{UP}}\right)_{\text{PA}}$ represents the overall sensitivity of land surface temperature to urban pressure in PAs. This sensitivity was estimated using the Theil–Sen slope in each 0.25° grid cell. ΔUP is the difference in urban pressure between PUIs and background PAs in each 0.25° grid cells, reflecting the extent of forest habitat degradation associated with urban pressure. ε is the residual term, indicating residual biases introduced by factors such as topography, soil properties, and remote sensing uncertainties.

$\left(\frac{\partial\text{LST}}{\partial\text{UP}}\right)_{\text{PA}}\Delta\text{UP}$ provides an estimate of land surface temperature change attributable specifically to urban pressure. To better interpret the underlying processes, we further decomposed this term into two components: (i) direct effects driven by forest canopy degradation ($\Delta\text{LST}_{\text{direct}}$), and (ii) indirect effects arising from urban heat island (UHI) spillover and other spillover effects of human activities ($\Delta\text{LST}_{\text{indirect}}$):

$$\Delta\text{LST}_{\text{UP}}=\left(\frac{\partial\text{LST}}{\partial\text{UP}}\right)_{\text{PA}}\Delta\text{UP}=\Delta\text{LST}_{\text{direct}}+\Delta\text{LST}_{\text{indirect}} \quad (4)$$

This decomposition assumes that urban pressure influences LST through both local biophysical changes in forest canopy structure and non-local UHI spillover from surrounding urban areas, which can be approximated as additive contributions at the grid scale.

The direct effects of urban pressure on land surface temperature ($\Delta\text{LST}_{\text{direct}}$) from structural canopy degradation, including reductions in leaf area index (LAI) (horizontal canopy loss), canopy height (CH) (vertical structural decline), and forest cover (FC) (overall extent of forest loss). These changes alter surface energy budget by reducing turbulent heat flux and exposing darker surfaces with lower albedo, thereby

contributing directly to land surface warming in forests. To quantify this, we estimated $\Delta\mathrm{LST}_{\mathrm{direct}}$ by aggregating the warming contributions associated with each canopy variable:

$$\Delta\mathrm{LST}_{\mathrm{direct}}=\left(\frac{\partial\mathrm{LST}}{\partial\mathrm{LAI}}\frac{\partial\mathrm{LAI}}{\partial\mathrm{UP}}+\frac{\partial\mathrm{LST}}{\partial\mathrm{CH}}\frac{\partial\mathrm{CH}}{\partial\mathrm{UP}}+\frac{\partial\mathrm{LST}}{\partial\mathrm{FC}}\frac{\partial\mathrm{FC}}{\partial\mathrm{UP}}\right)_{\mathrm{PA}}\Delta\mathrm{UP} \quad (5)$$

Where $\left(\frac{\partial()}{\partial\mathrm{UP}}\right)_{\mathrm{PA}}$ represent the sensitivities of leaf area index, canopy height and forest cover to urban pressure in PAs. We estimated these sensitivities using the Theil–Sen slope in each 0.25° grid cell, based on MODIS LAI data (MCD15A2H) at 0.01° resolution from 2018 and forest cover and height data from the GLAD Global Land Cover and Land Use Change dataset from 2020. $\left(\frac{\partial\mathrm{LST}}{\partial()}\right)_{\mathrm{PA}}$ represent the sensitivities of land surface temperature to leaf area index, canopy height, and forest cover, respectively, in PAs. These sensitivities were estimated by fitting multiple linear regression models of land surface temperature against leaf area index, canopy height, and forest cover across protected forest pixels within each 0.25° grid cell, thereby isolating their independent associations with surface temperature.

The indirect effects of urban pressure include UHI spillover as well as other spillover effects of human activities, such as increased heat emissions, altered irrigation practices, or reduced wind flow from nearby built-up areas. These processes can alter the thermal environment of forest habitats without involving direct canopy degradation. Due to the lack of a direct attribution method for indirect effects, we estimated $\Delta\mathrm{LST}_{\mathrm{indirect}}$ as the residual difference between the total temperature change attributable to urban pressure and the portion explained by direct canopy degradation.

At the global scale, the mean observed land surface temperature difference between PUIs and background PAs was 0.38 ± 0.01 °C. Of this, 0.31 ± 0.01 °C (contribution rate = 81.6%) was attributed to urban pressure (Supplementary Figure 6a), in which 0.18 °C was linked to direct effects of forest canopy degradation (Supplementary Figure 6b), with contributions by loss in leaf area index (0.07 ± 0.01 °C, 18.4%), canopy height (0.05 ± 0.01 °C,13.2%), and forest cover (0.06 ± 0.01 °C,15.8%). The remaining 0.13 ± 0.01 °C (34.2%) was attributed to indirect effects (Supplementary Figure 6c), including potential UHI spillover and other non-structural human activity spillovers.

These findings underscore the dual contribution of direct forest degradation and indirect urban influences in shaping the land surface temperature of forests in PUIs.

We further chose six explanatory variables including differences in population density (ΔPOP), road density (ΔRD), agricultural density (ΔAG), impervious surface density (ΔIS), edge density (ΔED) between forests in PUIs and background PAs within each 0.25° grid cell. The population density, road density, agricultural density, impervious surface density are from LandScan Global Gridded Population Dataset[3], Global Roads Inventory Project Dataset[4], Global Artificial Impervious Area Dataset[5] and GLAD Global Land Cover and Land Use Change Dataset, respectively, and are aggregated to a 0.01° resolution[6]. Forest edge density was defined as the ratio of total edge length to window area within each 0.01° grid cell[7], and was calculated using the GLAD Global Land Cover and Land Use Change dataset. We used Ordinary Least Squares (OLS) model to calculate the partial correlation coefficients of these explanatory variables with direct ($\Delta \mathrm{LST}_{\mathrm{direct}}$) and indirect ($\Delta \mathrm{LST}_{\mathrm{indirect}}$) land surface temperature difference. To ensure the robustness of the results, we ran the OLS models by randomly subsampling 30% samples with replacement out of the entire data 500 times and calculated the mean partial correlation coefficient for each explanatory variable across all model runs. Compared with forests in background PAs (Supplementary Table 3), forests in PUIs show direct warming associated with population density, forest edge density, and impervious surface density, suggesting more pronounced canopy degradation under these pressures. Indirect warming is dominated by impervious surface density and population density, indicating that these factors play a major role in shaping the strength of urban heat spillover.

Supplementary Note 4 Reductions in forest connectivity and mean species abundance in PUIs

Urbanization not only elevates land surface temperatures but also alters landscape structure and biodiversity-related attributes of protected forests. We further quantified the impacts of urbanization on forest connectivity and mean species abundance (MSA) in PAs.

Forest connectivity was calculated based on the ESA CCI forest cover dataset aggregated to a spatial resolution of 0.01° for the year 2018. Connectivity was quantified following the method proposed by Grantham et al.[8], which estimates the probability of functional connections among forest patches. We applied three dispersal parameters (σ = 5, 20, and 50), representing short-, medium-, and long-distance dispersal processes, respectively. The final forest connectivity value was derived as the mean of the connectivity estimates across these three dispersal scenarios.

Global MSA data at 300 m spatial resolution were obtained from the Global Biodiversity Model[9]. MSA ranges from 0 to 1, where a value of 1 indicates a fully intact species assemblage and a value of 0 indicates the complete local extirpation of the original species assemblage. Due to the unavailability of MSA data for 2018, we used the temporally closest dataset from 2015, which includes terrestrial plants and warm-blooded vertebrates. The MSA data were subsequently aggregated to a spatial resolution of 0.01°.

In the same 0.25° window, we evaluated the differences in connectivity (ΔCON) and MSA (ΔMSA) of forest habitats between in PUIs and in background PAs. The observed differences are assumed to reflect the combined influence of urbanization and associated human activities (e.g., infrastructure, agriculture, and industrial activities). We constrained our analysis to windows where the elevation difference between PAs and PUIs was within ±500 m. We show that more than 70% of forest habitats within PUIs exhibit lower connectivity (ΔCON < 0) compared to background PAs, with an average ΔCON of -6.31±0.23% (Supplementary Figure 8a). Similarly, more than 64% of forest habitats within PUIs have lower mean species abundance (ΔMSA<0) than those in background PAs, with an average ΔMSA of -2.41±0.18% (Supplementary Figure 8b). Furthermore, we find that over 55% of protected forests are simultaneously affected by urbanization, experiencing rising surface temperatures (ΔLST>0), decreased connectivity (ΔCON< 0), and declining MSA (ΔMSA<0). These results

indicate that urbanization is making protected forests increasingly warmer and more isolated, thereby contributing to reductions in local species abundance. To assess the influence of edge effects, we excluded forest pixels within 1 km of PA boundaries and found that the mean values of ΔCON (−6.12 ± 0.15%) and ΔMSA (−2.24 ± 0.11%) changed only slightly, indicating a limited impact of edge effects on our results.

Supplementary Table 1 Differences in vegetation features and urban pressure indicators between forest habitats in PUIs and in background PAs with different urban buffer distances

Buffer distance	5km	10km	25km	50km
ΔLAI (m^2m^{-2})	-0.11	-0.08	-0.05	-0.02
ΔAGC ($MgCha^{-1}$)	-2.22	-2.09	-1.05	-0.42
ΔUP (%)	2.55	2.01	1.05	0.18
ΔHFP (%)	8.43	5.86	2.54	0.89

Supplementary Table 2 Differences in land surface temperature between forest habitats in PUIs and in background PAs across different ranges of DEM differences

ΔDEM range	500m	250m	100m
Global ΔLST (℃)	0.38	0.36	0.33
Boreal ΔLST (℃)	0.17	0.17	0.16
Temperate ΔLST (℃)	0.43	0.42	0.39
Tropical ΔLST (℃)	0.63	0.60	0.56

Supplementary Table 3 Partial correlation of drivers for direct and indirect thermal effects. '*' and '**' indicate $P < 0.05$ and $P < 0.01$, respectively, and ns indicates not significant. P values were derived from two-sided Pearson correlation tests.ΔPOP, ΔRD, ΔAG, ΔIS, and ΔED denote differences between forest in PUIs and background PAs in population density, road density, agricultural intensity, impervious surface density, and edge density, respectively.

Drivers	Direct effect (r)	Indirect effect (r)
ΔPOP	0.311 **	0.211 **
ΔIS	0.211 **	0.391 **
ΔED	0.227 **	0.187 **
ΔAG	0.115 *	0.081 ns
ΔRD	0.108 ns	0.068 ns

Supplementary Table 4 Brief introductions to the IUCN protected area categories I to VI

Category	Name	Description
Ia	Strict Nature Reserve	Areas strictly protected for conservation of biodiversity and are also possibly for geological or geomorphological features. Human visitation, use, and impacts are typically very limited.
Ib	Wilderness Area	Areas large unmodified or slightly modified areas, retaining their natural character and influence without permanent or significant human habitation. They are also protected and managed to preserve their natural condition.
II	National Park	Areas set aside for ecosystem protection and recreation. Human activities and impacts are limited.
III	Natural Monument or Feature	Areas set aside to protect a specific natural monument, which can be a landform, sea mount, submarine cavern, geological feature, or even a living feature such as an ancient grove.
IV	Habitat/Species Management Area	Areas set to protect specific species or habitats, and management reflects this priority. Often these areas are developed through active interventions.
V	Protected Landscape/Seascape	Areas where the interaction of people and nature over time has produced an area of distinct character with significant ecological, biological, cultural, and scenic value.
VI	Protected Area with Sustainable Use of Natural Resources	Areas set to protect ecosystems and habitats, while permitting a range of sustainable, low-level, non-industrial use of natural resources.

Supplementary Table 5 Mean differences in urban pressure (ΔUP) between in PUIs and in background PAs with different σ value.

σ (km)	1	2	2.5	3	5	7.5	10
ΔUP (%)	2.02	2.37	2.55	2.45	2.15	1.41	1.01
Standard Error (%)	0.07	0.06	0.06	0.06	0.06	0.05	0.05

Supplementary Table 6 Overview of datasets used in this study

Dataset	**Source**	**Spatial resolution**	**Temporal coverage**
World Database on Protected Areas	UNEP-WCMC / IUCN	Vector	2018
ESA CCI Land Cover	ESA	300m	1992, 2018
Global Urban Boundary	iEarth DataHub	Vector	1990-2018
Global Artificial Impervious Area	iEarth DataHub	30 m	1990-2018
Human Footprint Intensity	Mu et al.[10]	1 km	2000-2024
SRTM30 DEM	USGS / NASA	1 km	Static
MODIS Aqua LST (MYD11A2)	USGS / NASA	1 km	2003-2018
MODIS LAI (MOD15A2H)	USGS / NASA	500 m	2001-2018
ESA CCI Biomass	ESA	100 m	2020
GLAD Global LCLUC	University of Maryland	30 m	2020
Global Mean Species Abundance	GLOBIO	300m	2015

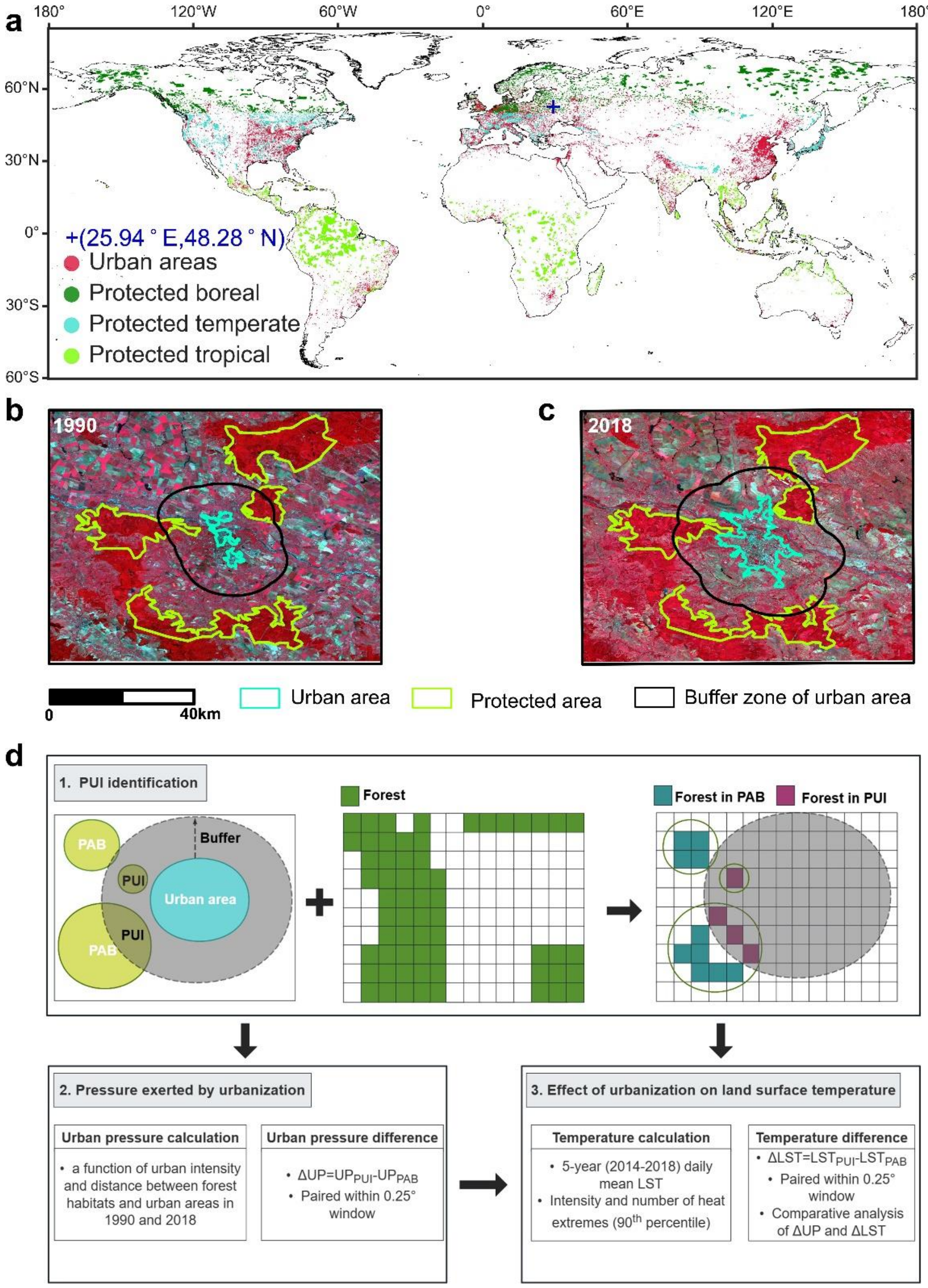

Supplementary Figure 1 Global urban areas and protected forests. (a) Spatial distribution of global urban areas and protected boreal, temperate and tropical forests in 2018, (b) example of Landsat-5 false-color composite of near infrared, red, and green bands at 30-m resolution for urban areas, protected areas and buffer zone of urban area in Ukraine (25.94°E，48.28°N) on August, 1990, (c) example of Landsat-8 false-color composite for the same area, on August, 2018, and (d) diagram illustrating the main research steps to identify the protected area-urban interface (PUI) and their relative position with background protected area (PAB), and calculate urban pressure (UP) and effect of urbanization on land surface temperature (LST).

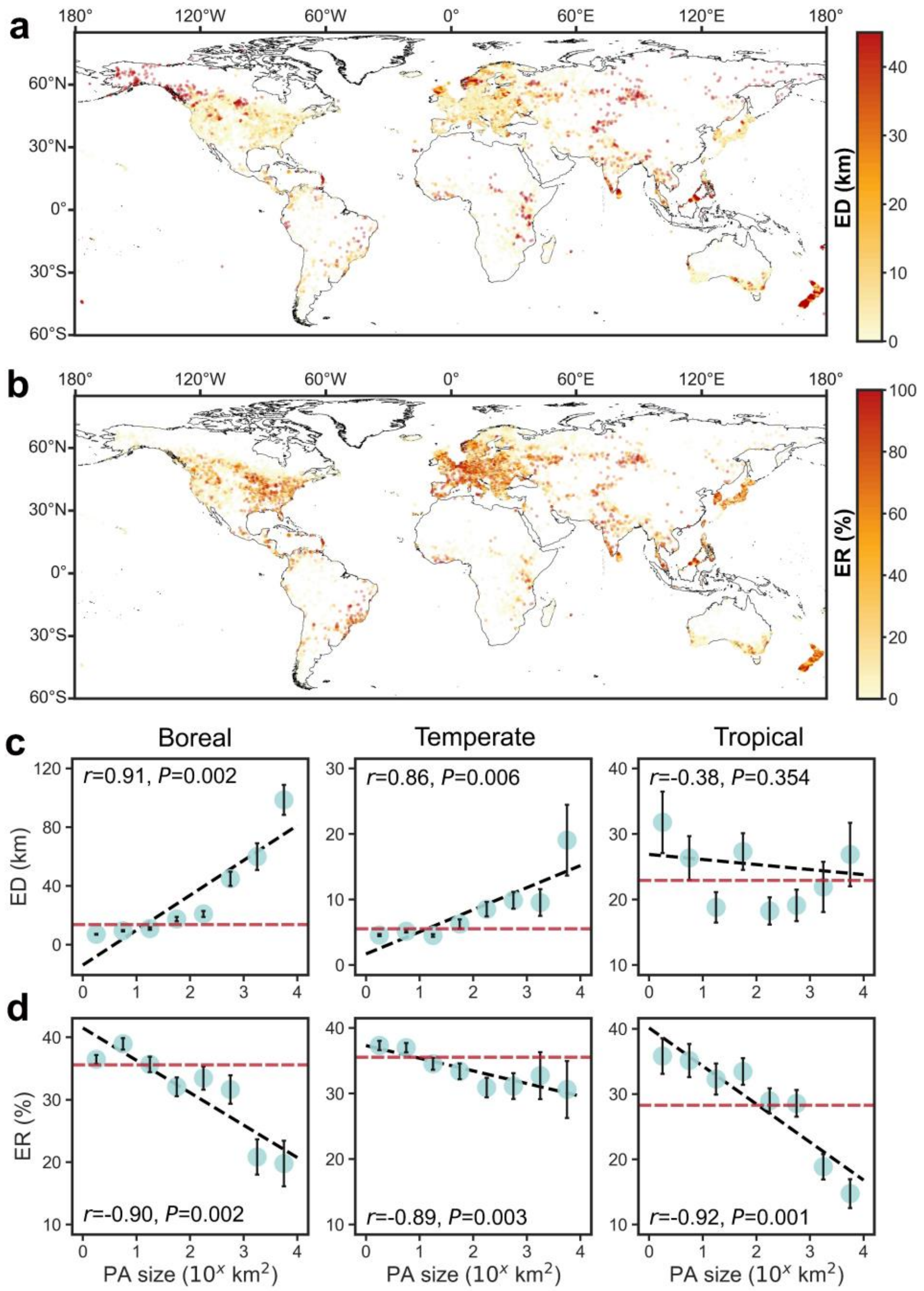

Supplementary Figure 2 Global urban area expansion towards PAs during 1990-2018. (a) The expansion distance (ED) of urban areas towards PAs, (b) expansion rate (ER) of urban areas towards PAs, (c) expansion distance of urban areas towards protected boreal, temperate, and tropical forests with different PA sizes, and (d) expansion rate of urban areas towards protected boreal, temperate, and tropical forests with different PA sizes. In (c) and (d), points show mean values and error bars indicate ± standard error of the mean (SEM). Dashed red lines indicate the overall mean across all observations, and dashed black lines show linear regression trends. Correlations were assessed using two-sided Pearson correlation tests. The displayed *r* and *P* values represent the Pearson correlation coefficient and exact *P* value, respectively. Sample sizes were n = 6149, 7350, and 5315 protected areas for boreal, temperate, and tropical forests, respectively.

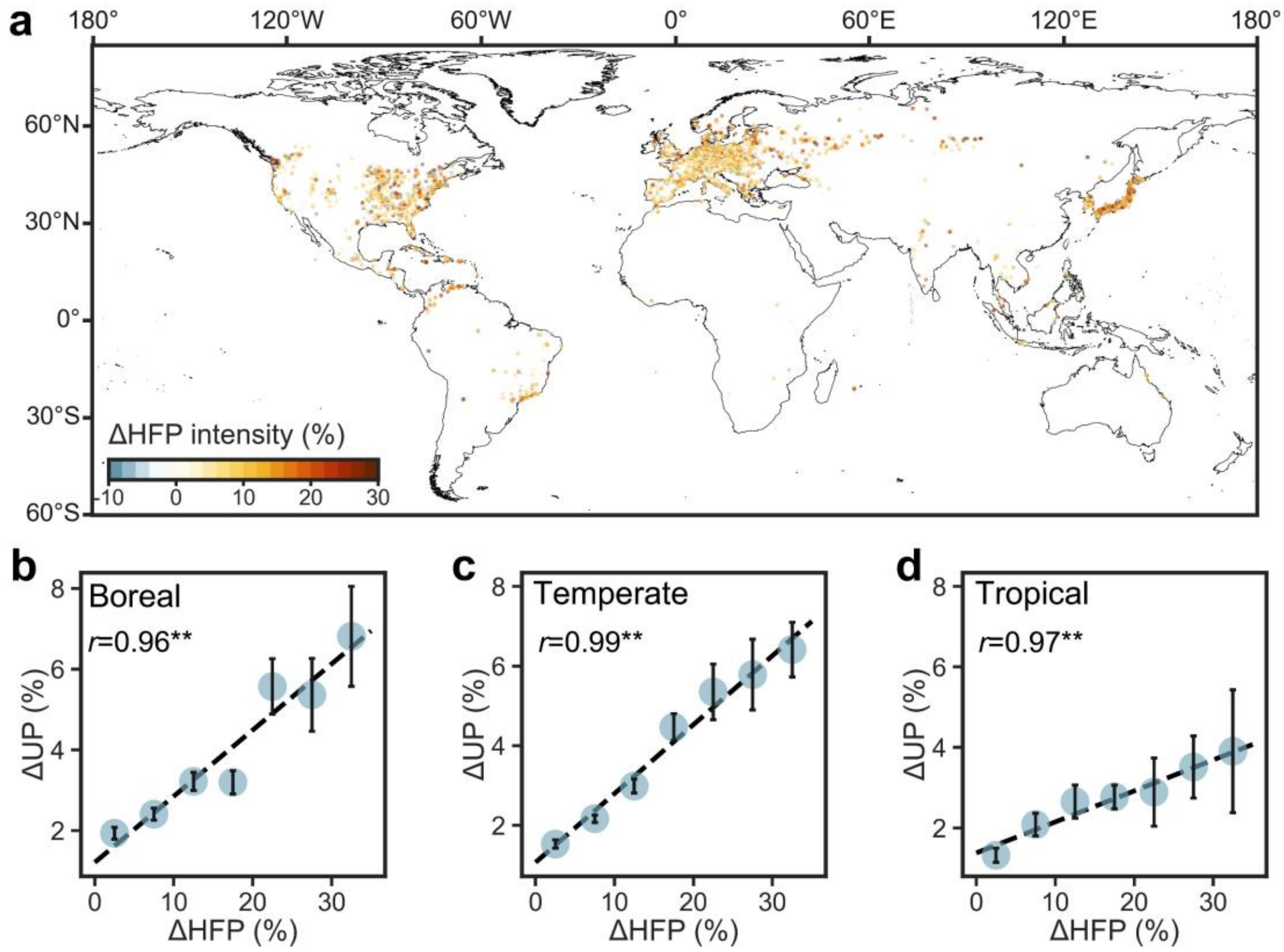

Supplementary Figure 3 Increased human footprint intensity on protected forests. (a) The human footprint intensity difference (ΔHFP) between forest habitats in PUIs and background PAs in 2018, and (b-d) correlation between ΔHFP and urban pressure difference (ΔUP) in boreal, temperate, and tropical protected forests. In (b–d), points show mean values and error bars indicate ± Standard Error of the Mean (SEM). The dashed black lines indicate linear regression trends. Correlations were assessed using two-sided Pearson correlation tests, and the symbol ** represents P value<0.01. Sample sizes were n = 1007, 2383, and 395 valid observations for boreal, temperate, and tropical forests, respectively.

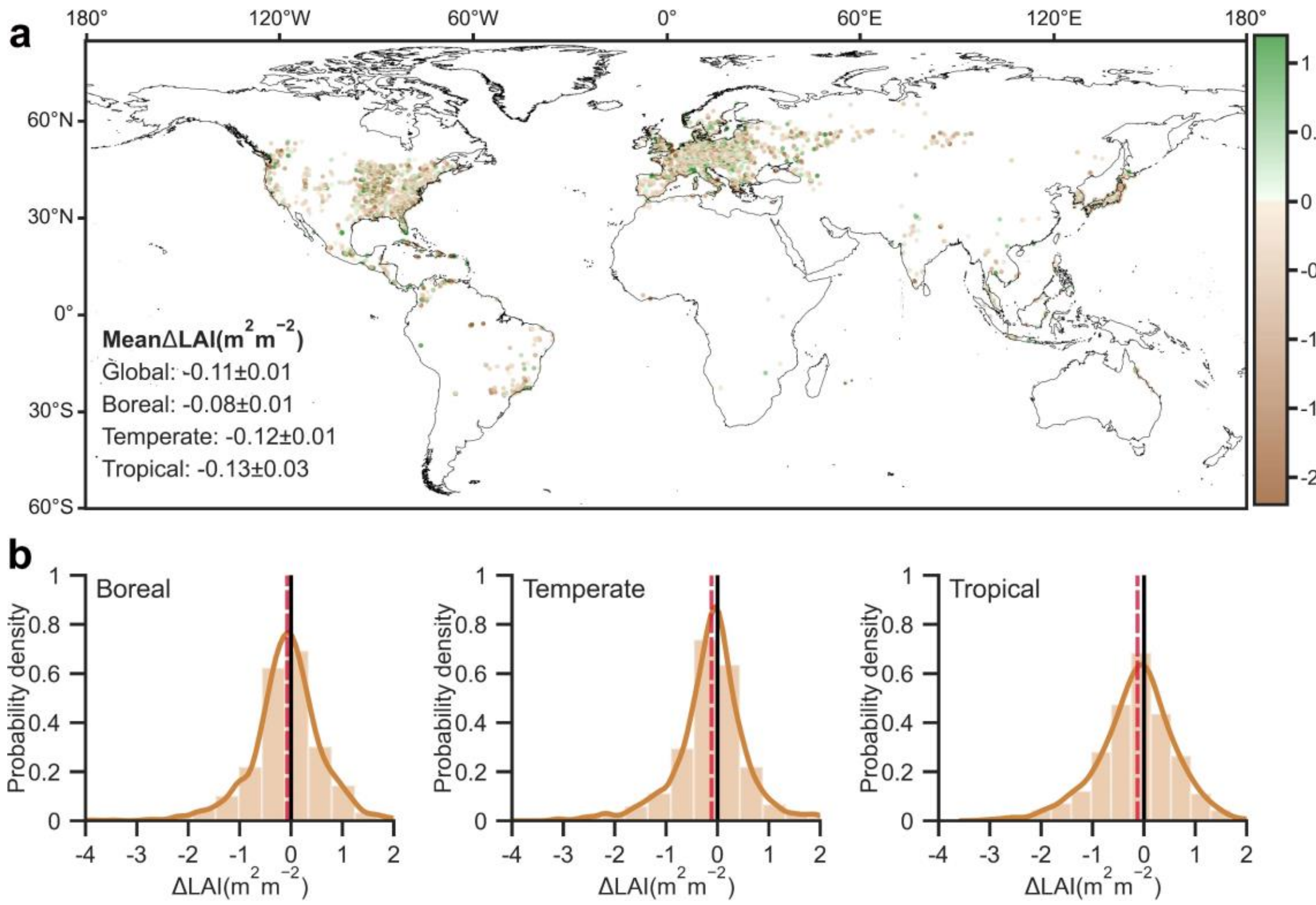

Supplementary Figure 4 Leaf area index difference between protected forests in PUIs and in background PAs during in 2018. (a) Spatial distribution of leaf area index deficit (ΔLAI) between protected forests in PUIs and in background PAs, ,with values representing mean ± standard error of the mean (SEM), and (b) kernel density distributions of ΔLAI for protected boreal, temperate, and tropical forests, respectively. The red dashed lines in (b) denote the mean values of ΔLAI. The red dashed lines are biased to the left of the black solid lines at ΔLAI=0, indicating the dominant negative impact of urbanization on leaf area index.

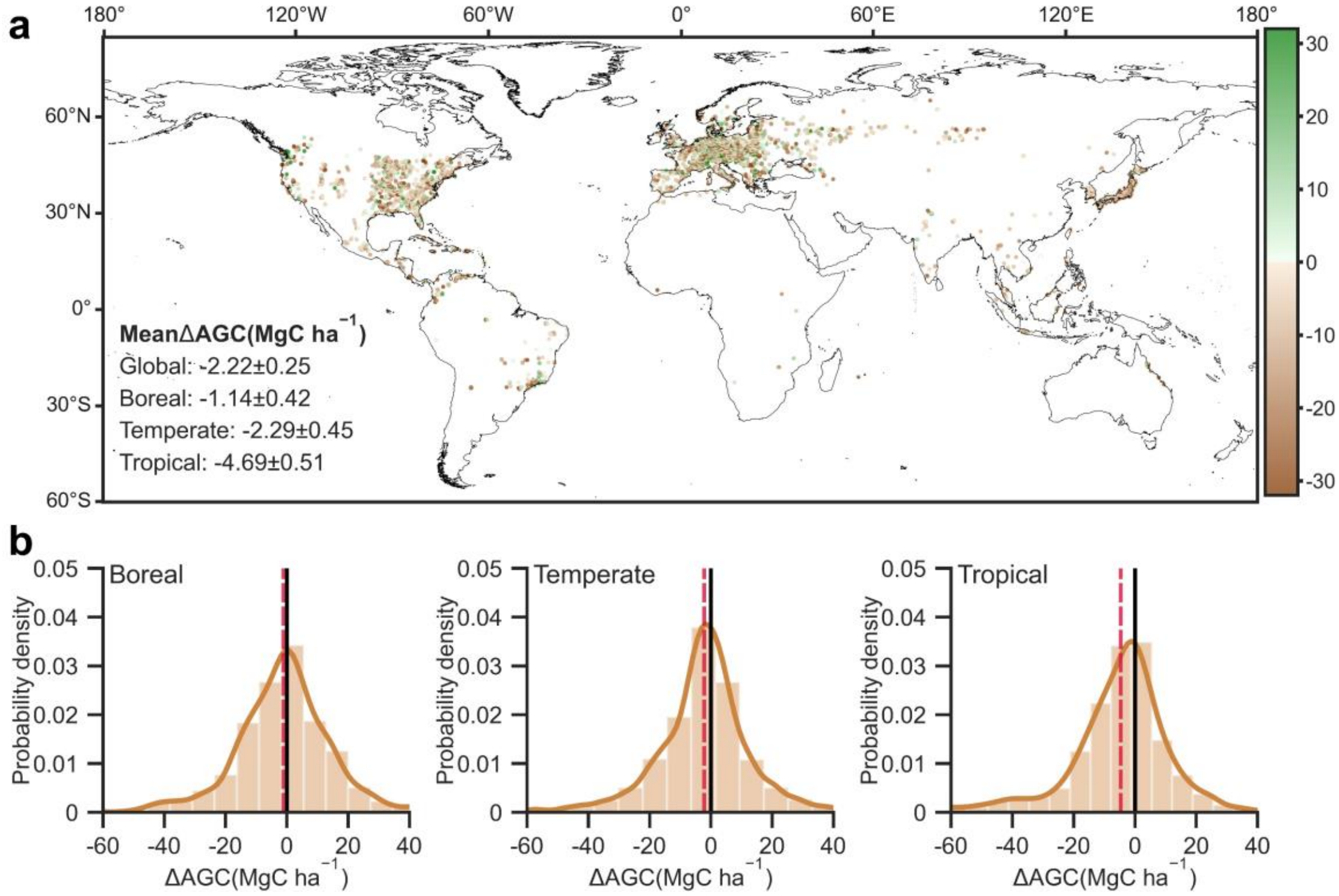

Supplementary Figure 5 Aboveground carbon deficit between protected forests in PUIs and in background PAs in 2018. (a) Spatial distribution of aboveground carbon deficit (ΔAGC) between protected forests in PUIs and in background PAs, with values representing mean ± standard error of the mean (SEM), and (b) Kernel density distributions of ΔAGC for protected boreal, temperate, and tropical forests, respectively. The red dashed lines in (b) denote the mean values of ΔAGC. The red dashed lines are biased to the left of the solid black lines at ΔAGC=0, indicating the dominant negative impact of urbanization on aboveground carbon.

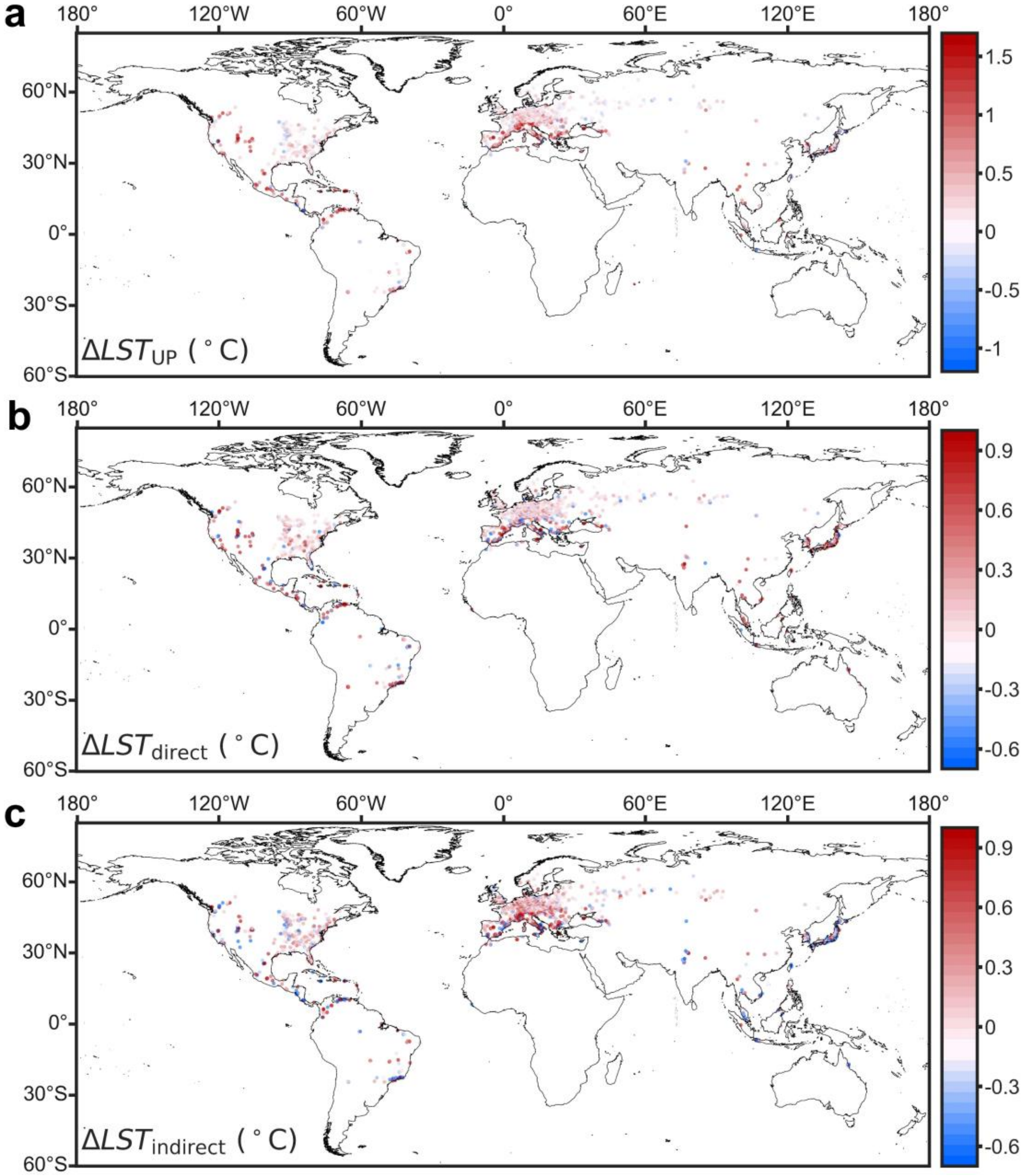

Supplementary Figure 6 Attribution of urbanization-induced surface warming of forest in PUIs. (a) Total surface warming attributed to urbanization pressure (ΔLST_{up}), (b) direct warming effects (ΔLST_{direct}) driven by forest canopy degradation, including reductions in leaf area index, canopy height, and forest cover, and (c) indirect warming effects ($\Delta LST_{indirect}$), estimated as residuals and capturing spillover influences of urban heat and other non-structural human activities.

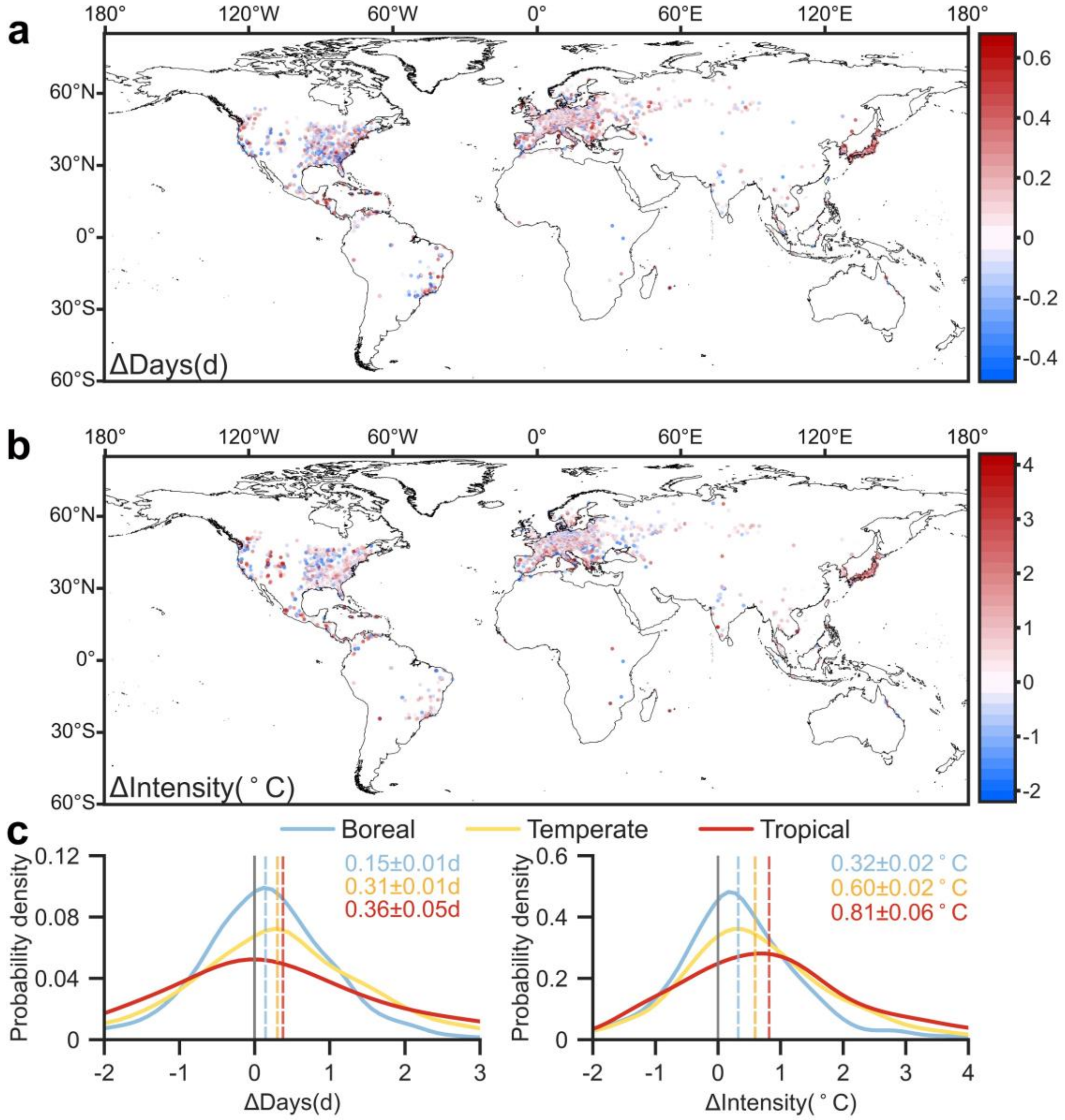

Supplementary Figure 7 Influence of urbanization on heat extremes of forest habitats in PUIs compared to background PAs. (a) Difference in the number of annual heat extreme days in protected forests between PUIs and background PAs, (b) difference of heat extreme intensity in protected forests between PUIs and background PAs, and (c) Kernel density distributions of difference in the number of annual heat extreme days and heat extreme intensity for protected boreal, temperate, and tropical forests, with values representing mean ± standard error of the mean (SEM).

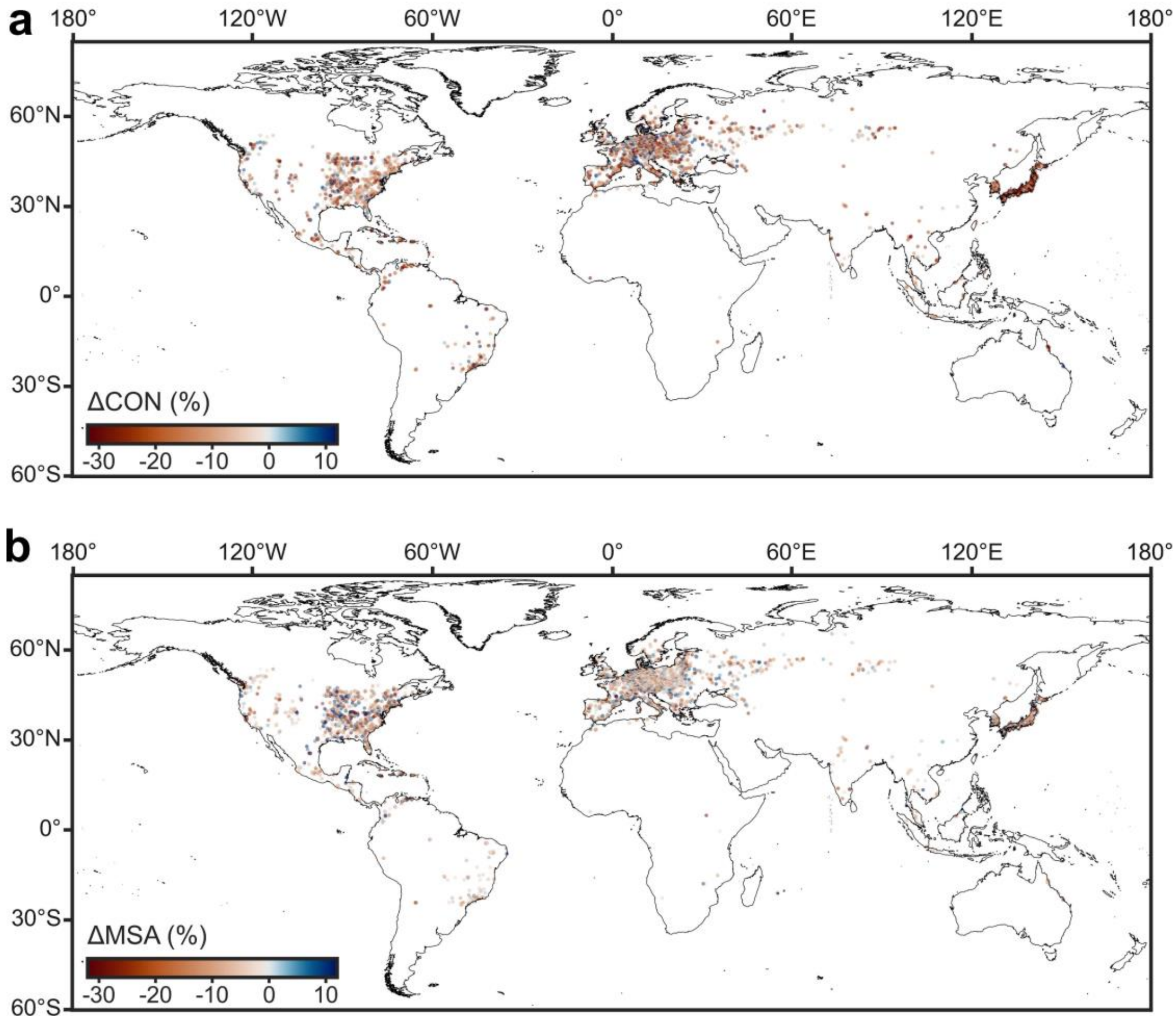

Supplementary Figure 8 Reductions in forest connectivity and mean species abundance in PUIs. (a) Spatial distribution of the connectivity deficit (ΔCON) between protected forests in PUIs and in background PAs, and (b) spatial distribution of mean species abundance (ΔMSA) between protected forests in PUIs and in background PAs

Supplemental References

1 Santoro, M. & Cartus, O. ESA biomass climate change initiative (Biomass_cci): Global datasets of forest above-ground biomass for the years 2010, 2017 and 2018, v2. *Cent. Environ. Data Anal* (2021).

2 Myneni, R., Knyazikhin, Y. & Park, T. MOD15A2H MODIS/Terra leaf area Index/FPAR 8-Day L4 global 500m SIN grid V006. *NASA EOSDIS Land Processes DAAC* (2015).

3 Lebakula, V. *et al.* LandScan global 30 arcsecond annual global gridded population datasets from 2000 to 2022. *Scientific Data* **12**, 495 (2025).

4 Meijer, J. R., Huijbregts, M. A., Schotten, K. C. & Schipper, A. M. Global patterns of current and future road infrastructure. *Environmental Research Letters* **13**, 064006 (2018).

5 Gong, P. *et al.* Annual maps of global artificial impervious area (GAIA) between 1985 and 2018. *Remote Sensing of Environment* **236**, 111510 (2020).

6 Potapov, P. *et al.* The global 2000-2020 land cover and land use change dataset derived from the Landsat archive: first results. *Frontiers in Remote Sensing* **3**, 856903 (2022).

7 Ma, J., Li, J., Wu, W. & Liu, J. Global forest fragmentation change from 2000 to 2020. *Nature Communications* **14**, 3752 (2023).

8 Grantham, H. *et al.* Anthropogenic modification of forests means only 40% of remaining forests have high ecosystem integrity. *Nature communications* **11**, 5978 (2020).

9 Schipper, A. M. *et al.* Projecting terrestrial biodiversity intactness with GLOBIO 4. *Global Change Biology* **26**, 760-771 (2020).

10 Mu, H. *et al.* An annual global terrestrial Human Footprint dataset (Version 8) [Dataset]. figshare. https://doi.org/10.6084/m9.figshare.16571064.v8 (2025).
