# Supplementary material for: Forests in protected areas worldwide face increased thermal pressure from urbanization
Source: Nat Commun. 2026 Jul 28;17:9171. doi: 10.1038/s41467-026-76226-8 (PMC13521939; doi:10.1038/s41467-026-76226-8)
Supplement: Supplementary file 2 [file 41467_2026_76226_MOESM2_ESM.pdf]

nature portfolio

Corresponding author(s): Xiyan Xu

Last updated by author(s): Jun 11, 2026

Reporting Summary

Nature Portfolio wishes to improve the reproducibility of the work that we publish. This form provides structure for consistency and transparency in reporting. For further information on Nature Portfolio policies, see our Editorial Policies and the Editorial Policy Checklist.

Statistics

For all statistical analyses, confirm that the following items are present in the figure legend, table legend, main text, or Methods section.

n/a	Confirmed	
☐	☒	The exact sample size (*n*) for each experimental group/condition, given as a discrete number and unit of measurement
☒	☐	A statement on whether measurements were taken from distinct samples or whether the same sample was measured repeatedly
☐	☒	The statistical test(s) used AND whether they are one- or two-sided<br>*Only common tests should be described solely by name; describe more complex techniques in the Methods section.*
☒	☐	A description of all covariates tested
☒	☐	A description of any assumptions or corrections, such as tests of normality and adjustment for multiple comparisons
☐	☒	A full description of the statistical parameters including central tendency (e.g. means) or other basic estimates (e.g. regression coefficient) AND variation (e.g. standard deviation) or associated estimates of uncertainty (e.g. confidence intervals)
☐	☒	For null hypothesis testing, the test statistic (e.g. *F*, *t*, *r*) with confidence intervals, effect sizes, degrees of freedom and *P* value noted<br>*Give P values as exact values whenever suitable.*
☒	☐	For Bayesian analysis, information on the choice of priors and Markov chain Monte Carlo settings
☒	☐	For hierarchical and complex designs, identification of the appropriate level for tests and full reporting of outcomes
☒	☐	Estimates of effect sizes (e.g. Cohen's *d*, Pearson's *r*), indicating how they were calculated

Our web collection on statistics for biologists contains articles on many of the points above.

Software and code

Policy information about availability of computer code

Data collection	Data collections were downloaded from Internet.
Data analysis	No custom code or algorithms were developed for this study. All analyses were performed using the open-source software QGIS (version 3.44.8; https://qgis.org).

For manuscripts utilizing custom algorithms or software that are central to the research but not yet described in published literature, software must be made available to editors and reviewers. We strongly encourage code deposition in a community repository (e.g. GitHub). See the Nature Portfolio guidelines for submitting code & software for further information.

Data

Policy information about availability of data

All manuscripts must include a data availability statement. This statement should provide the following information, where applicable:

- Accession codes, unique identifiers, or web links for publicly available datasets
- A description of any restrictions on data availability
- For clinical datasets or third party data, please ensure that the statement adheres to our policy

The datasets supporting the findings of this study were obtained from publicly available sources. The World Database on Protected Areas (WDPA) is available at https://www.protectedplanet.net/en/thematic-areas/wdpa?tab=WDPA. Global Urban Boundaries (GUB) data are available at https://data-starcloud.pcl.ac.cn/iearthdata/14. Annual Global Artificial Impervious Area (GAIA) maps are available at https://data-starcloud.pcl.ac.cn/iearthdata/13. Global Human Footprint data are available at https://doi.org/10.6084/m9.figshare.16571064. MODIS Aqua Land Surface Temperature (MYD11A2 Version 6) is available at https://doi.org/10.5067/

MODIS/MYD11A2.006. MODIS Leaf Area Index (MOD15A2H Version 6) is available at https://doi.org/10.5067/MODIS/MOD15A2H.006. ESA CCI land cover data are available at https://www.esa-landcover-cci.org. ESA CCI aboveground biomass data are available at https://climate.esa.int/en/projects/biomass. Global Mean Species Abundance data are available at https://www.globio.info/globio-data-downloads.

Research involving human participants, their data, or biological material

Policy information about studies with human participants or human data. See also policy information about sex, gender (identity/presentation), and sexual orientation and race, ethnicity and racism.

Reporting on sex and gender	Regarding Nature Communications' policy on sex and gender reporting, we confirm that our study does not involve human participants or gender-related analyses. Therefore, the sex and gender reporting guidelines are not applicable to this study.
Reporting on race, ethnicity, or other socially relevant groupings	The research design and methodology of this study did not involve the categorization or analysis of human race, ethnicity, or other socially relevant groups.
Population characteristics	The research design and methodology of this study did not address the collection and analysis of data on Population characteristics
Recruitment	This study did not involve the recruitment of human participants.
Ethics oversight	This study did not address the above and therefore does not involve an approved research protocol.

Note that full information on the approval of the study protocol must also be provided in the manuscript.

Field-specific reporting

Please select the one below that is the best fit for your research. If you are not sure, read the appropriate sections before making your selection.

☐ Life sciences ☐ Behavioural & social sciences ☒ Ecological, evolutionary & environmental sciences

For a reference copy of the document with all sections, see nature.com/documents/nr-reporting-summary-flat.pdf

Ecological, evolutionary & environmental sciences study design

All studies must disclose on these points even when the disclosure is negative.

Study description	This study used a comparative observational design with multi-source remote sensing datasets. Forest habitats within protected area–urban interfaces (PUIs) were compared with those in background protected areas (PAs) to quantify urban pressure and thermal effects. The analytical units were forest pixels sampled across global PAs from 1990–2018. All eligible observations were included in the analyses, and no experimental treatments or replicates were involved.
Research sample	Forest habitats were sampled within global protected areas (PAs) assigned IUCN categories I–VI and adjacent protected area–urban interfaces (PUIs). These raster-based samples were selected to quantify the effects of urbanization on protected forests. The study population represents forest habitats in globally protected areas and their surrounding urban influence zones. Analyses were conducted using publicly available remote sensing and spatial datasets. No organisms were used in this study, and no experimental manipulations were performed.
Sampling strategy	Forest habitats were identified from multi-source remote sensing datasets and sampled across all eligible global protected areas (PAs) and adjacent protected area–urban interfaces (PUIs). No a priori sample size calculation was performed because the study analyzed the complete set of available observations meeting the selection criteria. Sample size was therefore determined by the spatial extent and coverage of the datasets and is sufficient to represent global patterns of urban pressure and thermal responses in protected forests.
Data collection	All datasets were downloaded from publicly available sources and processed by the authors. Original data were collected by the respective providers using satellite sensors and geospatial monitoring systems.
Timing and spatial scale	Data were collected from publicly available remote sensing and geospatial datasets covering the period 1990–2018. Urban extent, protected area, and human footprint datasets were analyzed annually, whereas MODIS land surface temperature and leaf area index products were available at 8-day intervals and aggregated to annual metrics. Analyses were conducted globally using spatial datasets with native resolutions ranging from 500 m to 1 km, with some results displayed at a 2.5° spatial aggregation for visualization purposes.
Data exclusions	All non-forest pixels and protected areas classified outside IUCN categories I–VI or smaller than 1 km^2 were excluded from the analyses.
Reproducibility	This study is based on publicly available spatial datasets and does not involve laboratory or field experiments. All analyses were performed using reproducible computational workflows, and data sources are provided in the Data Availability statement to enable independent reproduction of the results.
Randomization	Not applicable. All forest pixels in protected areas and adjacent PUIs were analyzed observationally with no experimental grouping or randomization.

Blinding	Blinding was not relevant to this study, as all analyses were performed on publicly available spatial datasets and no manual or subjective measurements were made. There were no experimental group allocations that required blinding. All data processing and analyses were conducted programmatically.

Did the study involve field work? ☐ Yes ☒ No

Reporting for specific materials, systems and methods

We require information from authors about some types of materials, experimental systems and methods used in many studies. Here, indicate whether each material, system or method listed is relevant to your study. If you are not sure if a list item applies to your research, read the appropriate section before selecting a response.

Materials & experimental systems

n/a	Involved in the study
☒	☐ Antibodies
☒	☐ Eukaryotic cell lines
☒	☐ Palaeontology and archaeology
☒	☐ Animals and other organisms
☒	☐ Clinical data
☒	☐ Dual use research of concern
☒	☐ Plants

Methods

n/a	Involved in the study
☒	☐ ChIP-seq
☒	☐ Flow cytometry
☒	☐ MRI-based neuroimaging

Plants

Seed stocks	This study did not involve any seed stocks and other sources of plant material.
Novel plant genotypes	This study did not address methods for generating novel plant genotypes.
Authentication	This study did not involve any identification procedures using seed banks or generated new genotypes. It does not involve the description of any experiments used to assess the effects of mutations.
