# Supplementary material for: Forests in protected areas worldwide face increased thermal pressure from urbanization
Source: Nat Commun. 2026 Jul 28;17:9171. doi: 10.1038/s41467-026-76226-8 (PMC13521939; doi:10.1038/s41467-026-76226-8)
Supplement: Supplementary file 3 [file 41467_2026_76226_MOESM3_ESM.pdf]

nature portfolio

Peer Review File

Forests in protected areas worldwide face increased thermal pressure from urbanization

Corresponding Author: Professor Xiyan Xu

This file contains all reviewer reports in order by version, followed by all author rebuttals in order by version.

Version 0:

Reviewer comments:

Reviewer #1

(Remarks to the Author)
General comments
The paper quantifies how urbanization alters the thermal environment within protected forests. The authors report that urban expansion increased mean land-surface temperature by 0.63 °C and intensified thermal extremes by 0.81 °C in tropical protected forests. While the topic and findings are compelling, the conclusions require clearer articulation and more cautious interpretation before publication.
Major comments:
First, the approach used to quantify the impacts of urbanization on the thermal environment in protected forests needs to be described more clearly and in greater detail. For example, are the paired "protected forest" samples in the PUI and PA groups drawn from the same protected area, with the only distinction being their location relative to the 5-km buffer (one inside the buffer and one outside)? If so, this pairing strategy should be stated explicitly and justified. In addition, a more detailed schematic illustrating the sampling design and how the temperature difference is calculated would greatly improve clarity.
Second, the interpretation of the temperature contrast between PUI forests and PA forests needs to be clarified. Specifically, it is unclear whether the observed differences are primarily driven by (i) urbanization-associated forest degradation within PUI (e.g., reduced canopy cover or altered structure), (ii) edge effects resulting from greater fragmentation and a higher edge-to-interior ratio near urban areas, or (iii) thermal spillover from the urban heat island into adjacent forest. In addition, the Results section decomposes the PUI–PA temperature difference into three components, but the methodological basis for this partitioning is not sufficiently explained.
Third, the spillover of urban warming into nearby protected forests appears to be a key contribution of this study, but it is currently under-discussed. Please expand the Discussion by (i) situating your results within the existing literature on urban heat island spillover and microclimatic edge effects, and (ii) articulating the physical and ecological mechanisms that could explain why warming propagates into forests within urban buffers.

Detailed comments
L28-31: You need to summarize how to measure the spillover warm effects of urban expansion on adjacent forest.
L28-31: How to inspect thermal extremes? A simple explanation in the abstract can help readers to understand.
L28-31: Change "surface temperature" to "Land Surface Temperature".
L28-31: Different radii of the urban buffer yield different numbers. I strongly recommend clarifying the buffer radius.
L72-74: I am wondering if urbanization-induced landscape fragmentation and habitat loss, or spillover of warming, cause local extinction? I guess the landscape fragmentation and habitat loss, rather than the warmer thermal environment, play a major role.
L75: Please give more details about "urban heat spillover" in the previous studies. How do they define and measure this spillover? And what are the implications of "urban heat spillover"?
L81-82: The buffer radius is critical to identify the urban-protected area interface, as shown in the Fig.S1. How do you determine the buffer radius?
L104: "the global mean distance between urban areas and PAs shrinking by more than a third". How to inspect this result? Does urban expansion alone contribute to the closer distance? OR do both urban expansion and PA expansion contribute together?
L111-112: You defined urban pressure as a function of urban intensity and distance between urban and the target pixels. It is not surprising that PUI forest has a larger UP than PA forest, because PUI forest is closer to PA forest, as shown in the

Fig.S1. But how to understand the ΔUP2018 < 0 in the remaining 5% PUI forest?
L128-130 and L137-139: “urban pressure difference between forest habitats in PUIs and PAs declines significantly as PA size increases”, “the differences of urban pressure increase in forest habitats between PUI and PAs rise significantly with PA size”. I am confused a lot at this point. First, how to inspect the ΔUP changes in the temporal domain: that is ΔUP2018 - ΔUP1990? If the urban expansion caused closer distance between urban areas and protected forest in PUI contribute? Or does the higher urbanization intensity contribute? Second, and most importantly, how to explain these two reversed relationships with PA size?
L159-162: How to divide the temperature difference between PUI forest and PA forest into the three components (urbanization-induced direct forest canopy degradation, indirect spillover effects of urbanization, and local difference)?
L184-185: Why does PA size modulate the temperature disproportionally in PUI and PA forest, as shown in the fig.4b?

Reviewer #2

(Remarks to the Author)
The manuscript "Forests in protected areas worldwide face increased thermal pressure from urbanization" submitted for publication consideration to Nature Communications provides a global spatial assessment of the distribution of urban settlement areas and protected forest areas. The results show that protected forest areas located closer to urban settlements have on average elevated surface temperatures. The research is interesting and shows clear spatial patterns. I do have some comments which should be addressed particularly concerning the overall argument for some of the analyses.
Introduction
-Lines 54 ff. ..> The argument why it is important to understand how climate change and human activities affect these protected forests and their potentials to serve as thermal refuges for biodiversity is not entirely clear. The argument needs further elaboration. Also, climate change does not appear to be relevant in the further analyses.
- Lines 61f "...has led to ongoing forest loss and degradation in these PAs". Please provide more details and/or values to back up the argument.
- Lines 64f: "Many species depend on microclimates sustained by natural landscape, particularly PAs to function as thermal refuges under climate change," --> please provide more details and examples of such species.
Methodology
- 1990-2018 – time period chosen may be justified in a short sentence (although it is clear from the data available but also a short discussion on why this time period would be helpful)
- Lines 373 ff: So different distance within the buffer are used to define the PUI?
- A clear overview table should on data used with source, spatial resolution and temporal coverage should be provided
Results
- the analysis was done focussing on countries of lower and higher human development level   Why this was done, i.e. the argument behind is not entirely clear and should be developed in the introduction
- the analysis was also done to show differences between different forest types (tropical, temperate, boreal). Why this is important has not entirely been introduced and the argument needs further development in the introduction. Why is that important in the context of biodiversity.
Discussion
- Is distance the decisive factor or is it size and density of the urban area located close to the PA --> so what role plays size, building-density, industry, traffic? What about additional factors and the related impact on biodiversity?
- Particularly for forest areas, the surface temperature as the major indicator might not entirely capture the climatic temperature environment as air temperature is much more impacting the local environment conditions relevant for local biodiversity, no? A critical reflection on the use of surface temperature as a main indicator is needed.
- Lines 295ff: “Many tropical ectothermic species have evolved within narrow thermal ranges, often less than 2 °C between their realized habitat temperatures and upper thermal limits”   can this be further explained using specific examples?
- A transparent discussion on the limitations of the approach is missing.

Reviewer #3

(Remarks to the Author)
Title: Forests in protected areas worldwide face increased thermal pressure from urbanization

This study presented a research gap related to the consequences of urbanization on biodiversity conservation worldwide. Multiple analyses were conducted to assess how urbanization expansion is affecting protected areas in the different regions of the world. In general, the manuscript is very well written and clear. However, from our perspective, the manuscript fails to consider important connections between the research gap, method design, and the discussion of the results. In the following paragraphs, we emphasize the improvements in the study, considering major and minor aspects:
Research design
The manuscript presents, as the relevant research gap, that the thermal effects resulting from urbanization may affect biodiversity (lines 51-52; lines 75-79). However, the methods are not designed to evaluate biodiversity (species richness and diversity; species distribution) but the simple extent of protected areas (PAs). If the research gap is centered on biodiversity or the extent of structured habitat for species distribution, the methods should be designed to address this research question.

Database
The manuscript presents PAs as a proxy of forest habitats. The PAs present different vegetation cover around the world, and in some regions, especially in tropical regions, this cover provides a rich habitat for species distribution (such as ruprestrian grassland or even the cerrado in Brazil). Therefore, the results obtained for “forest habitats” can be overestimated,

considering that not all PAs are covered only by forests.
Moreover, the PAs from UNEP-WCMC represent different protected territories, including indigenous lands, conservation units that permit or not the presence of human occupation. The manuscript considers a unique category of PA that may not be realistic for the discussion of the results.

Methods
The manuscript presents a proximity analysis to evaluate the effects on biodiversity/ecosystems. However, it is not sufficient to provide concrete evidence on the topic by not providing a landscape analysis, connectivity, and fragmentation assessment. The authors mention the size of PAs as an important aspect, but their connectivity is of most importance for biodiversity conservation. The formation of ecological corridors, even between small PAs, is relevant for conserving biodiversity and the integrity of ecosystems, and this could play an important role in designing urban PAs and assessing their thermal effects.
Moreover, the proximity analysis may be insufficient to evaluate the effects on biodiversity, requiring, for example, the analysis of species distribution across PAs when assessing the thermal effects of urbanization.

Minor comments
Introduction
The introduction fails to critically review the literature, considering aspects of ecosystem conservation close to urban areas. The discussion of cultural ecosystem services, such as recreation, environmental education provided by the PAs close to urban areas, needs to be described in the introduction as well. Some very minor grammar adjustments are required (lines 44 and 64), and adding sources for the cited afforestation efforts in line 50.
Results
In general, it is not clear that the term 'PAs' refers only to the portion of protected areas that do not overlap with PUIs. I would recommend including an additional acronym for the PA area without PUIs. Readers might not be able to clearly distinguish this difference, as 'PAs' as an acronym for protected areas could refer to the whole PA area.
When the attributions of surface temperature rise are presented (line 160), I would make the language tone used lighter, as there are certain limitations towards those findings.

Discussion
The discussion could describe the possible causal mechanisms between PA size and urban pressure. The general consideration for policy implication should not be generic (as mentioned in the abstract) but grounded in the results. Furthermore, presenting limitations of the work and future research directions would enhance the study.

Methods
The methods section and figures are clear. Data sources are cited, and supplementary information provides further details about the analysis.
As there are many different steps and parts of the analysis, it would help the reading experience to provide a figure with an overview of the different methodological steps taken. As in the results sections, we recommend substituting the acronym PA for a different one when referring to the portion without PUI.
Moreover, the method used for attribution of surface temperature change presented in the results (line 160) is not cited in the methods. They should be at least cited in the 'Alteration of the thermal environment in forest habitats' section, including a reference to supplementary notes (note S3). Finally, if there are any limitations from the methods used and databases, they should be presented.

Reviewer #4

(Remarks to the Author)
I co-reviewed this manuscript with one of the reviewers who provided the listed reports. This is part of the Nature Communications initiative to facilitate training in peer review and to provide appropriate recognition for Early Career Researchers who co-review manuscripts.

Version 1:

Reviewer comments:

Reviewer #1

(Remarks to the Author)
Most of my previous comments have been addressed satisfactorily.
I have one additional comment:
I would be interested to know what the LST comparison would show if unprotected forests inside and outside the urban buffers were also analyzed. Moreover, what differences of LST exist between protected and unprotected forests within the urban buffer?

More minor comments:
L31: "surface temperature" is not equivalent to LST, please direct use LST.
L32 "mean surface temperature" is not clear, is annual mean values of daily LST?

Reviewer #2

(Remarks to the Author)
The manuscript has improved and my previous comments have been addressed, mostly sufficiently. Minor comments remain:
• In the Supplementary Material: The Mu et al. Reference is not included in the Manuscript. The publication with Scientific Data covers 2000-2018. https://doi.org/10.1038/s41597-022-01284-8 Was there an update? Please add reference.
• I suggest you define Protected areas right at the beginning of the manuscript and clearly highlight that you mean in your case forest PA. Otherwise the use of the term and abbreviation raises confusion.
• Line 56: "Nations Strategic Plan for Forests (UNSPF) and the Bonn Challenge14,15" Can you please briefly explain what these efforts quantitatively mean, any thresholds to be reached?
• Line 107ff: You need to describe here clearly already why you primarily focus at the 5km buffer.
• Lines 202ff: When do you consider a PA as large and when small?
• For the discussion section, I suggest to write out abbreviations again at the beginning, just to remind the reader about their meaning.
• Line 230: Please specify "are often". What do you mean by often?
• Lines 305ff: The discussion on the limitation of the approach concerning the use of surface temperature as a proxy for temperature increase versus air temperature is too narrow and should be expanded. Please, include also references to back up your argument.
• Please write out or explain abbreviations in the figure legends in your figures.

Reviewer #3

(Remarks to the Author)
Second review - General comments from the reviewers
The authors made several amendments to the manuscript. The research scope is clearer and better justified. The authors improved the clarity in the methods section. Additional analysis of impacts was conducted, and some limitations were clarified. However, we still identify some important issues, detailed below, among the three major topics.
1) The research gap needs to ground the research design
Existing literature focused on temperature elevation; the authors argue that there is a gap in studies analyzing thermal impacts on protected landscapes. However, the authors only quantify the temperature change, without an analysis of the actual impacts derived from it (change in the environmental quality in the affected habitats).
2) Use of terms: Protected forests X PAs in general
Given the fact that the conducted analysis considered only forest pixels within protected areas (lines 381-383), the results cannot be directly extrapolated to the whole PA systems. The authors need to be more careful when concluding results, considering this limitation. For example, in lines 258 and 296 of the revised paper, results are cited as impacts on the whole PAs and not only on protected forests.
3) The term 'background PAs' is confusing and cannot be used as a robust comparison for impact assessment
The authors argue that they analyze the impacts of urbanization on the local thermal capacity of protected forests. However, the thermal capacity or impact in PUI is not properly compared to control areas to demonstrate the real impact of thermal effects. The comparison with the "internal region" of each PA is biased by other cooling effects of dense forests and does not provide a robust threshold without the effects of urbanization and other pressures surrounding the PAs (See impact assessment and BACI methods)
The term 'background PAs' is confusing, because it treats the PAs without any overlap with urban areas, and those that do not suffer any effect from it in the same way as PAs with some overlap with urban areas. While PAs farther from urban areas and/or with similar properties could be treated as a control group for a robust assessment, this is not done.
The authors compared biodiversity indicators (connectivity and mean of species abundance) only between PUIs and background PAs. This represents an issue, because PAs that do not overlap with urban areas (without PUIs) and PAs that partially overlap with urban areas are considered the same.
The authors directly attribute the differences in connectivity and species abundance to the urban effects, without considering other drivers for it, such as other human disturbances (infrastructure projects, mining, agriculture) and the impacts of the edge effect itself, where the interior of PAs will naturally be more conserved and suffer less from any disturbances than the edges.
Furthermore, the urban effect on the thermal impact needs to be isolated from other drivers of temperature change. The temperature change cannot be fully attributed to urban effects, because there are also thermal effects from other drivers, such as the edge effect due to fragmentation of the landscape and other human disturbances in the surroundings.

Because of these important topics explained above, we recommend a major review to address those issues in the analysis conducted.

Reviewer #4

(Remarks to the Author)
I co-reviewed this manuscript with one of the reviewers who provided the listed reports. This is part of the Nature Communications initiative to facilitate training in peer review and to provide appropriate recognition for Early Career Researchers who co-review manuscripts.

Version 2:

Reviewer comments:

Reviewer #1

(Remarks to the Author)
My previous comments have been generally addressed. However, I still have the following concerns that may need to be further clarified or strengthened, and hopefully that can help improve the study.
1. Conceptual ambiguity and imprecise terminology
Some key concepts and terms used in the manuscript remain ambiguous or insufficiently rigorous.
First, the manuscript interprets the local land surface (i.e., canopy) cooling effect of forests as “thermal refugia” or “thermal buffering capacity.” This interpretation is not entirely accurate. In ecological and climatological contexts, these terms typically refer to the ability of forests to regulate sub-canopy microclimate and buffer against ambient temperatures, including both cooling under hot conditions and warming under cold conditions (see reference like this: https://www.nature.com/articles/s41559-019-0842-1). However, MODIS LST represents canopy surface temperature and does not capture the microclimatic conditions beneath the forest canopy. Therefore, using these terms in the current context may be conceptually misleading and should be reconsidered or more carefully qualified.
Second, terms such as “macroclimate warming” and “microclimatic conditions” are used somewhat loosely. These terms should be clearly defined and used consistently.
Third, expressions such as “human pressure,” “urban pressure,” and “thermal pressure” are vague and potentially ambiguous. These terms would benefit from clearer definitions or replacement with more precise, measurable descriptors.
2. Attribution analysis lacks depth and clarity
While I generally agree that the impacts of urbanization on LST in PUIs can be attributed to both direct human disturbance and urban heat spillover, the attribution analysis presented in the main text (L174–190) remains insufficiently convincing. The quantitative decomposition (e.g., 0.18 °C attributed to canopy degradation, 0.13 °C to urban heat spillover, and 0.07 °C to background differences) seems interesting, but the methodological basis for this partitioning is not adequately explained. The authors are encouraged to provide a more detailed explanation of the attribution framework, including assumptions, uncertainties, and limitations, to strengthen the robustness and interpretability of these results.
3. Urban heat spillover is not explicitly quantified
The manuscript introduces urban heat spillovers as a key mechanism, but it is not directly quantified. Instead, it appears to be inferred as the residual after subtracting the effects of direct canopy degradation from the total LST difference.
Moreover, urban heat spillover is described as driven by atmospheric processes such as advection and diffusion (L87-88 in the main text), yet no analysis of wind patterns, atmospheric circulation, or related variables is included to support this mechanism.
At a minimum, this limitation should be explicitly acknowledged in the Discussion. Clarifying that the current estimation of urban heat spillover is indirect and subject to uncertainty would improve the transparency and credibility of the study.
Overall, addressing the above issues is needed to improve its conceptual rigor and the robustness of its conclusions.

Reviewer #2

(Remarks to the Author)
My point on the reference data for the human foodprint index was not solved and still the old reference is in the manuscript although it is argued that the data was updated to 2024. In the supplementary Material, the Mu et al. reference appears in Table S6 without indication of year and the reference is not included at all in the supplementary Material. Also, in Table S6 it is called index but authors use intensity.

In the manuscript the old refernce with No 42 is still used: Mu, H. et al. A global record of annual terrestrial Human Footprint dataset from 2000 to 2018. Scientific Data 9, 176 (2022).

I am very surprised that this was not solved during revisions.

Reviewer #3

(Remarks to the Author)
The authors made a comprehensive review, addressing most of the topics we have pointed out. The additional analyses made on the multiple possible drivers for temperature change enhanced the robustness of the study.
Before considering the manuscript for final acceptance, we suggest only minor reviews: (i) the inclusion of a separate session on limitations of the work, describing limitations such as the difficulty of separating the influence of urban island effect from other activities such as industries and infrastructure projects on the impacts on PAs described; (ii) review in the language and final tone of the manuscript, aligned with these limitations.

Reviewer #4

(Remarks to the Author)
I co-reviewed this manuscript with one of the reviewers who provided the listed reports. This is part of the Nature

Communications initiative to facilitate training in peer review and to provide appropriate recognition for Early Career Researchers who co-review manuscripts.

Version 3:

Reviewer comments:

Reviewer #1

(Remarks to the Author)
I have carefully reviewed the authors' responses and the revised manuscript and I am satisfied with the revisions made, as they have adequately addressed the concerns raised in the previous rounds of review. Therefore, I am pleased to recommend this paper for publication in its current form.

Reviewer #2

(Remarks to the Author)
My comments were addressed. Thanks.

Reviewer #3

(Remarks to the Author)
The authors added a paragraph describing the limitations of the work, addressing the main recommendation from the previous round of review.
A minor point of review is the need to define the new acronym - UHI. From our point of view, after this small change, the manuscript can be considered for publication.

Reviewer #4

(Remarks to the Author)
I co-reviewed this manuscript with one of the reviewers who provided the listed reports. This is part of the Nature Communications initiative to facilitate training in peer review and to provide appropriate recognition for Early Career Researchers who co-review manuscripts.

Open Access This Peer Review File is licensed under a Creative Commons Attribution 4.0 International License, which permits use, sharing, adaptation, distribution and reproduction in any medium or format, as long as you give appropriate credit to the original author(s) and the source, provide a link to the Creative Commons license, and indicate if changes were made.
In cases where reviewers are anonymous, credit should be given to 'Anonymous Referee' and the source.
The images or other third party material in this Peer Review File are included in the article’s Creative Commons license, unless indicated otherwise in a credit line to the material. If material is not included in the article’s Creative Commons

license and your intended use is not permitted by statutory regulation or exceeds the permitted use, you will need to obtain permission directly from the copyright holder.
To view a copy of this license, visit https://creativecommons.org/licenses/by/4.0/

We appreciate the four reviewers for taking the time to review our manuscript and provide valuable comments. We addressed all the comments and made revisions in the manuscript accordingly. Please find our responses to the reviewers below. Our responses are in blue and the corresponding revisions in the manuscript are in purple. All the revisions are highlighted in yellow in the revised manuscript.

Reviewer #1:

General comments

The paper quantifies how urbanization alters the thermal environment within protected forests. The authors report that urban expansion increased mean land-surface temperature by 0.63 °C and intensified thermal extremes by 0.81 °C in tropical protected forests. While the topic and findings are compelling, the conclusions require clearer articulation and more cautious interpretation before publication.

Authors: Thank you for your constructive comments which have helped us improve the manuscript. We addressed all the comments and made revisions according to your comments.

Major comments:

First, the approach used to quantify the impacts of urbanization on the thermal environment in protected forests needs to be described more clearly and in greater detail. For example, are the paired "protected forest" samples in the PUI and PA groups drawn from the same protected area, with the only distinction being their location relative to the 5-km buffer (one inside the buffer and one outside)? If so, this pairing strategy should be stated explicitly and justified. In addition, a more detailed schematic illustrating the sampling design and how the temperature difference is calculated would greatly improve clarity.

Authors: We added a schematic diagram illustrating the method design (Figure S1D). A PA fully falling within the buffer zone of an urban area is classified as a PUI. Otherwise, the part of a PA falling within the buffer zone is classified as a PUI, and outside the buffer zone is classified as a background PA (PAB). Within each 0.25° window, we compared all forest pixels located in PUIs with that in PABs. We assumed that background climate and background human activity levels are similar within each 0.25° window, such that the observed differences between PUIs and PABs can be attributed to the additional influence of urbanization.

We further clarified this approach in the Methods section.

We assumed that the background climate for paired PUIs and background PAs was similar in each 0.25° window. We did not differentiate management regimes of PAs.

At this local to regional scale of 0.25° window, management intensity and effectiveness, which are shaped by local political and socio-economic conditions and shift over time, are also assumed to be similar for PUIs and nearby background PAs. Disturbance caused by urbanization on forests in background PAs was assumed to be minimum. Forests in PUIs, however, were assumed to be exposed to more external disturbances caused by urbanization than protected forests in paired background PAs. (L440-447)

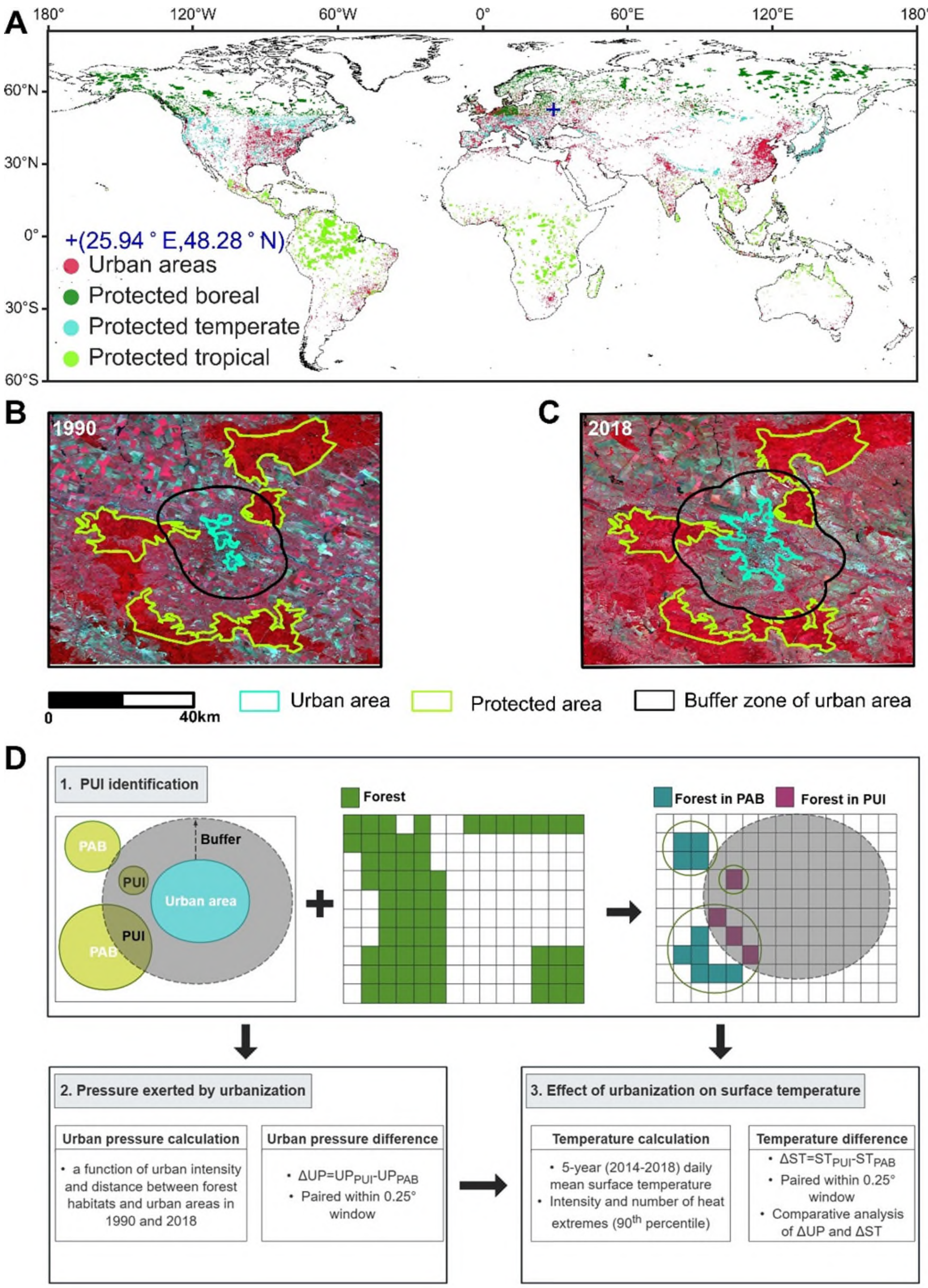

Figure S1 Global urban areas and protected forests. (A) Spatial distribution of global urban areas and protected boreal, temperate and tropical forests in 2018, (B) example

of Landsat-5 false-color composite of near infrared, red, and green bands at 30-m resolution for urban areas, protected areas and buffer zone of urban area in Ukraine (25.94°E，48.28°N) on August, 1990, (C) example of Landsat-8 false-color composite for the same area, on August, 2018, (D) diagram illustrating the main research steps to identify the protected area-urban interface (PUI) and their relative position with background protected area (PAB), and calculate urban pressure (UP) and effect of urbanization on surface temperature (ST).

Second, the interpretation of the temperature contrast between PUI forests and PA forests needs to be clarified. Specifically, it is unclear whether the observed differences are primarily driven by (i) urbanization-associated forest degradation within PUI (e.g., reduced canopy cover or altered structure), (ii) edge effects resulting from greater fragmentation and a higher edge-to-interior ratio near urban areas, or (iii) thermal spillover from the urban heat island into adjacent forest. In addition, the Results section decomposes the PUI–PA temperature difference into three components, but the methodological basis for this partitioning is not sufficiently explained.

Authors: We have clarified the interpretation of the temperature contrast between PUI forests and background PA (defined as PAB in the revised manuscript to distinguish it from PA in PUI, please see our response above) forests in the Results section. We acknowledge that forest fragmentation can affect local surface temperature. However, the fragmentation is reflected in reduced forest cover, leaf area index and canopy height, which affects surface temperature through canopy biophysical changes already being accounted for in our analysis of direct effects.

In addition, we provided a more detailed description of the attribution method and its underlying assumptions in the Supplementary Information Note S3.

We further quantified the relative contributions of the direct effect (e.g., canopy degradation caused by urbanization) (Supplementary Information Note S2, Figure S4 and S5), indirect effect (e.g., urban heat spillovers), and residual component reflecting effects of topographic, edaphic, or other unaccounted factors to the thermal difference between PUIs and background PAs (Supplementary Information Note S3, Figure S6). We found that in the 0.38 ± 0.01 °C difference, about 0.18 °C is attributed to urbanization-induced direct forest canopy degradation, about 0.13 °C is attributed to indirect effects of urban heat spillover, and the remaining 0.07°C is introduced by the differences in factors such as background topography and soil properties between PUIs and background PAs (Supplementary Information Note S3). These results underscore the dual roles of direct forest degradation and indirect spillovers following urban expansion in shaping the surface temperature of forests in PUIs.（L171-182）

Note S3 Attribution of urbanization-induced surface warming

To assess the extent to which urbanization contributes to surface temperature increases in PUIs, we estimated the temperature change induced by urban pressure as the product of its sensitivity and magnitude:

$$\Delta \mathrm{ST}=\left(\frac{\partial \mathrm{ST}}{\partial \mathrm{UP}}\right)_{\mathrm{PA}} \Delta \mathrm{UP} +\varepsilon \tag{3}$$

where $\left(\frac{\partial \mathrm{ST}}{\partial \mathrm{UP}}\right)_{\mathrm{PA}}$ represents the overall sensitivity of surface temperature to urban pressure in PAs. This sensitivity was estimated using the Theil–Sen slope in each 0.25° grid cell. ΔUP is the difference in urban pressure between PUIs and background PAs in each 0.25° grid cells, reflecting the extent of forest habitat degradation associated with urban pressure. ε is the residual term, indicating residual biases introduced by factors such as topography, soil properties, and remote sensing uncertainties.

$\left(\frac{\partial \mathrm{ST}}{\partial \mathrm{UP}}\right)_{\mathrm{PA}} \Delta \mathrm{UP}$ provides an estimate of surface temperature change attributable specifically to urban pressure. To better interpret the underlying processes, we further decomposed this term into two components: (i) direct effects driven by forest canopy degradation ($\Delta \mathrm{ST}_{\mathrm{direct}}$), and (ii) indirect effects arising from urban heat island (UHI) spillover and other spillover effects of human activities ($\Delta \mathrm{ST}_{\mathrm{indirect}}$):

$$\Delta \mathrm{ST}_{\mathrm{UP}} = \left(\frac{\partial \mathrm{ST}}{\partial \mathrm{UP}}\right)_{\mathrm{PA}} \Delta \mathrm{UP} =\Delta \mathrm{ST}_{\mathrm{direct}} + \Delta \mathrm{ST}_{\mathrm{indirect}} \tag{4}$$

The direct effects of urban pressure on surface temperature ($\Delta \mathrm{ST}_{\mathrm{direct}}$) from structural canopy degradation, including reductions in leaf area index (LAI) (horizontal canopy loss), canopy height (CH) (vertical structural decline), and forest cover (FC) (overall extent of forest loss). These changes alter surface energy budget by reducing turbulent heat flux and exposing darker surfaces with lower albedo, thereby contributing directly to land surface warming in forests. To quantify this, we estimated $\Delta \mathrm{ST}_{\mathrm{direct}}$ by aggregating the warming contributions associated with each canopy variable:

$$\Delta \mathrm{ST}_{\mathrm{direct}}=\left(\frac{\partial \mathrm{ST}}{\partial \mathrm{LAI}}\frac{\partial \mathrm{LAI}}{\partial \mathrm{UP}} + \frac{\partial \mathrm{ST}}{\partial \mathrm{CH}}\frac{\partial \mathrm{CH}}{\partial \mathrm{UP}} + \frac{\partial \mathrm{ST}}{\partial \mathrm{FC}}\frac{\partial \mathrm{FC}}{\partial \mathrm{UP}}\right)_{\mathrm{PA}} \Delta \mathrm{UP} \tag{5}$$

Where $\left(\frac{\partial ()}{\partial \mathrm{UP}}\right)_{\mathrm{PA}}$ represent the sensitivities of leaf area index, canopy height and forest cover to urban pressure in PAs. We estimated these sensitivities using the Theil–Sen slope in each 0.25° grid cell, based on MODIS LAI data (MCD15A2H) at 0.01° resolution from 2018 and forest cover and height data from the GLAD Global Land Cover and Land Use Change dataset from 2020. $\left(\frac{\partial \mathrm{ST}}{\partial ()}\right)_{\mathrm{PA}}$ represent the sensitivities of surface temperature to leaf area index, canopy height, and forest cover, respectively, in PAs. These sensitivities were estimated by fitting multiple linear regression models of

surface temperature against leaf area index, canopy height, and forest cover across protected forest pixels within each 0.25° grid cell, thereby isolating their independent associations with surface temperature.

The indirect effects of urban pressure include UHI spillover as well as other spillover effects of human activities, such as increased heat emissions, altered irrigation practices, or reduced wind flow from nearby built-up areas. These processes can alter the thermal environment of forest habitats without involving direct canopy degradation. Due to the lack of a direct attribution method for indirect effects, we estimated $\Delta ST_{indirect}$ as the residual difference between the total temperature change attributable to urban pressure and the portion explained by direct canopy degradation.

At the global scale, the mean observed surface temperature difference between PUIs and background PAs was 0.38 ± 0.01 °C. Of this, 0.31 ± 0.01 °C (contribution rate = 81.6%) was attributed to urban pressure (Figure S6A), in which 0.18 °C was linked to direct effects of forest canopy degradation (Figure S6B), with contributions by loss in leaf area index (0.07 ± 0.01 °C, 18.4%), canopy height (0.05 ± 0.01 °C, 13.2%), and forest cover (0.06 ± 0.01 °C,15.8%). The remaining 0.13 ± 0.01 °C (34.2%) was attributed to indirect effects (Figure S6C), including potential UHI spillover and other non-structural human activity spillovers. These findings underscore the dual contribution of direct forest degradation and indirect urban influences in shaping the surface temperature of forests in PUIs.

Third, the spillover of urban warming into nearby protected forests appears to be a key contribution of this study, but it is currently under-discussed. Please expand the Discussion by (i) situating your results within the existing literature on urban heat island spillover and microclimatic edge effects, and (ii) articulating the physical and ecological mechanisms that could explain why warming propagates into forests within urban buffers.

Authors: We added the related discussion according to your suggestion.

Excessive warming in PUIs is attributed to direct effects of canopy degradation and indirect urban heat spillovers. Canopy degradation in PUIs is reflected in the lower leaf area index, forest cover, and canopy height, which modifies surface energy budget and weakens local cooling capacity directly. In addition, canopy degradation increases forest edge exposure and thus expands forest contact with urban heat indirectly. Extensive impervious urban surfaces enhance solar radiation absorption and suppress evapotranspiration, leading to heat accumulation that warms near-surface air, and the excessive heat can be transported beyond urban boundaries through advection and diffusion39. As urban expands beyond original city margins, the transfer of accumulated heat into surrounding wildland areas is further amplified38,41. Furthermore, anthropogenic heat from transportation and infrastructures can propagate

into distant wildland areas along transportation51. While the different forms of heat spillover along the urbanization were documented, their contribution to wildland thermal environment has not yet been quantified. In this study, we disentangled the indirect impacts of urban heat spillover using a residual-based approach, however, observations and simulations are needed to better understand the underlying physical processes. (L278-293)

Detailed comments

L28-31: You need to summarize how to measure the spillover warm effects of urban expansion on adjacent forest.

L28-31: How to inspect thermal extremes? A simple explanation in the abstract can help readers to understand.

L28-31: Change “surface temperature” to “Land Surface Temperature".

L28-31: Different radii of the urban buffer yield different numbers. I strongly recommend clarifying the buffer radius.

Authors: We rewritten this part of the abstract according to your suggestions.

The thermal impacts of urban pressure were assessed by comparing satellite-derived mean land surface temperature (LST) and thermal extremes inferred by the 90th-percentile maximum LST of protected forests within and beyond a 5-km buffer zone outside urban boundaries. (L25-28)

L72-74: I am wondering if urbanization-induced landscape fragmentation and habitat loss, or spillover of warming, cause local extinction? I guess the landscape fragmentation and habitat loss, rather than the warmer thermal environment, play a major role.

Authors: We clarified the potential impacts of habitat fragmentation and warming on local species according to your suggestion.

This spillover warmth driven by the transport of urban heat through atmospheric advection and diffusion intensifies thermal stress on species in PAs, while urbanization-induced habitat destruction and fragmentation reduce habitat connectivity and constrains climate-driven dispersal, together increasing the risk of local extinctions (L85-89)

L75: Please give more details about “urban heat spillover” in the previous studies. How do they define and measure this spillover? And what are the implications of “urban heat spillover”?

Authors: We added more details about urban heat spillover according to your suggestion.

Despite growing recognition of urban heat spillover, existing studies quantifying temperature elevation extending from urban areas to their surrounding rural areas mainly focused on its implications for human health and urban-rural resilience. However, the thermal impacts of urban heat spillover on natural and semi-natural ecosystems, particularly protected forest ecosystems, remain underexplored. (L90-94)

L81-82: The buffer radius is critical to identify the urban-protected area interface, as shown in the Fig.S1. How do you determine the buffer radius?

Authors: We have tested multiple buffer distances (5, 10, 25, and 50 km) and justified the rationale for buffer selection in the Methods. PUIs identified using the 5-km buffer showed the clearest signals of canopy degradation and warming relative to larger radii. We also clarified the buffer-radius setting accordingly and refer to Materials and Methods in this sentence.

…mapped as the overlapping regions between PAs and urban buffer zones delineated at multiple distances (5, 10, 25, and 50 km PUIs) from urban boundaries (Materials and Methods: Identification of PAs, urban areas, and their interfaces) (L97-100)

L104: "the global mean distance between urban areas and PAs shrinking by more than a third". How to inspect this result? Does urban expansion alone contribute to the closer distance? OR do both urban expansion and PA expansion contribute together?

Authors: We restricted our distance analysis to PAs established before 1990 (Supplementary Information Note S1) so that changes in distance reflect the effects of urban expansion toward already established PAs. We have additionally clarified this in the Results section.

More than 95% of PAs established before 1990 have experienced approaching urban expansion between 1990 and 2018, … (L125-127)

L111-112: You defined urban pressure as a function of urban intensity and distance between urban and the target pixels. It is not surprising that PUI forest has a larger UP than PA forest, because PUI forest is closer to PA forest, as shown in the Fig.S1. But how to understand the $\Delta UP2018 < 0$ in the remaining 5% PUI forest?

Authors: The $\Delta UP2018$ is positive in the vast majority (>95%) of cases. However, in a small number of situations, background PA forests (PAB) may still experience higher urban pressure. Although these PA forests are outside the urban buffer, they can be simultaneously influenced by several nearby cities (Figure R1 below). When these influences are combined, the overall urban pressure can exceed that experienced by PUIs, which explains why $\Delta UP2018$ can be negative in these cases.

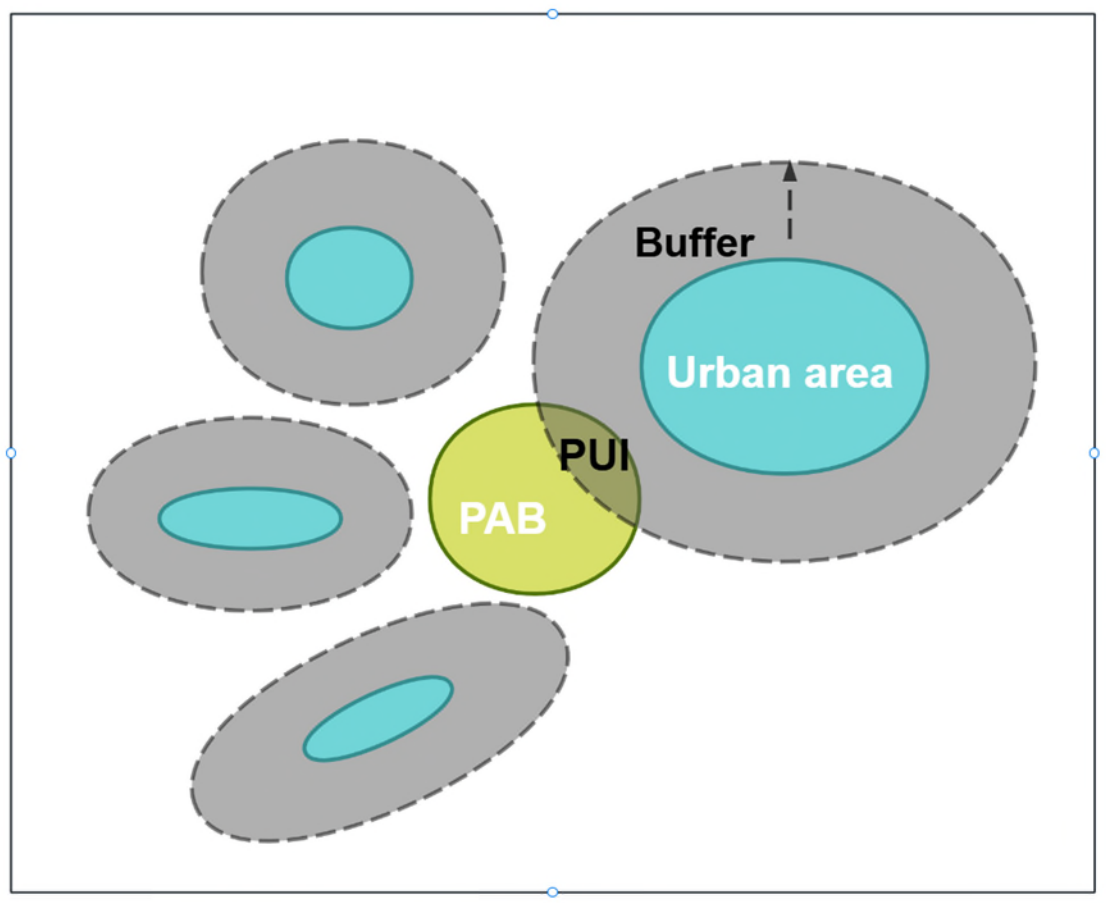

Figure R1 Relative position of PUI and PAB in which PAB forest can experience higher urban pressure than PUI forest

L128-130 and L137-139: "urban pressure difference between forest habitats in PUIs and PAs declines significantly as PA size increases", "the differences of urban pressure increase in forest habitats between PUI and PAs rise significantly with PA size". I am confused a lot at this point. First, how to inspect the ΔUP changes in the temporal domain: that is ΔUP2018 - ΔUP1990?

Authors: The urban pressure difference between forest habitats in PUIs and PAs is defined as $\Delta UP = UP_{PUI} - UP_{PAB}$. The temporal change in ΔUP is defined as $\Delta UP_{2018} - \Delta UP_{1990}$, which is explicitly stated in the manuscript (L136 and L152).

If the urban expansion caused closer distance between urban areas and protected forest in PUI contribute? Or does the higher urbanization intensity contribute?

Authors: Urban pressure is calculated as a nonlinear function of urbanization intensity and distance to urban areas (Equation 1 in Materials and Methods), which prevents a linear separation of the effects of urbanization intensity and distance to urban areas. We conducted an additional scenario-based analysis to examine the relative roles urbanization intensity and distance to urban area. We recalculated ΔUP_{2018} under two scenarios: (S1) retaining only pre-existing urban pixels (urban intensity > 0 in 1990), ($\Delta UP_{2018} - \Delta UP_{1990}$) reflects changes associated with increasing urbanization intensity, and (S2) retaining only newly emerged urban pixels (urban intensity = 0 in 1990 and > 0 in 2018), ($\Delta UP_{2018} - \Delta UP_{1990}$) reflects distance reduction due to urban expansion. In both cases, ΔUP1990 was calculated using the urban distribution and intensity of 1990.

We showed changes in ΔUP ($\Delta UP_{2018} - \Delta UP_{1990}$) were 1.02% and 0.73%, for scenarios S1 and S2, respectively. This analysis indicates that changes associated with urbanization intensity cause a larger ΔUP change than distance reduction alone.

…, mainly driven by rising urbanization intensity within pre-existing urban areas.

(L154-156)

To examine temporal changes in urban pressure, we calculated ΔUP changes between 1990 and 2018 (ΔUP_{2018}-ΔUP_{1990}). Because urban pressure is defined as a nonlinear function of urbanization intensity and distance to urban areas, we conducted an additional scenario-based analysis to examine the relative roles urbanization intensity and distance to urban area. We recalculated ΔUP_{2018} under two scenarios: (S1) retaining only pre-existing urban pixels (urban intensity > 0 in 1990), (ΔUP_{2018}-ΔUP_{1990}) reflects changes associated with increasing urbanization intensity, and (S2) retaining only newly emerged urban pixels (urban intensity = 0 in 1990 and > 0 in 2018), (ΔUP_{2018}-ΔUP_{1990}) reflects distance reduction due to urban expansion. In both cases, ΔUP1990 was calculated using the urban distribution and intensity of 1990. We showed changes in ΔUP (ΔUP_{2018}-ΔUP_{1990}) were 1.02% and 0.73%, for scenarios S1 and S2, respectively. This analysis indicates that changes associated with urbanization intensity cause a larger ΔUP change than distance reduction alone. (L462-474)

Second, and most importantly, how to explain these two reversed relationships with PA size?

Authors: We added relevant explanations in the Discussion.

We show that urban pressure in PUIs was higher in smaller PAs, whereas PUIs of larger PAs exhibited greater temporal increases in urban pressure since 1990. These contrasting patterns arise because small PAs are often located closer to cities (Supplementary Information Note S1) and thus experience higher baseline urban pressure, while further urban expansion around them is spatially and temporally constrained. Since the remaining non-PUI areas of small PAs are much smaller than those of larger PAs, small PAs are more likely to be fully exposed to urban pressure. In contrast, large PAs are typically located farther from cities with extensive boundaries, leaving greater potential for urbanization in their surrounding areas and leading to greater temporal increases in urban pressure as urbanization progresses. (L228-237)

L159-162: How to divide the temperature difference between PUI forest and PA forest into the three components (urbanization-induced direct forest canopy degradation, indirect spillover effects of urbanization, and local difference)?

Authors: We provided a more detailed description of the attribution framework and its underlying assumptions in Supplementary Information Note S3. Please see our response to you second major Comment.

L184-185: Why does PA size modulate the temperature disproportionally in PUI and PA forest, as shown in the fig.4b?

Authors: We added relevant explanations in this part of results.

This suggests that in small PAs, both PUIs and background PAs are often exposed to urban pressure, weakening original thermal buffering, whereas large PAs retain extensive interior areas distant from urban pressure and thus maintain stronger buffering capacity. (L202-205)

Reviewer #2:

The manuscript "Forests in protected areas worldwide face increased thermal pressure from urbanization" submitted for publication consideration to Nature Communications provides a global spatial assessment of the distribution of urban settlement areas and protected forest areas. The results show that protected forest areas located closer to urban settlements have on average elevated surface temperatures. The research is interesting and shows clear spatial patterns. I do have some comments which should be addressed particularly concerning the overall argument for some of the analyses.

Authors: Thank you for your constructive comments which have helped us improve the manuscript. We addressed all the comments and made revisions according to your comments.

Introduction

Lines 54 ff...> The argument why it is important to understand how climate change and human activities affect these protected forests and their potentials to serve as thermal refuges for biodiversity is not entirely clear. The argument needs further elaboration. Also, climate change does not appear to be relevant in the further analyses.

Authors: We rephrased the sentences to better reflect the focus of this research.

With large-scale afforestation efforts underway, such as the United Nations Strategic Plan for Forests (UNSPF) and the Bonn Challenge, it is increasingly important to assess whether protected forests across different latitudes can effectively maintain their thermal buffering capability. (L55-58)

These human-induced changes in protected forests modify surface energy budgets and are often linked to local temperature anomalies. As a result, protected forests tend to experience increased microclimate instability, weakening their capacity to serve as thermal refuges for biodiversity. (L71-75)

Lines 61f "...has led to ongoing forest loss and degradation in these PAs". Please provide more details and/or values to back up the argument.

Authors: We revised this sentence to provide more details.

However, the intensified human pressures induced by urbanization have also exacerbated logging pressure, habitat fragmentation, and disrupted canopy structure near or in PAs. (L69-71)

Lines 64f: "Many species depend on microclimates sustained by natural landscape, particularly PAs to function as thermal refuges under climate change," --> please

provide more details and examples of such species.

Authors: We added examples of such species according to your suggestion

Many species depend on microclimates sustained by natural landscapes, including thermally sensitive ectotherms such as amphibians and reptiles that rely on shaded, cooler habitats to avoid thermal stress. (L76-78)

Methodology

1990-2018 – time period chosen may be justified in a short sentence (although it is clear from the data available but also a short discussion on why this time period would be helpful)

Authors: We added a brief explanation to the time period chosen.

The period 1990-2018 was selected because the GUB dataset used for the urban extent analysis is only available for this time period. (L389-390)

Lines 373 ff: So different distance within the buffer are used to define the PUI?

Authors: We evaluated PUIs defined using different buffer distances. We also compared urban pressure, canopy degradation, and temperature changes in PUIs mapped by different buffer distances (Table S1 and Figure 3) to test whether the urban pressure and its effects diminish with distance from the urban area. We further clarified in the last paragraph of the Introduction.

The urban pressure and its thermal impact analyses were conducted primarily at the 5km PUIs, while further analyses were conducted at the 10, 25 and 50km PUIs to assess the urban pressure and its thermal impact farther away from the urban areas. (L106-109)

A clear overview table should on data used with source, spatial resolution and temporal coverage should be provided

Authors: According to your suggestion, we added an overview data table in Supplementary Information.

All datasets used in the above analyses are summarized in Table S5. (L516)

Table S5 Overview of datasets used in this study

Dataset	Source	Spatial resolution	Temporal coverage
World Database on Protected	UNEP-WCMC /	Vector	2018

Areas	IUCN		
ESA CCI Land Cover	ESA	300m	1992, 2018
Global Urban Boundary	iEarth DataHub	Vector	1990-2018
Global Artificial Impervious Area	iEarth DataHub	30 m	1990-2018
Human Footprint Index	Mu et al.	1 km	2000-2022
SRTM30 DEM	USGS / NASA	1 km	Static
MODIS Aqua LST (MYD11A2)	USGS / NASA	1 km	2003-2018
MODIS LAI (MOD15A2H)	USGS / NASA	500 m	2001-2018
ESA CCI Biomass	ESA	100 m	2020
GLAD Global LCLUC	University of Maryland	30 m	2020
Global Mean Species Abundance	GLOBIO	300m	2015

Results

the analysis was done focusing on countries of lower and higher human development level. Why this was done, i.e. the argument behind is not entirely clear and should be developed in the introduction

Authors: We have removed the limited results related to human development level, as they had minimal impact on the main conclusions and did not substantially affect the overall interpretation of the result.

the analysis was also done to show differences between different forest types (tropical, temperate, boreal). Why this is important has not entirely been introduced and the argument needs further development in the introduction. Why is that important in the context of biodiversity.

Authors: We added related content in the introduction.

Tropical protected forests tend to exhibit stronger local cooling, whereas boreal protected forests play a greater role in slowing local warming trends. With large-scale afforestation efforts underway, such as the United Nations Strategic Plan for Forests (UNSPF) and the Bonn Challenge, it is increasingly important to assess whether protected forests across different latitudes can effectively maintain their thermal buffering capability. (L53-58)

Discussion

Is distance the decisive factor or is it size and density of the urban area located close to the PA --> so what role plays size, building-density, industry, traffic? What about

additional factors and the related impact on biodiversity?

Authors: Urban pressure is calculated as a nonlinear function of urbanization intensity and distance to urban areas (Equation 1 in Materials and Methods), which quantifies the pressure with general urban spatial patterns rather than the building-density, industry and traffic. We added further analysis and found that increased urban intensity plays a higher role than the distance to urban area in the urban pressure.

To examine temporal changes in urban pressure, we calculated ΔUP changes between 1990 and 2018 (ΔUP_{2018}-ΔUP_{1990}). Because urban pressure is defined as a nonlinear function of urbanization intensity and distance to urban areas, we conducted an additional scenario-based analysis to examine the relative roles urbanization intensity and distance to urban area. We recalculated ΔUP_{2018} under two scenarios: (S1) retaining only pre-existing urban pixels (urban intensity > 0 in 1990), (ΔUP_{2018}-ΔUP_{1990}) reflects changes associated with increasing urbanization intensity, and (S2) retaining only newly emerged urban pixels (urban intensity = 0 in 1990 and > 0 in 2018), (ΔUP_{2018}-ΔUP_{1990}) reflects distance reduction due to urban expansion. In both cases, ΔUP1990 was calculated using the urban distribution and intensity of 1990. We showed changes in ΔUP (ΔUP_{2018}-ΔUP_{1990}) were 1.02% and 0.73%, for scenarios S1 and S2, respectively. This analysis indicates that changes associated with urbanization intensity cause a larger ΔUP change than distance reduction alone. (L462-474)

We also added related discussions.

The results offer observational evidence that, at the global level, between 1990 and 2018, increasing urban pressure on PAs is driven not only by urban expansion toward PAs, but also by rising urban intensity in surrounding areas. Higher urban intensity reflects rapid changes in urban land use, including increased building density, expanded transport networks, and more concentrated industrial and residential activities. Increased industrial facilities and transport infrastructures can transfer waste heat and pollutants into nearby PAs through atmospheric advection and diffusion, while growing urban populations raise the frequency and intensity of human activities such as tourism and accommodation development, further increasing pressure on PAs. (L218-227)

Particularly for forest areas, the surface temperature as the major indicator might not entirely capture the climatic temperature environment as air temperature is much more impacting the local environment conditions relevant for local biodiversity, no? A critical reflection on the use of surface temperature as a main indicator is needed.

Authors: We agree with you that surface temperature and air temperature are not equivalent. Satellite observations mainly represent canopy surface temperature, rather

than air temperature, as clarified in the Methods (L487-495). Given the current limitations in obtaining globally consistent, high-resolution gridded air temperature data, particularly sub-canopy temperature observations, satellite-derived land surface temperature remains a widely used proxy in large-scale studies. We further discuss this limitation of the study.

The satellite-derived land surface temperature used in this study is a critical proxy of forest-environment interactions, however, it much infers the thermal conditions in the upper canopies. Observations of microclimate within canopies of different forest types remain limited, underscoring the need for field measurements and validations. (L305-309)

Lines 295ff: "Many tropical ectothermic species have evolved within narrow thermal ranges, often less than 2 °C between their realized habitat temperatures and upper thermal limits" can this be further explained using specific examples?

Authors: We added specific examples according to your suggestion.

For example, lowland tropical Anolis lizards and several tropical Drosophila species already have been subjected to temperatures approaching their critical thermal maxima, leaving warming tolerances of less than 2 °C. (L316-318)

A transparent discussion on the limitations of the approach is missing.

Authors: We added some discussions on the limitations of the study.

In this study, we disentangled the indirect impacts of urban heat spillover using a residual-based approach, however, observations and simulations are needed to better understand the underlying physical processes. (L291-293)

The satellite-derived land surface temperature used in this study is a critical proxy of forest-environment interactions, however, it much infers the thermal conditions in the upper canopies. Observations of microclimate within canopies of different forest types remain limited, underscoring the need for field measurements and validations. (L305-309)

When habitat fragmentation and heat stress occur simultaneously, they can reinforce each other and increase the risk of local extinction. However, these non-linear superposition effects are overlooked in the current studies. (L336-338)

Reviewer #3:

Title: Forests in protected areas worldwide face increased thermal pressure from urbanization

This study presented a research gap related to the consequences of urbanization on biodiversity conservation worldwide. Multiple analyses were conducted to assess how urbanization expansion is affecting protected areas in the different regions of the world. In general, the manuscript is very well written and clear. However, from our perspective, the manuscript fails to consider important connections between the research gap, method design, and the discussion of the results. In the following paragraphs, we emphasize the improvements in the study, considering major and minor aspects:

Authors: We are grateful to your constructive comments which have helped us improve the manuscript. We addressed all the comments and made revisions according to your comments.

Research design

The manuscript presents, as the relevant research gap, that the thermal effects resulting from urbanization may affect biodiversity (lines 51-52; lines 75-79). However, the methods are not designed to evaluate biodiversity (species richness and diversity; species distribution) but the simple extent of protected areas (PAs). If the research gap is centered on biodiversity or the extent of structured habitat for species distribution, the methods should be designed to address this research question.

Authors: We agree with you that evaluation of biodiversity often uses the metrices of species richness and diversity, species distribution. However, in this study, we focus on how urbanization affects the thermal buffering capacity of protected forests at the global scale, rather than assessing biodiversity directly. Thermal buffering capacity of forest plays an important role in helping species cope with climate warming and is widely recognized as a key indicator relevant to biodiversity conservation. To avoid confusion, we revised the relevant descriptions in the Introduction to better align with the actual scope of this study.

…it is increasingly important to assess whether protected forests across different latitudes can effectively maintain their thermal buffering capability. (L56-58)

Despite growing recognition of urban heat spillover, existing studies quantifying temperature elevation extending from urban areas to their surrounding rural areas mainly focused on its implications for human health and urban-rural resilience36,41. However, the thermal impacts of urban heat spillover on natural and semi-natural ecosystems, particularly protected forest ecosystems, remain underexplored. (L90-94)

Database

The manuscript presents PAs as a proxy of forest habitats. The PAs present different vegetation cover around the world, and in some regions, especially in tropical regions, this cover provides a rich habitat for species distribution (such as ruprestrian grassland or even the cerrado in Brazil). Therefore, the results obtained for "forest habitats" can be overestimated, considering that not all PAs are covered only by forests.

Authors: We agree with you that PAs globally present different vegetation cover. In this study, all analyses are conducted exclusively on protected forest. Non-forest PAs were not included in the calculations. This approach enables accurate quantification for a single land-cover type. Accordingly, our conclusions only apply to PAs with forest cover which is clarified in the title of the manuscript. We also added a clarification in the Materials and Methods.

Our analysis focused on protected forest pixels and do not include non-forest ecosystems within PAs. The findings are therefore most applicable to forest-dominated PAs. (L381-383)

Moreover, the PAs from UNEP-WCMC represent different protected territories, including indigenous lands, conservation units that permit or not the presence of human occupation. The manuscript considers a unique category of PA that may not be realistic for the discussion of the results.

Authors: We agree that PAs are subject to different policy and management arrangements. In this study, however, we do not differentiate among management regimes, as management intensity and effectiveness are shaped by local political and socio-economic conditions that may change over time. We therefore assume that management intensity and effectiveness are similar between PUIs and nearby background PAs within each 0.25° window. We also added a clarification in the Materials and Methods.

We assumed that the background climate for paired PUIs and background PAs was similar in each 0.25° window. We did not differentiate management regimes of PAs. At this local to regional scale of 0.25° window, management intensity and effectiveness, which are shaped by local political and socio-economic conditions and shift over time, are also assumed to be similar for PUIs and nearby background PAs. (L440-444)

Methods

The manuscript presents a proximity analysis to evaluate the effects on biodiversity/ecosystems. However, it is not sufficient to provide concrete evidence on the topic by not providing a landscape analysis, connectivity, and fragmentation

assessment. The authors mention the size of PAs as an important aspect, but their connectivity is of most importance for biodiversity conservation. The formation of ecological corridors, even between small PAs, is relevant for conserving biodiversity and the integrity of ecosystems, and this could play an important role in designing urban PAs and assessing their thermal effects. Moreover, the proximity analysis may be insufficient to evaluate the effects on biodiversity, requiring, for example, the analysis of species distribution across PAs when assessing the thermal effects of urbanization.

Authors: This study focuses on quantifying the impacts of urbanization on the local thermal buffering capacity of protected forests, a biophysical indicator that is broadly relevant to ecosystem functioning and biodiversity, yet has not previously been assessed at the global scale.

We agree that the effects of urbanization on biodiversity may also involve landscape connectivity and species-specific responses beyond thermal processes. In response to this comment, we added additional analyses examining the impacts of urbanization on forest connectivity and mean species abundance in PAs. Our results show that more than 70% of forest habitats within PUIs exhibit lower connectivity, and more than 64% of forest habitats within PUIs have lower mean species abundance than in background PAs, highlighting the potential threats of urbanization to biodiversity in PAs.

Beyond the observed thermal degradation, more than 70% of forest habitats within PUIs exhibit lower connectivity (Supplementary Information Note S4, Figure S8a), and more than 64% of forest habitats within PUIs have lower mean species abundance than in background PAs (Figure S8b). When habitat fragmentation and heat stress occur simultaneously, they can reinforce each other and increase the risk of local extinction. However, these non-linear superposition effects are overlooked in the current studies. (L332-338)

Note S4 Reductions in forest connectivity and mean species abundance in PUIs

Urbanization not only elevates land surface temperatures but also alters landscape structure and biodiversity-related attributes of protected forests. We further quantified the impacts of urbanization on forest connectivity and mean species abundance (MSA) in PAs.

Forest connectivity was calculated based on the ESA CCI forest cover dataset aggregated to a spatial resolution of 0.01° for the year 2018. Connectivity was quantified following the method proposed by Grantham et al.3, which estimates the probability of functional connections among forest patches. We applied three dispersal

parameters (σ = 5, 20, and 50), representing short-, medium-, and long-distance dispersal processes, respectively. The final forest connectivity value was derived as the mean of the connectivity estimates across these three dispersal scenarios.

Global MSA data at 300 m spatial resolution were obtained from the Global Biodiversity Model4. MSA ranges from 0 to 1, where a value of 1 indicates a fully intact species assemblage and a value of 0 indicates the complete local extirpation of the original species assemblage. Due to the unavailability of MSA data for 2018, we used the temporally closest dataset from 2015, which includes terrestrial plants and warm-blooded vertebrates. The MSA data were subsequently aggregated to a spatial resolution of 0.01°.

In the same 0.25° window, we evaluated the differences in connectivity (ΔCON) and MSA (ΔMSA) of forest habitats between in PUIs and in background PAs. We constrained our analysis to windows where the elevation difference between PAs and PUIs was within ±500 m. We show that more than 70% of forest habitats within PUIs exhibit lower connectivity (ΔCON < 0) compared to background PAs, with an average ΔCON of -6.31±0.23% (Figure S9A). Similarly, more than 64% of forest habitats within PUIs have lower mean species abundance (ΔMSA<0) than those in background PAs, with an average ΔMSA of -2.41±0.18% (Figure S9B). Furthermore, we find that over 55% of protected forests are simultaneously affected by urbanization, experiencing rising surface temperatures (ΔST>0), decreased connectivity (ΔCON< 0), and declining MSA (ΔMSA<0). These results indicate that urbanization is making protected forests increasingly warmer and more isolated, thereby contributing to reductions in local species abundance.

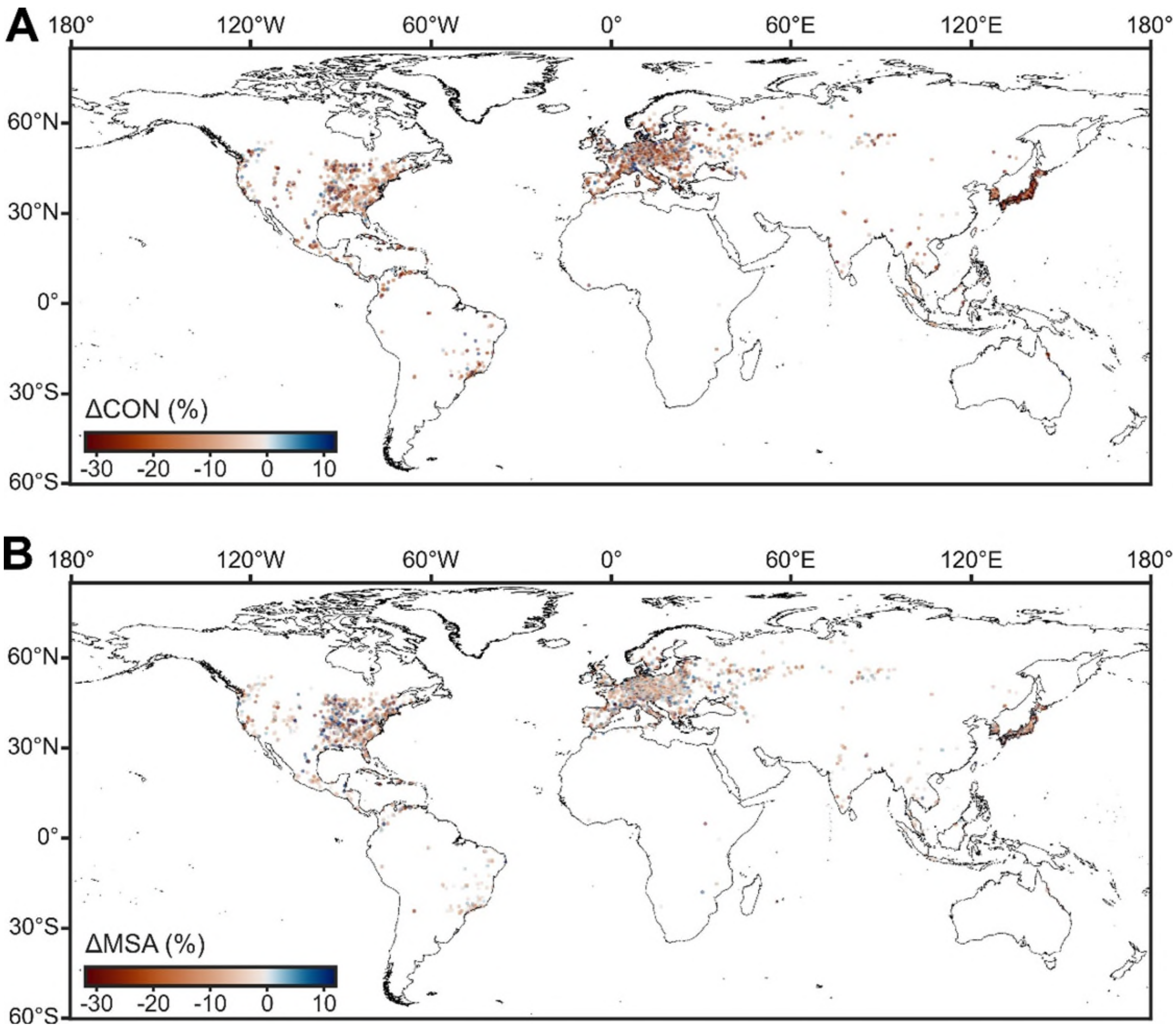

Figure S8 Reductions in forest connectivity and mean species abundance in PUIs. (A) Spatial distribution of t connectivity deficit (ΔCON) between protected forests in PUIs and in background PAs, and spatial distribution of mean species abundance (ΔMSA) between protected forests in PUIs and in background PAs

Minor comments

Introduction

The introduction fails to critically review the literature, considering aspects of ecosystem conservation close to urban areas. The discussion of cultural ecosystem services, such as recreation, environmental education provided by the PAs close to urban areas, needs to be described in the introduction as well.

Authors: We extended discussion on the ecosystem conservations close to urban areas and their cultural service in th introduction.

As urbanization approaches PAs, residents have easier access to ecosystem services, such as clean air and water, as well as cultural services obtained through recreation and environmental education. The ease of access to ecosystem services also fosters policy support and financial resources, thereby enhancing effectiveness of conservation efforts.

However, the intensified human pressures induced by urbanization have also exacerbated logging pressure, habitat fragmentation, and disrupted canopy structure near or in PAs. （L65-71）

Some very minor grammar adjustments are required (lines 44 and 64), and adding sources for the cited afforestation efforts in line 50.

Authors: We corrected the grammar error and added references.

Results

In general, it is not clear that the term ‘PAs’ refers only to the portion of protected areas that do not overlap with PUIs. I would recommend including an additional acronym for the PA area without PUIs. Readers might not be able to clearly distinguish this difference, as ‘PAs’ as an acronym for protected areas could refer to the whole PA area.

Authors: In the revised manuscript, we defined the portions of protected areas excluding PUIs as background PAs (PABs) and have revised the manuscript accordingly throughout the manuscript.

We further defined the portions of PAs excluding PUIs as background PAs (PABs). (L412-413)

When the attributions of surface temperature rise are presented (line 160), I would make the language tone used lighter, as there are certain limitations towards those findings.

Authors: We revised the attribution analysis (Supplementary Information Note S3) in response to your and other reviewers’ comments, and extended discussed on the limitations.

We further quantified the relative contributions of the direct effect (e.g., canopy degradation caused by urbanization) (Supplementary Information Note S2, Figure S4 and S5), indirect effect (e.g., urban heat spillovers), and residual component reflecting effects of topographic, edaphic, or other unaccounted factors to the thermal difference between PUIs and background PAs (Supplementary Information Note S3, Figure S6). We found that in the 0.38 ± 0.01 °C difference, about 0.18 °C is attributed to urbanization-induced direct forest canopy degradation, about 0.13 °C is attributed to indirect effects of urban heat spillover, and the remaining 0.07°C is introduced by the differences in factors such as background topography and soil properties between PUIs and background PAs (Supplementary Information Note S3). These results underscore the dual roles of direct forest degradation and indirect spillovers following urban expansion in shaping the surface temperature of forests in PUIs. (L171-182)

In this study, we disentangled the indirect impacts of urban heat spillover using a

residual-based approach, however, observations and simulations are needed to better understand the underlying physical processes. (L291-293)

Discussion

The discussion could describe the possible causal mechanisms between PA size and urban pressure.

Authors: We added related discussions.

We show that urban pressure in PUIs was higher in smaller PAs, whereas PUIs of larger PAs exhibited greater temporal increases in urban pressure since 1990. These contrasting patterns arise because small PAs are often located closer to cities (Supplementary Information Note S1) and thus experience higher baseline urban pressure, while further urban expansion around them is spatially and temporally constrained. Since the remaining non-PUI areas of small PAs are much smaller than those of larger PAs, small PAs are more likely to be fully exposed to urban pressure. In contrast, large PAs are typically located farther from cities with extensive boundaries, leaving greater potential for urbanization in their surrounding areas and leading to greater temporal increases in urban pressure as urbanization progresses. (L228-237)

The general consideration for policy implication should not be generic (as mentioned in the abstract) but grounded in the results.

Authors: We removed some broad policy statements and ensure the discussion on implications directly supported by the findings in this study.

Natural areas located less than 5km from urban areas experience the strongest urban and thermal pressures, and are therefore not recommended as priority locations for future PAs. The urban-induced warming in protected forest habitats can extend up to 50 km from city boundaries, suggesting that conservation planning must account for urban influence at larger spatial scales. In addition, our results show that protected forests exposed to urban-induced thermal risk are also losing connectivity, which may limit the ability of species in these regions to move and track suitable climates. A practical solution is to establish ecological corridors connecting these isolated PAs, forming a larger conservation network to enhance resilience of PAs against climate change. (L349-358)

Furthermore, presenting limitations of the work and future research directions would enhance the study.

Authors: We added discussion on the limitations of the work.

In this study, we disentangled the indirect impacts of urban heat spillover using a residual-based approach, however, observations and simulations are needed to better understand the underlying physical processes. (L291-293)

The satellite-derived land surface temperature used in this study is a critical proxy of forest-environment interactions, however, it much infers the thermal conditions in the upper canopies. Observations of microclimate within canopies of different forest types remain limited, underscoring the need for field measurements and validations. (L305-309)

When habitat fragmentation and heat stress occur simultaneously, they can reinforce each other and increase the risk of local extinction. However, these non-linear superposition effects are overlooked in the current studies. (L336-338)

Methods

The methods section and figures are clear. Data sources are cited, and supplementary information provides further details about the analysis. As there are many different steps and parts of the analysis, it would help the reading experience to provide a figure with an overview of the different methodological steps taken.

Authors: According to your and another reviewer's comment, we added a diagram in Figure S1 to illustrate the main research steps.

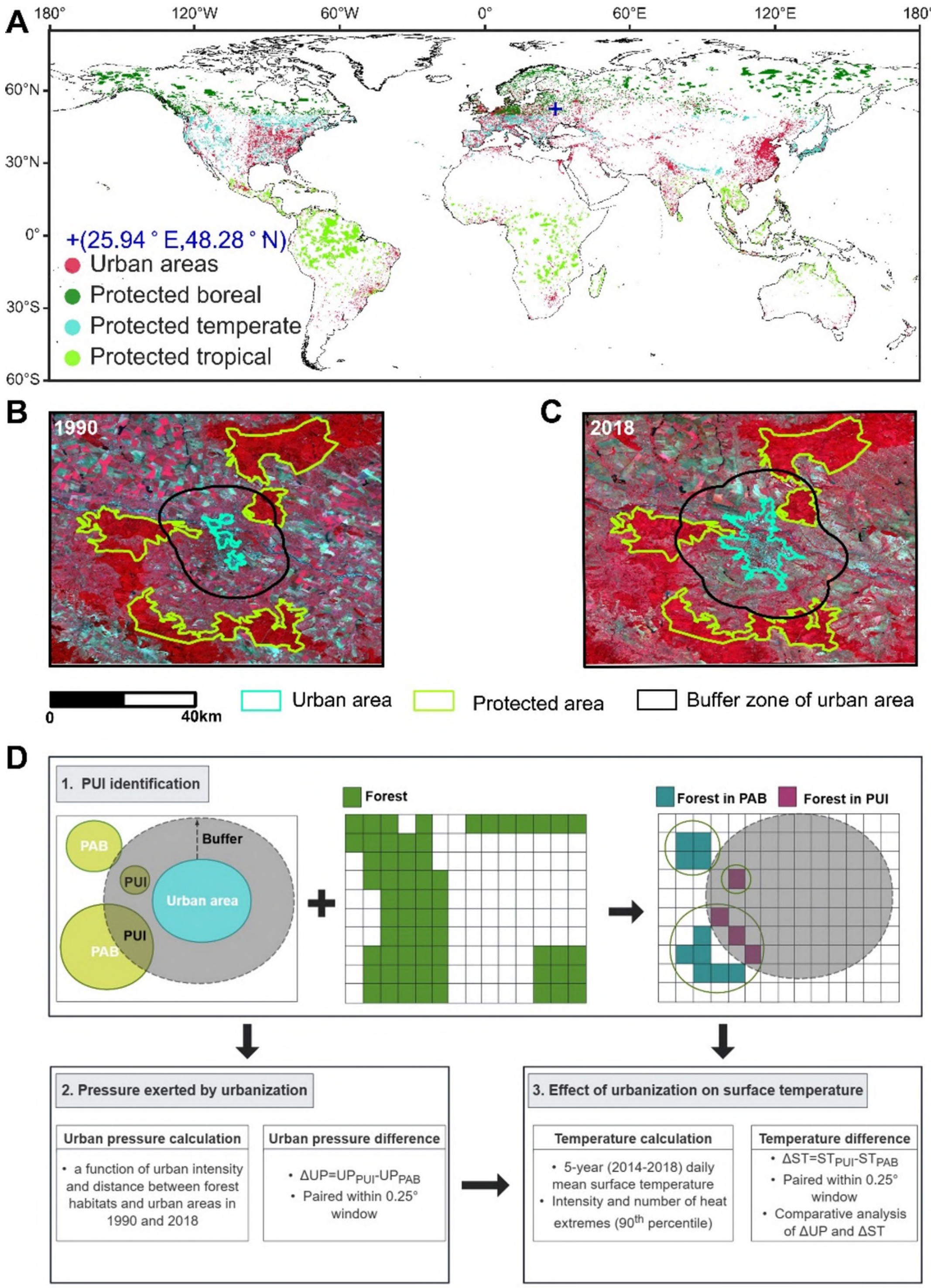

Figure S1 Global urban areas and protected forests. (A) Spatial distribution of global urban areas and protected boreal, temperate and tropical forests in 2018, (B) example of Landsat-5 false-color composite of near infrared, red, and green bands at 30-m resolution for urban areas, protected areas and buffer zone of urban area in Ukraine (25.94°E, 48.28°N) on August, 1990, (C) example of Landsat-8 false-color composite for the same area, on August, 2018, (D) diagram illustrating the main research steps to identify the protected area-urban interface (PUI) and their relative position with

background protected area (PAB), and calculate urban pressure (UP) and effect of urbanization on surface temperature (ST).

As in the results sections, we recommend substituting the acronym PA for a different one when referring to the portion without PUI.

Authors: We defined the portions of protected areas excluding PUIs as background PAs (PABs) and have revised the manuscript accordingly throughout the manuscript.

Moreover, the method used for attribution of surface temperature change presented in the results (line 160) is not cited in the methods. They should be at least cited in the 'Alteration of the thermal environment in forest habitats' section, including a reference to supplementary notes (note S3).

Authors: We added a reference to the Note S3.

Finally, if there are any limitations from the methods used and databases, they should be presented.

Authors: We added the limitations of the work concerning the methods and databases.

In this study, we disentangled the indirect impacts of urban heat spillover using a residual-based approach, however, observations and simulations are needed to better understand the underlying physical processes. (L291-293)

The satellite-derived land surface temperature used in this study is a critical proxy of forest-environment interactions, however, it much infers the thermal conditions in the upper canopies. Observations of microclimate within canopies of different forest types remain limited, underscoring the need for field measurements and validations. (L305-309)

When habitat fragmentation and heat stress occur simultaneously, they can reinforce each other and increase the risk of local extinction. However, these non-linear superposition effects are overlooked in the current studies. (L336-338)

Reviewer #4 (Remarks to the Author):

I co-reviewed this manuscript with one of the reviewers who provided the listed reports. This is part of the Nature Communications initiative to facilitate training in peer review and to provide appropriate recognition for Early Career Researchers who co-review manuscripts.

Authors: Thank you for taking your time to review our manuscript and provided valuable comments.

We appreciate the four reviewers for taking the time to review our manuscript and provide valuable comments. We addressed all the comments and made revisions in the manuscript accordingly. Please find our responses to the reviewers below. Our responses are in blue and the corresponding revisions in the manuscript are in purple. All the revisions are highlighted in yellow in the revised manuscript.

Reviewer #1:

Most of my previous comments have been addressed satisfactorily.

Authors: Thank you for taking your time to review our manuscript and provided valuable comments.

I have one additional comment:

I would be interested to know what the LST comparison would show if unprotected forests inside and outside the urban buffers were also analyzed. Moreover, what differences of LST exist between protected and unprotected forests within the urban buffer?

Authors: We added additional analyses on the LST comparison between unprotected forests inside and outside the urban buffers, and LST comparison between protected and unprotected forests within the urban buffer according to your suggestion. This result is added in the discussion.

We also found that within the 5-km urban buffer zone, protected forest habitats are 0.23 ± 0.01 °C cooler than unprotected forests, suggesting that forests within PUIs retain thermal buffering capacity despite urban warming. In addition, the temperature difference between unprotected forest habitats inside and outside urban buffer (5km) zones is 0.43 ± 0.01 °C higher than the temperature difference between protected forest habitats in background PAs and PUIs. This result implies that without forest conservation, thermal impact of urbanization is further amplified. (L362-369)

More minor comments:

L31: “surface temperature” is not equivalent to LST, please direct use LST.

Authors: We replaced the ST by LST throughout the manuscript.

L32 “mean surface temperature” is not clear, is annual mean values of daily LST?

Authors: We revised the wording as “annual mean daily LST”.

Reviewer #2:

The manuscript has improved and my previous comments have been addressed, mostly sufficiently.

Authors: Thank you for reviewing our manuscript and for your valuable comments.

Minor comments remain:

In the Supplementary Material: The Mu et al. Reference is not included in the Manuscript. The publication with Scientific Data covers 2000-2018. https://doi.org/10.1038/s41597-022-01284-8 Was there an update? Please add reference.

Authors: We have added the corresponding reference in the manuscript and clarified that the dataset has been updated to 2024.

I suggest you define Protected areas right at the beginning of the manuscript and clearly highlight that you mean in your case forest PA. Otherwise the use of the term and abbreviation raises confusion.

Authors: We clarified this at the end of the Introduction by explicitly stating that our analysis focuses on forests within protected areas and does not include non-forest protected areas.

To address this gap, we provide the global assessment of urbanization-induced thermal pressure on protected forest habitats. (L94-95)

By overlaying PUI and background PA boundaries with satellite-derived forest cover data, we only identify protected forest habitats in these regions. (L101-102)

Line 56: "Nations Strategic Plan for Forests (UNSPF) and the Bonn Challenge14,15" Can you please briefly explain what these efforts quantitatively mean, any thresholds to be reached?

Authors: We added quantitative description of targets of UNSPF and Bonn Challenge.

…such as the United Nations Strategic Plan for Forests (UNSPF), which aims to increase global forest area by 3% by 2030, and the Bonn Challenge, which targets the restoration of 350 million hectares of degraded and deforested land globally by 2030… (L54-57)

Line 107ff: You need to describe here clearly already why you primarily focus at the

5km buffer.

Authors: We added a brief explanation.

The urban pressure and its thermal impact analyses were conducted primarily at the 5km PUIs, where the urban effects are most pronounced (Table S1) … (L109-111)

Lines 202ff: When do you consider a PA as large and when small?

Authors: We clarified that small PAs (< 10 km²) and large PAs (> 1000 km²) in the revised manuscript.

For the discussion section, I suggest to write out abbreviations again at the beginning, just to remind the reader about their meaning.

Authors: We reintroduced the abbreviations for protected areas (PAs) and protected area-urban interfaces (PUIs) at the beginning of Discussion section.

• Line 230: Please specify “are often”. What do you mean by often?

Authors: We revised the relevant content and linked it to the analysis in Supplementary Information Note S1.

These contrasting patterns arise because small PAs tend to be located closer to cities. Based on the 1990 baseline analysis (Supplementary Information Note S1), 24.65% (40.92%) of small PAs are within 5 km (10 km) of urban areas, compared to only 5.24% (8.50%) for large PAs. (L240-244)

• Lines 305ff: The discussion on the limitation of the approach concerning the use of surface temperature as a proxy for temperature increase versus air temperature is too narrow and should be expanded. Please, include also references to back up your argument.

Authors: We expanded the discussion of surface temperature and air temperature and added relevant references.

The satellite-derived land surface temperature used in this study is a critical proxy of forest-environment interactions, however, it much infers the radiative temperature of the upper canopies[52]. Land surface temperature may differ from 2m air temperature, which is more commonly used in climate assessments[53]. Therefore, converting land surface temperature to corresponding air temperature responses is important but

remains challenging due to complex land-atmosphere interactions and atmospheric circulation[54,55]. Moreover, observations of air temperature within canopies of different forest types remain limited, underscoring the need for field measurements and validations[32]. Nonetheless, our analysis based on satellite-derived land surface temperature provides clear evidences of the thermal effects of urbanization on forest habitats within PAs (L306-316)

Please write out or explain abbreviations in the figure legends in your figures.

Authors: We checked and clarified abbreviations in the figure captions.

Reviewer #3:

Second review - General comments from the reviewers

The authors made several amendments to the manuscript. The research scope is clearer and better justified. The authors improved the clarity in the methods section. Additional analysis of impacts was conducted, and some limitations were clarified. However, we still identify some important issues, detailed below, among the three major topics.

Authors: We are grateful to your constructive comments which have helped us improve the manuscript. We addressed all the comments and made revisions according to your comments.

1) The research gap needs to ground the research design

Existing literature focused on temperature elevation; the authors argue that there is a gap in studies analyzing thermal impacts on protected landscapes. However, the authors only quantify the temperature change, without an analysis of the actual impacts derived from it (change in the environmental quality in the affected habitats).

Authors: We revised the Introduction to clarify that the study focuses on quantifying urbanization-induced thermal changes in protected areas, instead of broadly referring to ecological impacts.

Despite growing recognition of urban heat spillover, existing studies mainly focused on thermal conditions in human-dominated areas, whereas the magnitude and spatial extent of urban heat spillover into natural and semi-natural ecosystems remain poorly quantified. (L90-93)

2) Use of terms: Protected forests X PAs in general

Given the fact that the conducted analysis considered only forest pixels within protected areas (lines 381-383), the results cannot be directly extrapolated to the whole PA systems. The authors need to be more careful when concluding results, considering this limitation. For example, in lines 258 and 296 of the revised paper, results are cited as impacts on the whole PAs and not only on protected forests.

Authors: We revised the manuscript throughout to ensure that relevant results refer to protected forests, rather than to PAs as a whole.

3) The term 'background PAs' is confusing and cannot be used as a robust comparison for impact assessment

The authors argue that they analyze the impacts of urbanization on the local thermal capacity of protected forests. However, the thermal capacity or impact in PUI is not properly compared to control areas to demonstrate the real impact of thermal effects. The comparison with the "internal region" of each PA is biased by other cooling effects of dense forests and does not provide a robust threshold without the effects of urbanization and other pressures surrounding the PAs (See impact assessment and BACI methods)

The term 'background PAs' is confusing, because it treats the PAs without any overlap with urban areas, and those that do not suffer any effect from it in the same way as PAs with some overlap with urban areas. While PAs farther from urban areas and/or with similar properties could be treated as a control group for a robust assessment, this is not done.

The authors compared biodiversity indicators (connectivity and mean of species abundance) only between PUIs and background PAs. This represents an issue, because PAs that do not overlap with urban areas (without PUIs) and PAs that partially overlap with urban areas are considered the same.

Authors: Thank you for this important comment. Our analysis is conducted within paired 0.25° windows to ensure comparable climate backgrounds. Within each window, background PAs represent areas under low urban pressure, rather than areas free from urban pressure (UP = 0). Accordingly, we quantified urban pressure across all paired windows and found that forests in PUIs experience higher urban pressure ($3.20 \pm 0.06\%$) than those in background PAs ($0.64 \pm 0.01\%$) in 2018.

Across all paired windows, forests in background PAs with no urban pressure (UP = 0) account for only a small fraction (~5%) of total forests in background PAs globally. Among these, only 58% are located within protected areas that do not overlap with urban buffer zones.

Taken together, this indicates that, under current urbanization, even forests in background PAs are subject to non-zero urban pressure, and truly unaffected control areas near cities are rare. Therefore, rather than relying on idealized control-based

designs, our framework quantified urban influence as a relative difference between PUIs and background PAs, and better reflects real-world conditions.

We included this quantitative justification in the methodology.

Disturbance caused by urbanization on forests in background PAs was assumed to be lower. Forests in PUIs, however, were assumed to be exposed to more external disturbances caused by urbanization than protected forests in paired background PAs. Accordingly, across all paired 0.25° windows, forests in PUIs experience a higher urban pressure (3.20 ± 0.06%) than that in background PAs (0.64 ± 0.01%) in 2018. Forests in background PAs without urban pressure (UP = 0) account for less than 5% of forests in background PAs globally. Therefore, we quantify urban influence as a relative difference between PUIs and background PAs. (L470-478)

The authors directly attribute the differences in connectivity and species abundance to the urban effects, without considering other drivers for it, such as other human disturbances (infrastructure projects, mining, agriculture) and the impacts of the edge effect itself, where the interior of PAs will naturally be more conserved and suffer less from any disturbances than the edges.

Authors: We agree that multiple human disturbances, including infrastructure development, agriculture, and industrial activities, can influence connectivity and species abundance. However, these disturbances are not treated independently from urbanization, as they are often closely associated with urbanization. In particular, within the paired 0.25° windows used in this study, which are located near urban areas, it is difficult to disentangle whether such disturbances are driven directly by urbanization or occur as co-located processes.

We clarified this point in the revised manuscript and interpret the observed differences as reflecting the combined effects of urbanization and associated human pressures.

The observed differences are assumed to reflect the combined influence of urbanization and associated human activities (e.g., infrastructure, agriculture, and industrial activities). (Supplementary Information Note S4 L172-174)

Based on our definitions of PUIs and background PAs, both categories include interior and edge regions, and background PAs are not restricted to interior areas only. To further

evaluate the potential influence of edge effects, we conducted an additional analysis by excluding forest pixels within 1 km of PA boundaries. The results show that the mean values of ΔCON (−6.12 ± 0.15%) and ΔMSA (−2.24 ± 0.11%) change only slightly compared to the main analysis. This indicates that edge effects have a limited influence on the comparison and do not substantially alter the observed differences.

To assess the influence of edge effects, we excluded forest pixels within 1 km of PA boundaries and found that the mean values of ΔCON (−6.12 ± 0.15%) and ΔMSA (−2.24 ± 0.11%) changed only slightly, indicating a limited impact of edge effects on the results. (Supplementary Information Note S4 L185-188)

Furthermore, the urban effect on the thermal impact needs to be isolated from other drivers of temperature change. The temperature change cannot be fully attributed to urban effects, because there are also thermal effects from other drivers, such as the edge effect due to fragmentation of the landscape and other human disturbances in the surroundings.

Because of these important topics explained above, we recommend a major review to address those issues in the analysis conducted.

Authors: We agree with you that urban effects encompass both urbanization itself and associated human activities (e.g., infrastructure development, agriculture, and industrial activities), as well as landscape fragmentation. Therefore, we decomposed the thermal response into two components: (i) direct effects associated with forest canopy degradation and (ii) indirect effects related to urban heat island (UHI) spillover. These processes are not independent. Human activities and landscape fragmentation can influence temperature through both pathways, by directly altering canopy structure and indirectly enhancing UHI.

To address your comments and further examine these effects, we conducted additional analyses to assess the relationships between temperature differences and multiple factors, including differences in population density (ΔPOP), road density (ΔRD), agricultural intensity (ΔAG), impervious surface density (ΔIS), and forest edge density (ΔED). The analysis is added in the results section and the method is added in the supplementary information Note S3 Attribution of urbanization-induced surface warming.

Partial correlation analysis shows that the direct thermal effect of urbanization in PUIs is mainly contributed by population density (r=0.31, $p<0.01$), forest edge density (r=0.23, $p<0.01$), and impervious surface density (r=0.21, $p<0.01$), which exerts pronounced pressure on canopy structures. The indirect effect of urbanization is dominated by impervious surface density (r=0.39, $p<0.01$) followed by population density (r=0.21, $p<0.01$), indicating their major role in shaping the strength of urban heat spillover (Supplementary Information Note S3, Table S3). (L183-190)

Note S3 Attribution of urbanization-induced surface warming (L131-151)

We further chose six explanatory variables including differences in population density (ΔPOP), road density (ΔRD), agricultural density (ΔAG), impervious surface density (ΔIS), edge density (ΔED) between forests in PUIs and background PAs within each 0.25° grid cell. The population density, road density, agricultural density, impervious surface density are from LandScan Global Gridded Population Dataset, Global Roads Inventory Project Dataset, Global Artificial Impervious Area Dataset and GLAD Global Land Cover and Land Use Change Dataset, respectively, and are aggregated to a 0.01° resolution. Forest edge density was defined as the ratio of total edge length to window area within each 0.01° grid cell, and was calculated using the GLAD Global Land Cover and Land Use Change dataset. We used Ordinary Least Squares (OLS) model to calculate the partial correlation coefficients of these explanatory variables with direct (ΔLST_{direct}) and indirect ($\Delta LST_{indirect}$) land surface temperature difference. To ensure the robustness of the results, we ran the OLS models by randomly subsampling 30% samples with replacement out of the entire data 500 times and calculated the mean partial correlation coefficient for each explanatory variable across all model runs. Compared with forests in background PAs (Table S3), forests in PUIs show direct warming associated with population density, forest edge density, and impervious surface density, suggesting more pronounced canopy degradation under these pressures. Indirect warming is dominated by impervious surface density and population density, indicating that these factors play a major role in shaping the strength of urban heat spillover.

Table S3 Partial correlation of drivers for direct and indirect thermal effects

Drivers	Direct effect (r)	Indirect effect (r)
ΔPOP	0.311 **	0.211 **
ΔIS	0.211 **	0.391 **
ΔED	0.227 **	0.187 **
ΔAG	0.115 *	0.081 ^{ns}
ΔRD	0.108 ^{ns}	0.068 ^{ns}

'*' and '**' indicate $p < 0.05$ and $p < 0.01$, respectively, and ns indicates not significant. ΔPOP, ΔRD, ΔAG, ΔIS, and ΔED denote differences between forest in PUIs and background PAs in population density, road density, agricultural intensity, impervious surface density, and edge density, respectively.

Reviewer #4:

I co-reviewed this manuscript with one of the reviewers who provided the listed reports. This is part of the Nature Communications initiative to facilitate training in peer review and to provide appropriate recognition for Early Career Researchers who co-review manuscripts.

Authors: Thank you for taking your time to review our manuscript and provided valuable comments.

We appreciate the four reviewers for taking the time to review our manuscript and provide valuable comments. We addressed all the comments and made revisions in the manuscript accordingly. Please find our responses to the reviewers below. Our responses are in blue and the corresponding revisions in the manuscript are in purple. All the revisions are highlighted in yellow in the revised manuscript.

Reviewer #1 (Remarks to the Author):

My previous comments have been generally addressed. However, I still have the following concerns that may need to be further clarified or strengthened, and hopefully that can help improve the study.

Authors: We are grateful to your constructive comments which have helped us further improve the manuscript. We addressed all the comments and made revisions according to your comments.

1. Conceptual ambiguity and imprecise terminology

Some key concepts and terms used in the manuscript remain ambiguous or insufficiently rigorous. First, the manuscript interprets the local land surface (i.e., canopy) cooling effect of forests as "thermal refugia" or "thermal buffering capacity." This interpretation is not entirely accurate. In ecological and climatological contexts, these terms typically refer to the ability of forests to regulate sub-canopy microclimate and buffer against ambient temperatures, including both cooling under hot conditions and warming under cold conditions (see reference like this: https://www.nature.com/articles/s41559-019-0842-1). However, MODIS LST represents canopy surface temperature and does not capture the microclimatic conditions beneath the forest canopy. Therefore, using these terms in the current context may be conceptually misleading and should be reconsidered or more carefully qualified.

Authors: We clarified at the end of the Introduction that LST is used as a proxy for forest microclimate temperature, supported by relevant references.

Because global gridded observations of sub-canopy microclimate temperature remain limited[42], we use satellite-derived land surface temperature (i.e., canopy surface temperature of forests) as a proxy for forest microclimate temperature, an approach supported by field evidences reporting a strong positive correlation between canopy surface temperature and sub-canopy microclimate temperature[43-45]. (L97-102)

43 Wei, L. et al. Relationships between canopy surface temperature measured from drones and below-canopy forest microclimate in a tree diversity experiment. International Journal of Biometeorology 70, 118 (2026).
44 Zellweger, F., De Frenne, P., Lenoir, J., Rocchini, D. & Coomes, D. Advances in microclimate ecology arising from remote sensing. Trends in ecology & evolution 34, 327-341 (2019).
45 Farella, M. M., Fisher, J. B., Jiao, W., Key, K. B. & Barnes, M. L. Thermal remote sensing for plant ecology from leaf to globe. Journal of Ecology 110, 1996-2014 (2022).

Second, terms such as “macroclimate warming” and “microclimatic conditions” are used somewhat loosely. These terms should be clearly defined and used consistently.

Authors: We clarified these terms by providing explanations at their first occurrence. Specifically, we define “microclimate” as local climatic conditions and “macroclimate” as large-scale climatic conditions, and revised the sentence accordingly.

Third, expressions such as “human pressure,” “urban pressure,” and “thermal pressure” are vague and potentially ambiguous. These terms would benefit from clearer definitions or replacement with more precise, measurable descriptors.

Authors: We replaced “human pressure” with “human activities” to make the expression more straightforward. “Urban pressure” is a calculated metric in this study, and we defined it when it first appeared in the manuscript (L113-115). In addition, we defined “thermal pressure” at its first appearance as temperature anomalies relative to the background climate (L96-97).

2. Attribution analysis lacks depth and clarity

While I generally agree that the impacts of urbanization on LST in PUIs can be attributed to both direct human disturbance and urban heat spillover, the attribution analysis presented in the main text (L174–190) remains insufficiently convincing. The quantitative decomposition (e.g., 0.18 °C attributed to canopy degradation, 0.13 °C to urban heat spillover, and 0.07 °C to background differences) seems interesting, but the methodological basis for this partitioning is not adequately explained. The authors are encouraged to provide a more detailed explanation of the attribution framework, including assumptions, uncertainties, and limitations, to strengthen the robustness and interpretability of these results.

Authors: We have revised the manuscript to clarify the attribution framework in the Supplementary Information and an expanded discussion of uncertainties and limitations

in the Discussion section.

To isolate the contribution of urbanization to warming of forest, we assume that, under the same background climate, differences in forest LST between PUIs and background PAs are primarily associated with urbanization-driven human disturbance. (SI L79-91)

This decomposition assumes that urban pressure influences LST through both local biophysical changes in forest canopy structure and non-local UHI spillover from surrounding urban areas, which can be approximated as additive contributions at the grid scale. (SI L98-101)

In this study, the UHI spillover is not directly quantified but inferred as the residual after subtracting the direct canopy effects from total urbanization-induced thermal effects. This residual represents a combination of physical processes regulating heat transport and redistribution, including urban morphology, wind patterns, and atmospheric circulation. The approach assumes a linear response of land surface temperature to urban pressure and treats canopy degradation and UHI spillover as additive effects without explicitly accounting for their interactions, which can introduce uncertainty into the attribution results. However, forest canopy and UHI effects interactions (e.g., UHI spillover influencing canopy structure) cannot be disentangled with current observations. (L308-317)

3. Urban heat spillover is not explicitly quantified

The manuscript introduces urban heat spillovers as a key mechanism, but it is not directly quantified. Instead, it appears to be inferred as the residual after subtracting the effects of direct canopy degradation from the total LST difference. Moreover, urban heat spillover is described as driven by atmospheric processes such as advection and diffusion (L87-88 in the main text), yet no analysis of wind patterns, atmospheric circulation, or related variables is included to support this mechanism. At a minimum, this limitation should be explicitly acknowledged in the Discussion. Clarifying that the current estimation of urban heat spillover is indirect and subject to uncertainty would improve the transparency and credibility of the study. Overall, addressing the above issues is needed to improve its conceptual rigor and the robustness of its conclusions.

Authors: We revised the manuscript to include an explicit discussion of the limitations associated with the indirect attribution of UHI spillover effects.

In this study, the UHI spillover is not directly quantified but inferred as the residual after subtracting the direct canopy effects from total urbanization-induced thermal effects. This residual represents a combination of physical processes regulating heat transport and redistribution, including urban morphology, wind patterns, and atmospheric circulation. The approach assumes a linear response of land surface temperature to urban pressure and treats canopy degradation and UHI spillover as additive effects without explicitly accounting for their interactions, which can introduce uncertainty into the attribution results. However, forest canopy and UHI effects interactions (e.g., UHI spillover influencing canopy structure) cannot be disentangled with current observations. In addition, some inferred UHI spillover effects may reflect indirect warming from industrial activities and infrastructure development outside city boundaries through canopy degradation and anthropogenic heat emissions, which are not separately resolved in our analysis. This gap highlights the need for nonlinear attribution models by integrating interpretable machine-learning approaches with numerical models, e.g., urban canopy models, while incorporating multiple anthropogenic processes to enable a more comprehensive attribution of urbanization effects on protected forest thermal environment. (L308-324)

Reviewer #2 (Remarks to the Author):

My point on the reference data for the human foodprint index was not solved and still the old reference is in the manuscript although it is argued that the data was updated to 2024. In the supplementary Material, the Mu et al. reference appears in Table S6 without indication of year and the reference is not included at all in the supplementary Material. Also, in Table S6 it is called index but authors use intensity.

In the manuscript the old reference with No 42 is still used: Mu, H. et al. A global record of annual terrestrial Human Footprint dataset from 2000 to 2018. Scientific Data 9, 176 (2022).

I am very surprised that this was not solved during revisions.

Authors: We are grateful to your comments. The Human Footprint intensity used in this study has been continuously updated through the public database, without peer-reviewed publications reporting the dataset for extended years. We have therefore retained the original citation (Mu et al., 2022) while adding an explicit reference to the updated dataset and its access link. In addition, we revised the Supplementary Material to include the new reference and changed "index" to "intensity" to maintain consistency with the main text.

Mu, H. et al. An annual global terrestrial Human Footprint dataset (Version 8) [Dataset]. figshare. https://doi.org/10.6084/m9.figshare.16571064.v8. (2025).

Reviewer #3 (Remarks to the Author):

The authors made a comprehensive review, addressing most of the topics we have pointed out. The additional analyses made on the multiple possible drivers for temperature change enhanced the robustness of the study. Before considering the manuscript for final acceptance, we suggest only minor reviews.

Authors: We are grateful to your positive comments. We addressed all the comments and made further revisions according to your comments.

(i) the inclusion of a separate session on limitations of the work, describing limitations such as the difficulty of separating the influence of urban island effect from other activities such as industries and infrastructure projects on the impacts on PAs described;

Authors: We added a separate paragraph discussing the limitations associated with the indirect attribution of UHI spillover effects, and further expanded the discussion on the limitations of the attribution approach.

In this study, the UHI spillover is not directly quantified but inferred as the residual after subtracting the direct canopy effects from total urbanization-induced thermal effects. This residual represents a combination of physical processes regulating heat transport and redistribution, including urban morphology, wind patterns, and atmospheric circulation. The approach assumes a linear response of land surface temperature to urban pressure and treats canopy degradation and UHI spillover as additive effects without explicitly accounting for their interactions, which can introduce uncertainty into the attribution results. However, forest canopy and UHI effects interactions (e.g., UHI spillover influencing canopy structure) cannot be disentangled with current observations. In addition, some inferred UHI spillover effects may reflect indirect warming from industrial activities and infrastructure development outside city boundaries through canopy degradation and anthropogenic heat emissions, which are not separately resolved in our analysis. This gap highlights the need for nonlinear attribution models by integrating interpretable machine-learning approaches with numerical models, e.g., urban canopy models, while incorporating multiple anthropogenic processes to enable a more comprehensive attribution of urbanization

effects on protected forest thermal environment. (L308-324)

(ii) review in the language and final tone of the manuscript, aligned with these limitations.

Authors: We checked the manuscript throughout to ensure that the presentation is aligned with these limitations.

Reviewer #4 (Remarks to the Author):

I co-reviewed this manuscript with one of the reviewers who provided the listed reports. This is part of the Nature Communications initiative to facilitate training in peer review and to provide appropriate recognition for Early Career Researchers who co-review manuscripts.

Authors: Thank you for taking your time to review our manuscript and provided valuable comments.

Reviewer #1 (Remarks to the Author):

I have carefully reviewed the authors' responses and the revised manuscript and I am satisfied with the revisions made, as they have adequately addressed the concerns raised in the previous rounds of review. Therefore, I am pleased to recommend this paper for publication in its current form.

Authors: We sincerely thank you for your positive feedback and recommendation for publication of our manuscript.

Reviewer #2 (Remarks to the Author):

My comments were addressed. Thanks.

Authors: We sincerely thank you for your positive feedback and are pleased that our revisions have addressed your comments.

Reviewer #3 (Remarks to the Author):

The authors added a paragraph describing the limitations of the work, addressing the main recommendation from the previous round of review.

A minor point of review is the need to define the new acronym - UHI. From our point of view, after this small change, the manuscript can be considered for publication.

Authors: We sincerely thank you for your careful review and constructive suggestions. We have now defined the acronym UHI (urban heat island) at its first occurrence in the Introduction.

Reviewer #4 (Remarks to the Author):

I co-reviewed this manuscript with one of the reviewers who provided the listed reports. This is part of the Nature Communications initiative to facilitate training in peer review and to provide appropriate recognition for Early Career Researchers who co-review manuscripts.

Authors: Thank you for taking the time to review our manuscript and for your valuable feedback.
